# Gene Expression Analysis Indicates Divergent Mechanisms in DEN-Induced Carcinogenesis in Wild Type and Bid-Deficient Livers

**DOI:** 10.1371/journal.pone.0155211

**Published:** 2016-05-19

**Authors:** Changshun Yu, Shengmin Yan, Bilon Khambu, Xiaoyun Chen, Zheng Dong, Jianhua Luo, George K. Michalopoulos, Shangwei Wu, Xiao-Ming Yin

**Affiliations:** 1 Division of Clinical Microbiology, School of Laboratory Medicine, Tianjin Medical University, Tianjin, China; 2 Department of Pathology and Laboratory Medicine, Indiana University School of Medicine, Indianapolis, Indiana, United States of America; 3 Kingmed Center for Clinical Laboratory, Guangzhou, China; 4 Department of Cellular Biology and Anatomy, Medical College of Georgia and Charlie Norwood VA Medical Center, Augusta, Georgia, United States of America; 5 Department of Pathology, University of Pittsburgh School of Medicine, Pittsburgh, Pennsylvania, United States of America; UC Davis Comprehensive Cancer Center, UNITED STATES

## Abstract

Bid is a Bcl-2 family protein. In addition to its pro-apoptosis function, Bid can also promote cell proliferation, maintain S phase checkpoint, and facilitate inflammasome activation. Bid plays important roles in tissue injury and regeneration, hematopoietic homeostasis, and tumorigenesis. Bid participates in hepatic carcinogenesis but the mechanism is not fully understood. Deletion of Bid resulted in diminished tumor burden and delayed tumor progression in a liver cancer model. In order to better understand the Bid-regulated events during hepatic carcinogenesis we performed gene expression analysis in wild type and *bid*-deficient mice treated with a hepatic carcinogen, diethylnitrosamine. We found that deletion of Bid caused significantly fewer alterations in gene expression in terms of the number of genes affected and the number of pathways affected. In addition, the expression profiles were remarkably different. In the wild type mice, there was a significant increase in the expression of growth regulation-related and immune/inflammation response-related genes, and a significant decrease in the expression of metabolism-related genes, both of which were diminished in *bid*-deficient livers. These data suggest that Bid could promote hepatic carcinogenesis via growth control and inflammation-mediated events.

## Introduction

Bid (BH3 interacting domain death agonist) is a Bcl-2 family protein with multiple functions [1]. It was first cloned based on its interaction with Bcl-2, the founding member of the family and was soon found to play a pro-apoptosis role, antagonizing the pro-survival effect of Bcl-2 [2]. This effect requires a functional domain, the BH3 domain [2], which is shared by most Bcl-2 family proteins [3]. Bid is cleaved by caspase-8 following the activation of death receptors, which removes the N-terminal inhibitory domain, and enables the truncated Bid (tBid) to translocate to the mitochondria for the release of apoptogenic factors [4–6]. Deletion of Bid renders the mice resistant to hepatocyte apoptosis and liver injury caused by death receptor activation [7–9]. The effect of Bid on the mitochondria also promotes mitochondria-inflammasome signaling and thus provides a mechanism for inflammasome activation [10].

Like several other Bcl-2 family proteins, Bid also possesses a function regulating cell proliferation [11]. Deletion of Bid delays resting cells from entering cell cycle upon mitogenic stimulation [12]. In hepatocytes, this pro-proliferation function of Bid seems to be related to the ability of full length Bid to localize to the endoplasmic reticulum (ER), where it enhances the release of calcium [13]. Another function of Bid is related to genomic stability. Bid can be a substrate of ATM/ATR and plays a role in S phase checkpoint following DNA damage [14, 15]. These diverse functions enable Bid to play important roles in a number of pathophysiological processes including liver injury [7–9], hematopoietic homeostasis [16–18], liver regeneration [13], and cancer development [12, 19].

Neoplasia is characterized by uncontrolled cell proliferation and/or deregulated cell death. Bcl-2 family proteins may affect tumorigenesis by either mechanism. As these activities could have opposite effects on tumor development, the net impact of the Bcl-2 family proteins on tumorigenesis could be determined by the relative strength of these activities, which can be affected by factors such as etiology, tissue type, and tumor microenvironment [20]. Thus the Bcl-2 family proteins tend to affect tumorigenesis in the lymphoid cells mainly by regulating apoptosis [21], but their effects on liver cancers may be more related to their ability to regulate cell proliferation [12, 19, 22–24]. Thus while over-expression of Bcl-2 promotes lymphoma by suppressing apoptosis [21], the same maneuver suppresses carcinogen-induced hepatocellular carcinoma (HCC), likely by suppressing proliferation [22, 23]. On the other hand, *bid*-deficient mice develop spontaneous chronic myelomonocytic leukemia when aged [18]. This may be explained by the loss of the pro-death activity of Bid. Yet deletion of Bid does not result in enhanced HCC development induced by diethylnitrosamine (DEN) [12, 19], as it would be predicated from the diminished cell death, but results in diminished HCC development, which may be anticipated based on reduced pro-proliferation effect of Bid [12, 19]. Deletion of another BH3-only molecule PUMA also lead to a paradoxically reduced hepatic carcinogenesis [24]. The mechanisms of pro-death and pro-proliferation do not occur in isolation but could affect each other. For example, death of neighboring cells could trigger compensatory proliferation of other cells, which would be reduced if cell death is inhibited [25, 26]. It is likely that multiple mechanisms could be involved in the complex process of carcinogenesis.

We thus would like to determine whether the molecular events following DEN treatment could be different in mice with or without Bid. Toward this end, we performed gene expression and pathway analysis using microarray chips and found that such differences were indeed present between wild type and *bid*-deficient livers. These data provided new insights on DEN-induced hepatic carcinogenesis and further supported the notion that the absence of Bid altered the process.

## Materials and Methods

### Mice and treatment

Diethylnitrosamine (DEN, C_4_H_10_N_2_O,) was intraperitoneally administrated to male wild type and *bid*-deficient mice at the age of day 15 as previously described [12]. Wild type mice were in C57Bl/6J background whereas *bid*-deficient mice had been backcrossed to C57Bl/6J background for at least 12 generations. Male mice were used because they were susceptible to this model of carcinogenesis. Control untreated mice in the same genetic background were age and sex-matched. Each group had 4–7 mice. All of the mice were bred in house though *bid*-deficient mice were available from the Jackson Laboratory (Bar Harbor, Maine). Mice were euthanized by cervical dislocation following anesthesia at an early stage (4–6 months) and at a later stage (10–12 months) following DEN treatment. Livers were examined and processed. All mice received humane treatment. The treatment of mice was approved by the IACUC of the University of Pittsburgh.

### Microarray studies

Mouse livers were dissected and RNA was prepared. In brief, 20 to 50 mg of liver tissues were disrupted in Buffer RLT (provided in Qiagen RNEasy kit), and homogenized. One volume of ethanol (70%) was then added to the lysate, creating conditions that promote selective binding of RNA to the RNeasy membrane. The sample was then applied to the RNeasy Mini spin column. Contaminants were washed away using RW1 wash buffer (provided in the RNEasy kit). RNA was eluted in RNase-free water. All binding, wash, and elution steps were performed by centrifugation in a micro-centrifuge.

Five micrograms of total RNA were used in the first strand cDNA synthesis with T7-Oligo (dT)24 primer (GGCCAGTGAATTGTAATACGACTCACTATAGGGAGGCGG-(dT)24) by Superscript II (GIBCO-BRL, Rockville, MD). The second strand cDNA synthesis was carried out at 16°C by adding *E coli* DNA ligase, *E coli* DNA polymerase I, and RNaseH in the reaction. This was followed by the addition of T4 DNA polymerase to blunt the ends of newly synthesized cDNA. The cDNA was purified through phenol/chloroform and ethanol precipitation. The purified cDNA was then incubated at 37°C for 4 hours in an *in vitro* transcription reaction using MEGAscript system (Ambion Inc, Austin, TX), which included biotin-conjugated CTP and UTP in the reaction to produce cRNA labeled with biotin.

For hybridization, 15 to 20 μg of cRNA were fragmented by incubating in a buffer containing 200 mM Tris-acetate, pH 8.1, 500 mM KOAc, 150 mM MgOAc at 95°C for 35 minutes. The fragmented cRNA was then hybridized with a pre-equilibrated Affymetrix mouse chip (U74Av2) at 45°C for 14–16 hours. After the hybridization cocktails were removed, the chips were then washed in a fluidic station with low-stringency buffer (6x SSPE, 0.01% Tween 20, 0.005% antifoam) for 10 cycles (2 mixes/cycle) and stringent buffer (100 mM MES, 0.1 M NaCl and 0.01% Tween 20) for 4 cycles (15 mixes/cycle), and stained with SAPE (Strepto-avidin Phycoerythrin). This was followed by incubation with biotinylated mouse anti-avidin antibody, and restained with SAPE. The chips were scanned in a HP ChipScanner (Affymetrix Inc, Santa Clara, CA) to detect hybridization signals. For quality assurance, all samples were run on Affymetrix test-3 chips to evaluate the integrity of RNA, samples with RNA 3’/5’ ratios less than 2.5 were accepted for further analysis. Hybridization data were normalized to an average target intensity of 500 per chip.

### Confirmatory assay with qRT-PCR

Total RNA from the liver was extracted using GeneTET RNA purification kit (Thermo Fisher Scientific, Grand Island, NY) according to the manufacturer’s instructions. RNA concentrations were quantified using NanoQuant Plate on an Infinite M200Pro spectrometer (Tecan, Morrisville, NC). cDNA was synthesized using M-MLV reverse transcriptase enzyme system (Life Technologies-Thermo Fisher Scientific). Gene expression was quantified using SYBR Green master mixes and a 7500 FAST Real-Time PCR System (Life Technologies-Applied Biosystems). Expression of target genes was normalized to that of β-actin. The primers used are listed in S16 Table.

### Gene expression data analysis

The CEL files generated from the microarray scanning were used as the raw data. Data have been submitted to Gene Expression Omnibus (GEO accession number: GSE80488). Data quality was examined using the RMAExpress program (v.1.0.5) (http://rmaexpress.bmbolstad.com/) [27, 28]. The density distribution along the Log2 scale was comparable among the samples, which indicated that the quality of the assay was acceptable for comparison. Microarray data from individual mouse livers within each treatment group were then averaged and the averaged values were used for the subsequently comparisons and pathway analysis. This approach might result in an improved detection of consistent changes at the expense of reduced sensitivity.

Significance of differential expression was assessed using un-paired t-test. The threshold for the significant changes was a difference larger than 25% for upregulated genes and larger than 20% for downregulated genes (fold of change >1.25 or <0.8, p<0.05). Pathway analysis was conducted using the Database for Annotation, Visualization and Integrated Discovery (DAVID) bioinformatics Resources 6.7 (https://david.ncifcrf.gov/), and Gene Set Enrichment Analysis (GSEA 2.1.)(http://software.broadinstitute.org/gsea/index.jsp), for the differentially expressed gene set and the total gene set, respectively. The pathway database is from the KEGG annotation (http://www.genome.jp/kegg/pathway.html).

In GSEA, all genes of a treatment group are ranked based on the degree of changes over the corresponding expression in the control group. Genes with a larger change in expression will be ranked closer to the top (increased) or bottom (decreased) in the list. GSEA will then rank members of a set of genes, which are associated with a given biology function (pathway), among all genes with altered expression. Gene sets with more members ranked at the top or bottom in the list are considered to be enriched or over-represented among all sets. The enrichment score (ES) is calculated for each gene set for the degree of enrichment among all sets. The statistical significance of ES is calculated (nominal *p* value). In addition, ES was normalized for the size of the gene set, which yielded the normalized ES (NES). The probability that NES represents a false positive finding was estimated and represented by the false discovery rate (FDR) and familywise error rate (FWER).

## Results and Discussion

### There were more genes showing expression changes in wild type livers than in *bid*-deficient livers

**Fig 1:**
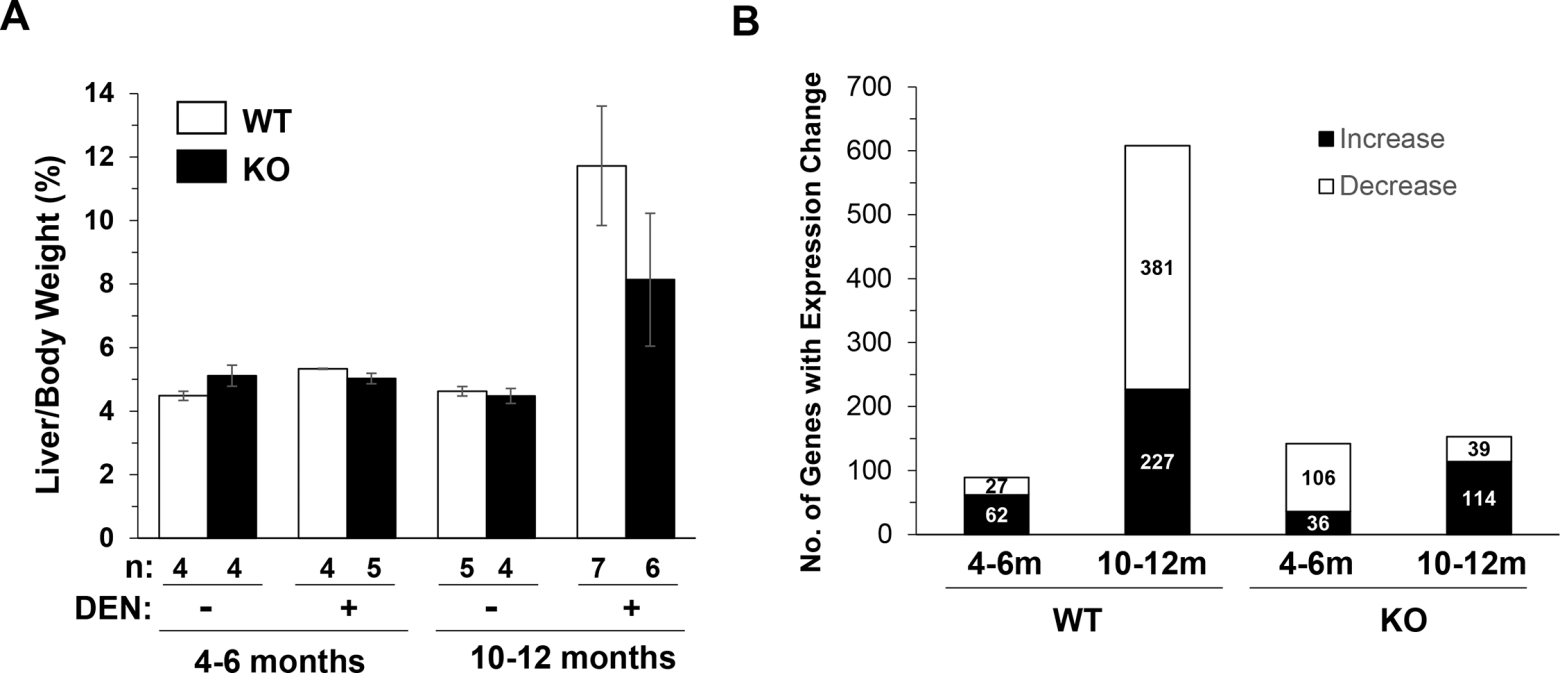
Bid-deficient livers have a lower tumor burden and fewer gene expression alterations following long-term DEN treatment. Wild type (WT) and *bid*-deficient (KO) mice were treated with or without DEN for 4–6 or 10–12 months. (**A**) The percentage of liver weight in the body weight was calculated as a parameter for tumor burden. The numbers (n) of mice in each group were shown. (B). The livers were subjected to microarray analysis. The data in each group were averaged and the numbers of genes with increased and decreased expression in the DEN-treated livers in comparison with the normal age-matched samples were shown in solid and open columns, respectively.

Wild type and *bid*-deficient mice were given DEN at the neonatal stage demonstrated significantly different tumor development process [12, 19]. We decided to examine the gene expression prolife in mice treated with DEN at two different time points based on previous observations [12]. The first time point was 4 to 6 months. This was the period when microscopic tumor foci were formed. There were no grossly observable tumors at this time. The second time point was 10 to 12 month. Grossly observable tumors could be well defined at this period. Previous studies had well demonstrated that *bid*-deficient mice developed less tumors in number and in size, which led to a relatively smaller liver/body weight ratio comparing to the wild type mice [12, 19]. The ratio of the liver/body weight was thus significantly increased in wild-type mice at the later stage (Fig 1A). The size of the liver could be used as an assessment of the tumor burden [12]. Consistently, the liver/body weight ratio was not as significantly increased in *bid*-deficient mice (Fig 1) as previously observed [12, 19].

**Fig 2:**
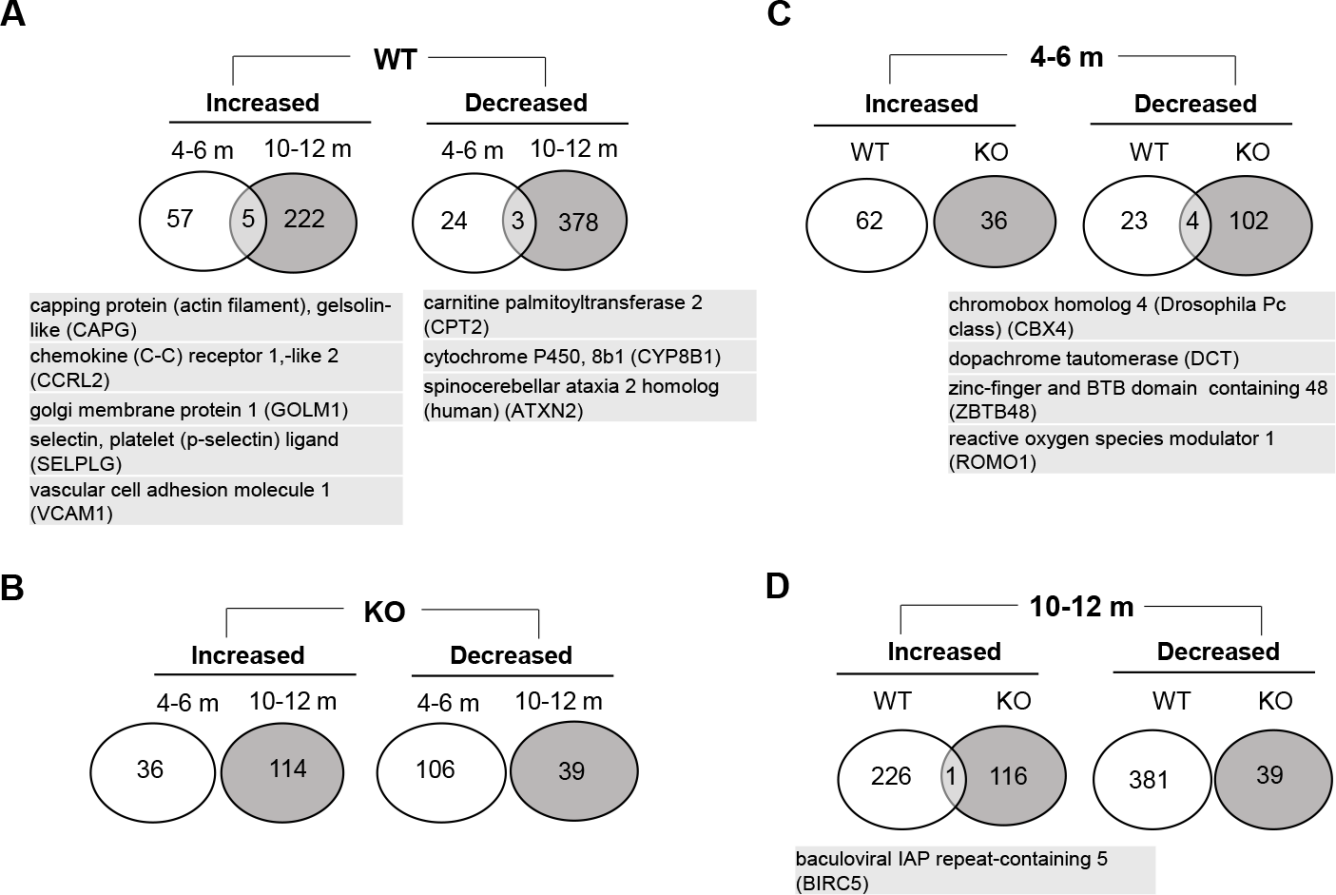
There are minimal overlaps in altered genes between DEN-treated wild type and *bid*-deficient mice, and between mice treated with DEN for different times. (A-B). Genes with altered expression was compared between the different time points (4–6 month vs 10–12 month) for the DEN-treated wild type (A) and *bid*-deficient (B) livers. Overlapped genes between the time points were shown in the diagram and listed. (C-D). Genes with altered expression were compared between DEN-treated wild type (WT) and *bid*-deficient (KO) livers at the time point of 4–6 month (C) or 10–12 month (D). Overlapped genes between the two groups of mice were shown in the diagram and listed. All genes are listed in S1–S8 Tables. Size of the diagram and overlapped region are not proportional to the number of genes.

The expression of genes in the livers of DEN-treated mice was compared to that in the livers of age- and sex-matched non-treated control mice with the same genetic background (S1–S8 Tables). The comparison was not made between tumors and adjacent non-tumor tissues because the tumors developed in a diffusive manner and in cases where tumors could be visually separated it was suspected that the adjacent tissues were not microscopically tumor-free. In wild type mice treated with DEN for 4–6 months we found that expression of 62 genes was increased while 27 was decreased compared to the non-treated control mice (Fold of change >1.25, *p* <0.05) (Fig 1B). There was a significant increase in the number of genes whose expression was altered in wild type mice treated with DEN for 10–12 months (227 increase and 381 decrease)(Fig 1B). This was likely due to the progression of DEN-induced carcinogenesis through the liver and the formation of grossly recognizable tumors, causing a more dramatic deviation of the gene expression than that in the normal liver. Notably, there were few overlaps in these significantly altered genes between the 4–6 month and the 10–12 month groups. Only 5 and 3 genes in the upregulated and downregulated lists, respectively, were shared by the early and the later stage groups in the wild type mice (Fig 2A). Few genes remained altered in expression from the early to the later stage. This result suggested that different genetic programs were involved at the microscopic tumor stage and at the gross tumor stage.

To examine the impact of the deletion of Bid on DEN-induced carcinogenesis, we looked at the gene expression profile in *bid*-deficient mice. A total of 142 genes (36 increased and 106 decreased) had altered expression to a significant level (Fold of change >1.25, *p* <0.05) at the early stage (Fig 1B). However, the change of gene expression in the 10–12 month group was only slightly enhanced, with a total of 153 genes displaying alterations (114 increased and 39 decreased)(Fig 1B). There were no overlaps for these significantly changed genes between the early and the later groups (Fig 2B), similar to what was observed in the wild type mice.

What was remarkable was that the number of genes (153) with significantly altered expression in *bid*-deficient livers was much less than that (608 genes) in the wild type mice at the later stage (Fig 1B). The results were consistent with the finding that there were much less grossly recognizable tumors in *bid*-deficient livers at this time [12, 19](Fig 1A), and indicated that the DEN-induced carcinogenesis was affected by Bid-mediated events. This difference was not only in the quantity, but also in the profile. There was little similarity in genes whose expression changed following DEN treatment between the wild type and *bid*-deficient livers. Only 4 genes were shared in the 4–6 month groups and one gene was shared in the 10–12 month groups (Fig 2C and 2D). Thus in the absence of Bid the carcinogen-driven changes in the gene expression came out with a different profile.

**Fig 3:**
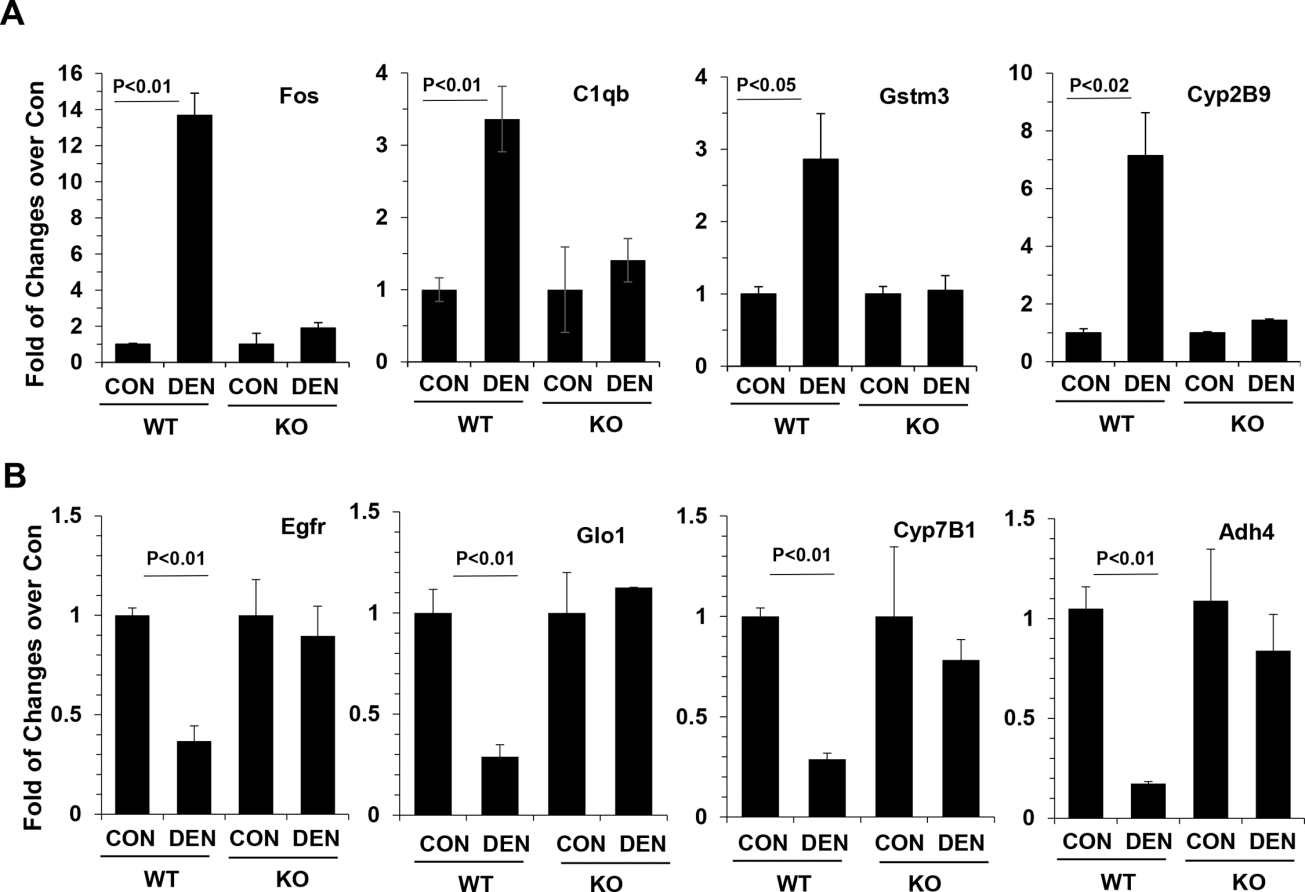
Confirmation of gene expression by qRT-PCR. Total RNA was extracted from the livers of wild type and *bid*-deficient mice treated with or without DEN for 10–12 months. qRT-PCR was performed with primers for the indicated genes that were elevated (A), or suppressed (B) in expression in wild type (WT) mice treated with DEN for 10–12 month. Expressions in *bid*-deficient mice (KO) were also assessed but did not show significant changes for these genes. The fold of changes over the non-treated controls was calculated. Mean+/-SEM (n = 3–4 per group).

We confirmed the differential expression of a selected number of genes using quantitative RT-PCR (Fig 3). For example, the expression of Fos, C1qb, Gstm3 and Cyp2B9 was higher in livers of DEN-treated wild type mice than in livers of non-DEN treated control (S3 Table) while these genes were not found to be significantly differently expressed in DEN-treated *bid*-deficient livers at the same stage (S7 Table). RT-PCR assay demonstrated the same expression pattern of genes among the four groups of mice (Fig 3A). Similarly, the expression of Egfr, Glo1, Cyp7B1 and Adh4 was down-regulated in DEN-treated wild type livers (S4 Table), but not significantly altered in DEN-treated *bid*-deficient livers (S8 Table) at the 10–12 month stage. These findings were confirmed by the qRT-PCR assay (Fig 3B).

### More functional pathways were affected in wild type livers than in *bid*-deficient livers following DEN treatment

We then examined the expression profiles of these groups (S1–S8 Tables). The degree to which the expression altered was different for different genes. Here we defined the threshold for the significant changes to be a difference larger than 25% for upregulated genes and larger than 20% for downregulated genes. The degree to which the gene expression altered was slightly but significantly higher in wild type livers than in the *bid*-deficient livers in the later stage. The differences were, however, statistically significant as the large amount of genes involved for this comparisons. Thus the mean fold of change for the increased expression in the wild type livers was 1.67±0.93 (median = 1.42, range: 1.25–10.07) vs 1.45±0.27 (median: 1.38, range: 1.25–3.6) in the *bid*-deficient livers. T test indicated a *p* value of 0.014. On the other hand, the mean fold of change for the decreased expression in the wild type livers was 0.64±0.12 (median: 0.66, range: 0.8–0.076) vs 0.74±0.06 (median: 0.76, range: 0.8–0.46) in the *bid*-deficient livers. T test indicated a *p* value of <0.001.

Thus for the upregulated genes, the largest fold of change was 1.53 (CISH, cytokine inducible SH2-containing protein) for the early stage wild type liver (S1 Table), 10.08 (LCN2, lipocalin 2) for the later stage wild type liver (S3 Table), 3.22 (CYP2B9, cytochrome p450, 2b9) for the early stage *bid*-deficient liver (S5 Table), and 3.60 (H19, imprinted maternally expressed transcript) for the later stage *bid*-deficient livers (S7 Table), respectively. Similarly, for the downregulated genes, the largest fold of change was 0.58 (AHR, aryl-hydrocarbon receptor) for the early stage wild type liver (S2 Table), 0.076 (HSD3B5, hydroxysteroid dehydrogenase-5) for the later stage wild type liver (S4 Table), 0.46 (ERDR1, erythroid differentiation regulator 1) for the early stage *bid*-deficient liver (S6 Table), and 0.46 (ALAS1, aminolevulinic acid synthase 1) for the later stage *bid*-deficient livers (S8 Table), respectively. These genes were quite different and did not seem to be correlated with the genotypes or the time as noted in Fig 2.

Many of the upregulated genes in the wild type livers at the later stage were related to immune response and/or inflammation response (S3 Table), whereas many of the down-regulated genes were related to hepatic metabolic functions (S4 Table). Alterations of these genes were not as obvious in the *bid*-deficient livers, suggesting that expression changes of these genes could contribute to the tumorigenesis that was delayed in the absence of Bid.

In order to better understand the significance of the altered gene expression, we subjected genes with differential expression (S1–S8 Tables) to pathway analysis using the DAVID bioinformatics tool. This analysis grouped genes based on functional similarity and allowed us to find whether genes representing a certain biological function or KEGG pathways were particularly enriched in the differentially expressed gene list. A single gene could be grouped in more than one functional cluster. We also subjected the entire gene expression sets to GSEA without considering the degree of change for each individual gene. GSEA has the advantage of looking at the pathway change in a global way without limiting to genes with large alterations in expression (>20%)[29]. These genes are often limited in number, but many more genes could have smaller changes. If many of these so-called minor genes are enriched in a particular pathway, the impact can still be significant for the signaling or the flux of that pathway. This impact could be bigger than what might be caused by a drastic expression change of a single gene. In both analyses, we used the KEGG mouse pathway database to assess which pathways are affected.

**Fig 4:**
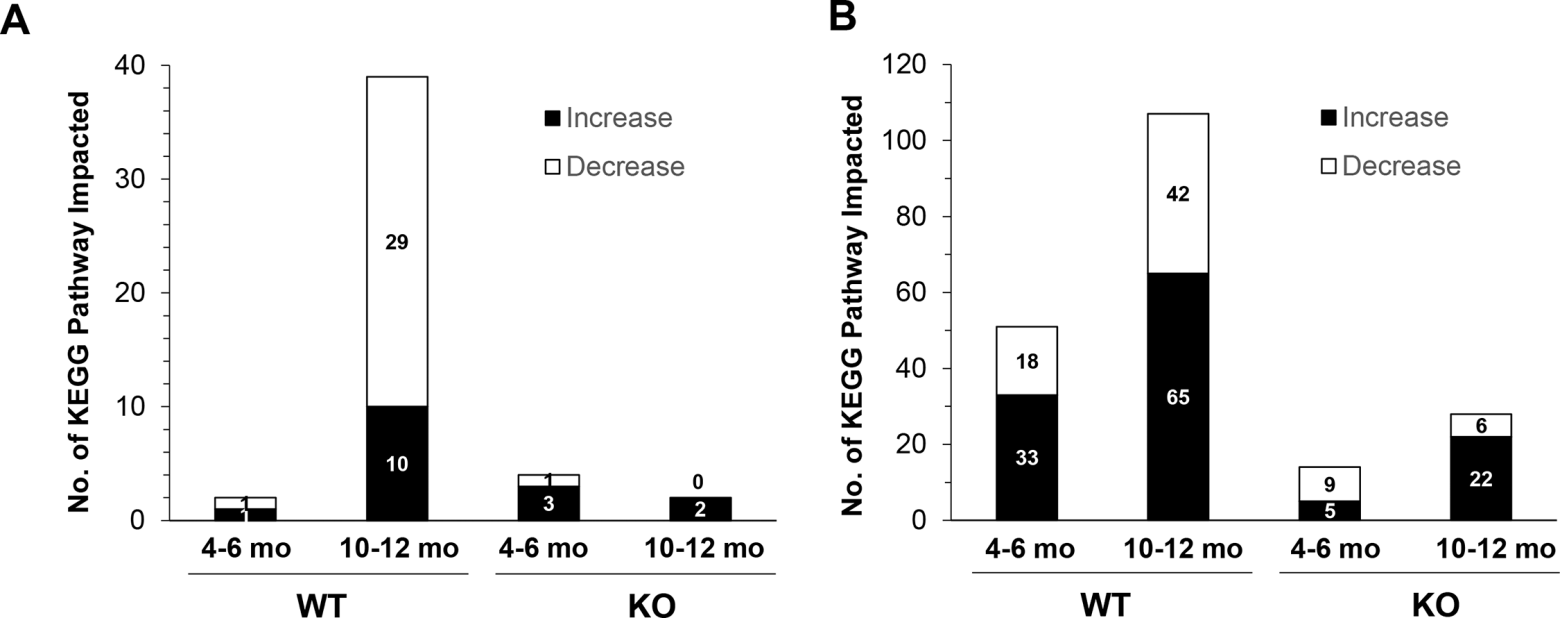
Significantly more biological pathways are altered in wild type mice than in *bid*-deficient mice following DEN treatment. (A). Genes showing significantly altered expressions in DEN-treated wild type (WT) and *bid*-deficient (KO) liver were subjected to DAVID analysis coupled with KEGG Pathway analysis. (B). All genes in DEN-treated wild type (WT) and *bid*-deficient (KO) liver were subjected to GSEA coupled with KEGG Pathway analysis. The number of upregulated (increase) or downregulated (decrease) pathways in each group was plotted and indicated. Wild type mice treated with DEN for 10–12 months had the largest number of pathways affected.

Analysis of the differentially expressed gene list using DAVID indicated that there were only limited pathways that were enriched (Fig 4A, S9 Table). As anticipated, wild type mice treated with DEN for 10–12 months had more pathways affected: 10 KEGG pathways were upregulated, whereas 29 KEGG pathways were downregulated. Less than 10 pathways were affected in all the other three groups. Much fewer pathways were affected in *bid*-deficient mice at the later stage of 10–12 months, although a similar number of pathways were affected at 4–6 months in both types of mice. Selected genes identified in these pathways were listed in S10 and S11 Tables. These genes had altered expressions more than 25% (for the upregulated ones), or 20% (for the down-regulated ones), and thus could be also found in the extended lists of genes with significantly altered expression (S3 and S4 Tables).

On the other hands, GSEA using the total gene set yielded much richer information regarding which pathways were affected (Fig 4B, S12–S15 Tables). Thus, in wild type mice treated with DEN for 10–12 months, the numbers of upregulated and downregulated pathways were 65 and 42, respectively. A fewer number of pathways were affected in wild type mice treated with DEN for 4–6 months (33 upregulated and 18 downregulated). Similar to DAVID analysis, GSEA revealed that DEN caused a much less number of pathways that were altered in mice deficient in Bid. A very limited set of KEGG pathways were affected either at the early stage (5 upregulated and 9 down-regulated) or at the later stage (22 upregulated and 6 down-regulated).

Overall the number of pathways affected (Fig 4) was consistent with the number of genes with significant changes (Fig 1B), and the trends remained similar between the results obtained with DAVID analysis and those with GSEA. However, GSEA revealed that more pathways were actually changed following DEN treatment.

### There was a more significant downregulation of intermediate metabolism pathways in wild type livers than in *bid*-deficient livers

**Fig 5:**
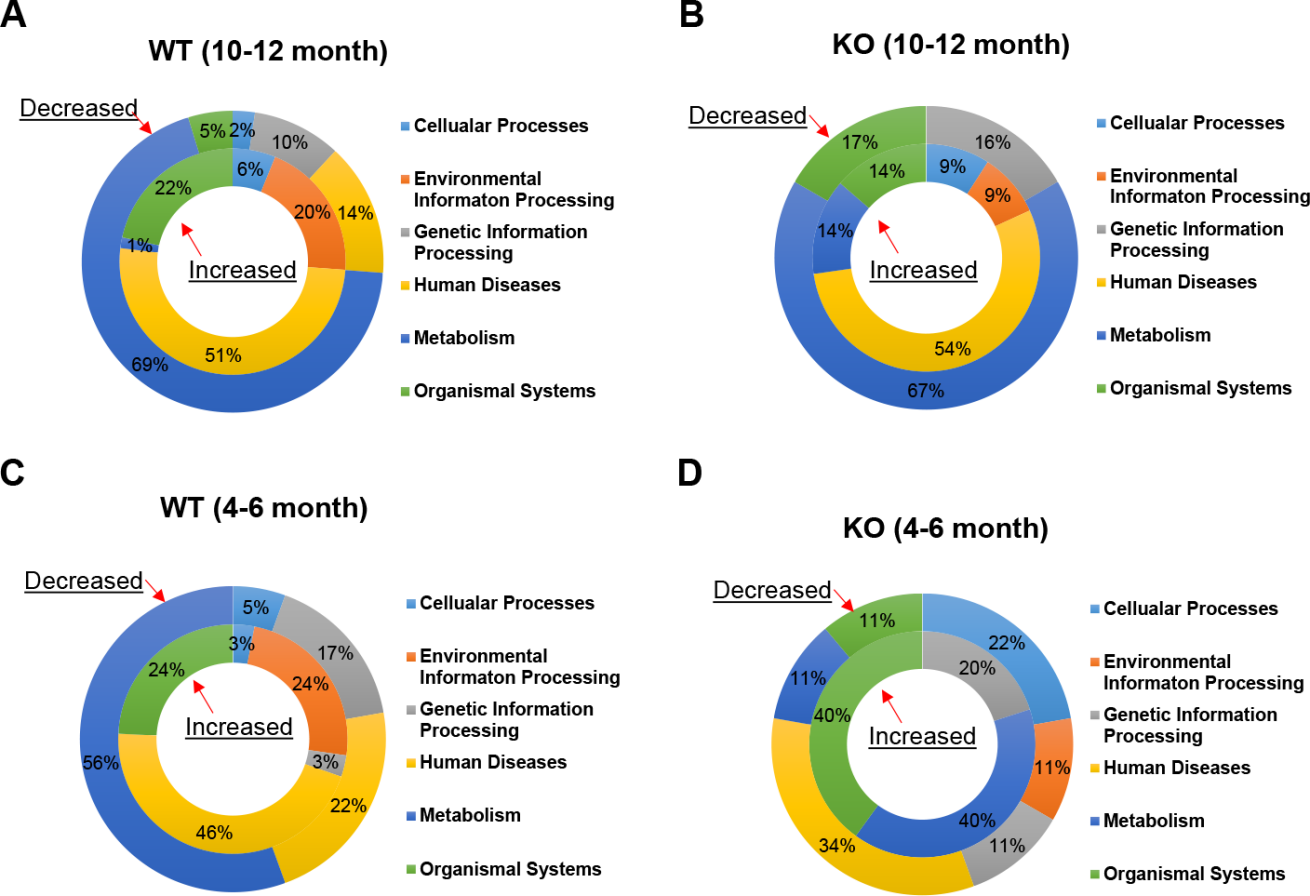
DEN treatment affects different functional pathways to different degrees in wild type vs *bid*-deficient mice. GSEA-identified biological pathways were grouped into six functional groups per KEGG pathway analysis for wild type (WT) and *bid*-deficient (KO) mice given DEN for 4–6 months or 10–12 months. Upregulated (increased, inner circle) and downregulated (decreased, outer circle) functional groups were displayed based on the percentage distribution. Pathways related to metabolism and diseases are most frequently affected. Functional groups that were not detected were not shown in the diagram.

We then analyzed the nature of the affected pathways that were determined through GSEA. The KEGG pathways can be grouped under 6 major functional clusters. The most affected function was related to the down-regulation of metabolism in both wild type and *bid*-deficient mice at the later stage of DEN treatment (Fig 5A and 5B), which accounted for 69% and 67% of all pathways downregulated, respectively. Notably, metabolic downregulation was also significant (56%) in the early stage of DEN treatment in wild type mice, but not in *bid*-deficient mice (11%) (Fig 5C and 5D).

**Fig 6:**
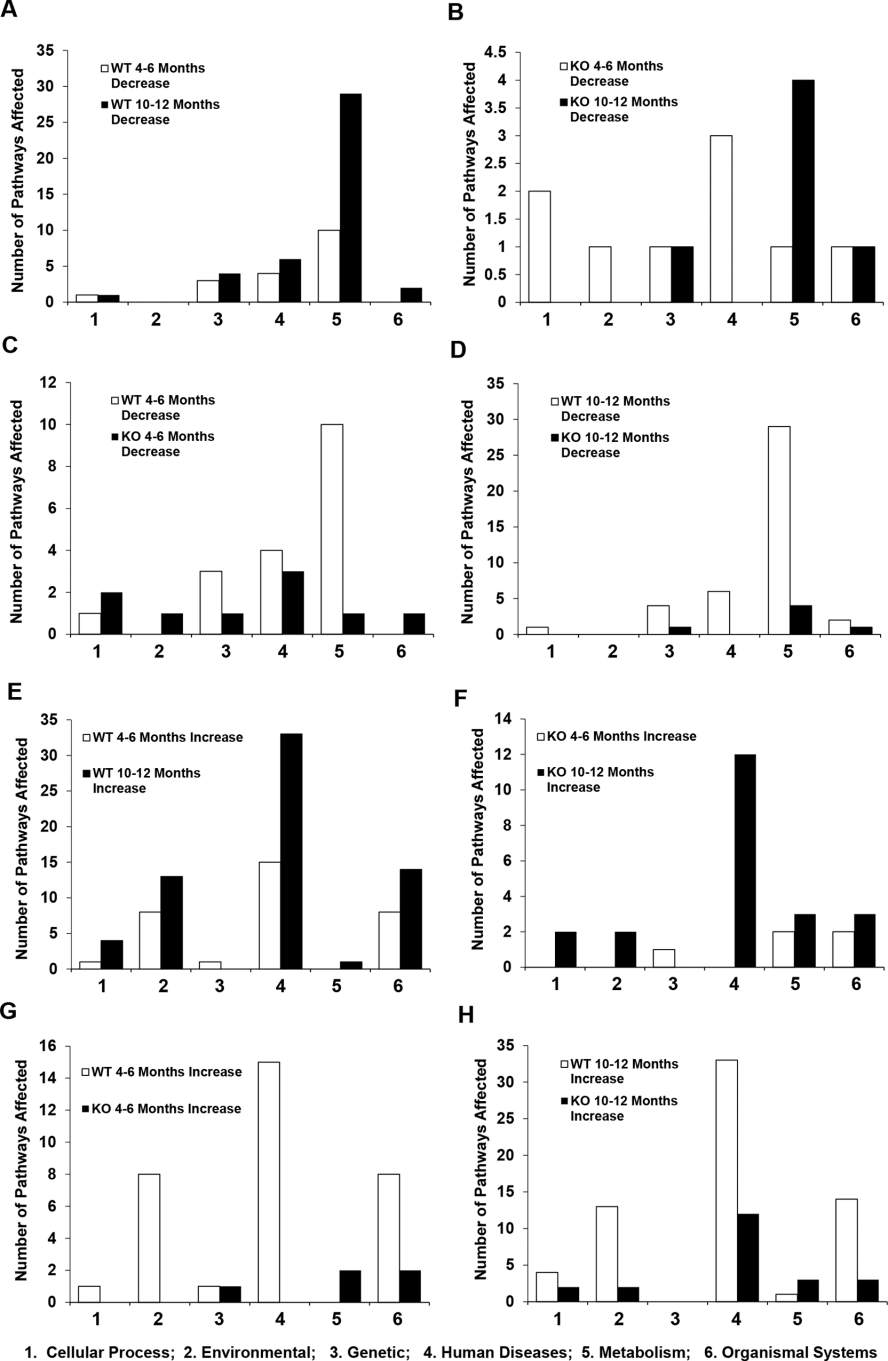
Biological pathways related to metabolism and diseases are most affected by DEN-induced carcinogenesis. GSEA-identified biological pathways were grouped into six functional groups per KEGG pathway analysis for wild type (WT) and *bid*-deficient (KO) mice given DEN for 4–6 months or 10–12 months. The number of downregulated pathways (A-D) and the upregulated pathways (E-H) were compared among different mouse groups. The most commonly downregulated biological pathway cluster was related to metabolism (cluster 5, A-D), whereas the most commonly upregulated biology pathway cluster was related to human diseases (cluster 4, E-H). For these two clusters, wild type mice had more pathways affected than *bid*-deficient mice; and later stage (10–12 months) group had more pathways affected than the early stage (4–6 months) group. Pathway functional groups: 1: Cellular Process; 2: Environmental Information Processing; 3: Genetic Information Processing; 4: Human Diseases; 5: Metabolism; 6: Organismal Systems.

The actual number of metabolic pathways downregulated was more significant in wild type mice than in *bid*-deficient mice, 29 vs 4 in the 10-12-month group (Fig 6A and 6B) and 10 vs 1 in the 4–6 month group(Fig 6C and 6D). This difference indicated a more rapid and significant change in the wild type livers due to DEN-induced carcinogenesis, whereas *bid*-deficient liver were resistant to these changes. In the 29 downregulated metabolic pathways in the later stage wild type mice, about one third (9) were related to amino acid metabolism, one fourth (7) were related to lipid and steroids metabolism and another one-fifth (6) were about carbohydrate metabolism (S13 Table). Downregulation of some of the metabolism pathways was also noted in the early stage wild type group (S12 Table), and in the *bid*-deficient group (S14 and S15 Tables) with diminished scales. However, amino acids metabolism seemed to be particularly downregulated in the later stage wild type livers. Considering that these metabolisms represent key functions of hepatocytes, downregulation of these pathways suggested a significant level of de-differentiation of hepatocytes under DEN-induced carcinogenesis, which occurred early in the process, and became quite significant later. The lack of equally notable change in the *bid*-deficient mice was consistent with a delayed progression of the poorly differentiated tumors in these mice (Fig 1A)[12].

The downregulation of metabolism pathways was also evident from DAVID analysis on the differentially expressed genes (S9 Table). In the later stage wild type mice, 23 of the 29 downregulated pathways were in the Metabolism cluster. Eight (8) of these 23 pathways were for amino acids metabolism. Thus the pathway distribution of the genes with significantly downregulated expression (S9 Table) was similar to the pathway distribution of many other genes that had smaller changes (S12–S15 Tables), but the latter represented a larger scope of the change.

### There was a more significant upregulation of pathways related to cell growth and immune response in wild type than in *bid*-deficient livers

The next mostly affected functional cluster observed via GSEA was related to Human Diseases. More of these pathways were upregulated than downregulated in all DEN-treated groups except the early *bid*-deficient group (Fig 5). More such pathways were enriched in the later stage than in the early stage (51% vs 46% in wild type, and 54% vs 0% in knockout mice) (Fig 5, Fig 6E and 6F), and in wild type mice than in *bid*-deficient mice in the early stage (46% vs 0%) (Fig 5, Fig 6G and 6H).

These pathways represented very diverse functions that were implicated in the pathogenesis of a variety of human diseases. However, in the 33 upregulated disease-related pathways in the 10–12 month wild type mice (S13 Table), one half (16) were related to infectious diseases, one-fifth (6) were about immune diseases and another one fourth (8) were related to cancer. Interestingly, in the 15 upregulated disease-related pathways for wild type mice at early stage (S12 Table), again one half (8) of the pathways were related to infectious diseases, one fifth (3) were related to immune diseases, and 2 were related to cancer. Although much less prominent in the *bid*-deficient mice, upregulated immune disease/infectious disease/cancer-related pathways were still more enriched in the later stage (S15 Table) than in the early stage of carcinogenesis (S14 Table). For the 12 pathways in the later stage group (S15 Table), those related to immune response and infectious diseases still accounted for about one half (5), and those related to cancer accounted for about one fourth (3). Thus the two upregulated gene profiles in the cluster of Human Diseases in the DEN-induced carcinogenesis were related to “cancer” and “immune diseases/infectious diseases”.

It was not surprising that genes and pathways related to cancer development were upregulated in DEN-treated livers. These pathways were selected based on what are known in the development of cancer in general, and in lung, colon, pancreatic cancer and basal cell carcinoma in particular (S12–S15 Tables). Correlating with these findings, several pathways related to cell growth and cell survival in the cluster of “Environmental Information Processing: Signal Transduction” were upregulated in DEN-treated livers. These included cAMP pathway, MAP kinase pathway, JAK-STAT pathway and Wnt pathway. Also upregulated were pathways related to cell adhesion and extracellular matrix (ECM)-receptor interaction (“Environmental Information Processing: Signaling Molecules and Interaction”), and pathways related to cell cycle, cell motility and focal adhesion (“Cellular Process”). Changes in all these pathways were clearly associated with the tumorigenesis and the promotion of cancer development. The significant differences between the wild type and *bid*-deficient mice in terms of the number of the growth control-related pathways and the size of the pathways (i.e., the number of genes involved in each pathway) were consistent with a more aggressive development of HCC in the wild type mice.

A somewhat unexpected but certainly reasonable finding was the even more significant involvement of pathways that were related to immune responses (S12–S15 Tables). The pathways of Immune Disease and of Infectious Diseases were well connected and intertwined. The Infectious Diseases pathways were mainly about immune response to intracellular pathogens (virus, parasites and bacteria) and thus were overlapped with the pathways classified under the cluster Immune Diseases. Furthermore, Genes involved in these pathways were overlapped with those in the pathways classified under “Organismal System: immune response”, many of which were also upregulated. Thus these upregulated pathways could together represent a significant activation of the innate and adaptive immune response, which can also be associated with an enhanced inflammation process. From the latter point of view, it was also evident that there was an upregulation of the NF-κB pathway, TNF pathway and cytokine-cytokine receptor interaction pathways, which were clustered under the “Environmental Information Processing: Signal Transduction” and “Environmental Information Processing: Signaling Molecule and Interaction” (S12 and S13 Tables). The Environmental Information Processing was the third major group with significant upregulation (Fig 5E and 5F).

It is thus notable that the upregulation of the cancer-related pathways and immune response/inflammation-related pathways was evident not only in one functional cluster (Human Diseases), but also in several other function clusters (“Cellular Processes, Environmental Information Processing”, and “Organismal System”). Overall, in the 65 upregulated pathways in later stage wild type mouse group (S13 Table), 42 pathways (64.6%) were related to the above-mentioned functions. These changes thus suggested the significant participation of the immune system and inflammation in the DEN-induced carcinogenesis. The importance of these pathways has been well discussed in cancer development [25, 26, 30]. More significant upregulations were seen in the later stage than in the early stage mouse group (Fig 6E and 6F). Equally notable was that these changes were much diminished in the absence of Bid (Fig 6G and 6H), suggesting that the Bid played a critical role in initiating and/or promoting many of these changes.

Upregulation of pathways in immune response/inflammation was also evident in DAVID analysis of those highly differentially expressed genes (S9 and S10 Tables). In 10–12 month DEN-treated wild type mice, 6 of the 10 upregulated pathways were in the “Organismal Systems; Immune System” or “Human Diseases; Immune diseases” cluster. One pathway under the cluster of “Human Diseases; Neurodegenerative diseases, Prion diseases”, was overlapped with the above pathways in terms of genes involved. Overall, these pathways involved innate immune response (complement cascade, Fc gamma receptor-mediated neutrophil and macrophage activation, leukocyte transmigration), inflammation (Toll-like receptor signaling) and cytokine activation of the hematopoietic system. Again the pathway distribution of the genes with significantly upregulated expression (S9 Table) was similar to the pathway distribution of many other genes that had smaller changes (S12–S15 Tables), but the latter represented a larger scope of the change.

## Conclusions

The microarray analysis indicated that alterations in gene expression occurred earlier in time and broader in scope in wild type mice than in *bid*-deficient mice following DEN treatment. This finding is consistent with the delayed and diminished carcinogenesis in the *bid*-deficient mice [12, 19]. For the liver, the most important metabolic organ in the body, the down-regulation of many genes involved in metabolism suggested a loss of one of its major functions following DEN treatment. This may suggest a process of dedifferentiation, commonly seen in cancers, which can also be characterized by the re-expression of fetal proteins. A less dedifferentiated status of the *bid*-deficient livers implied that these livers retained the normal function for a longer time under the carcinogen influence, which is consistent with a delayed progression of cancer in these livers.

**Fig 7:**
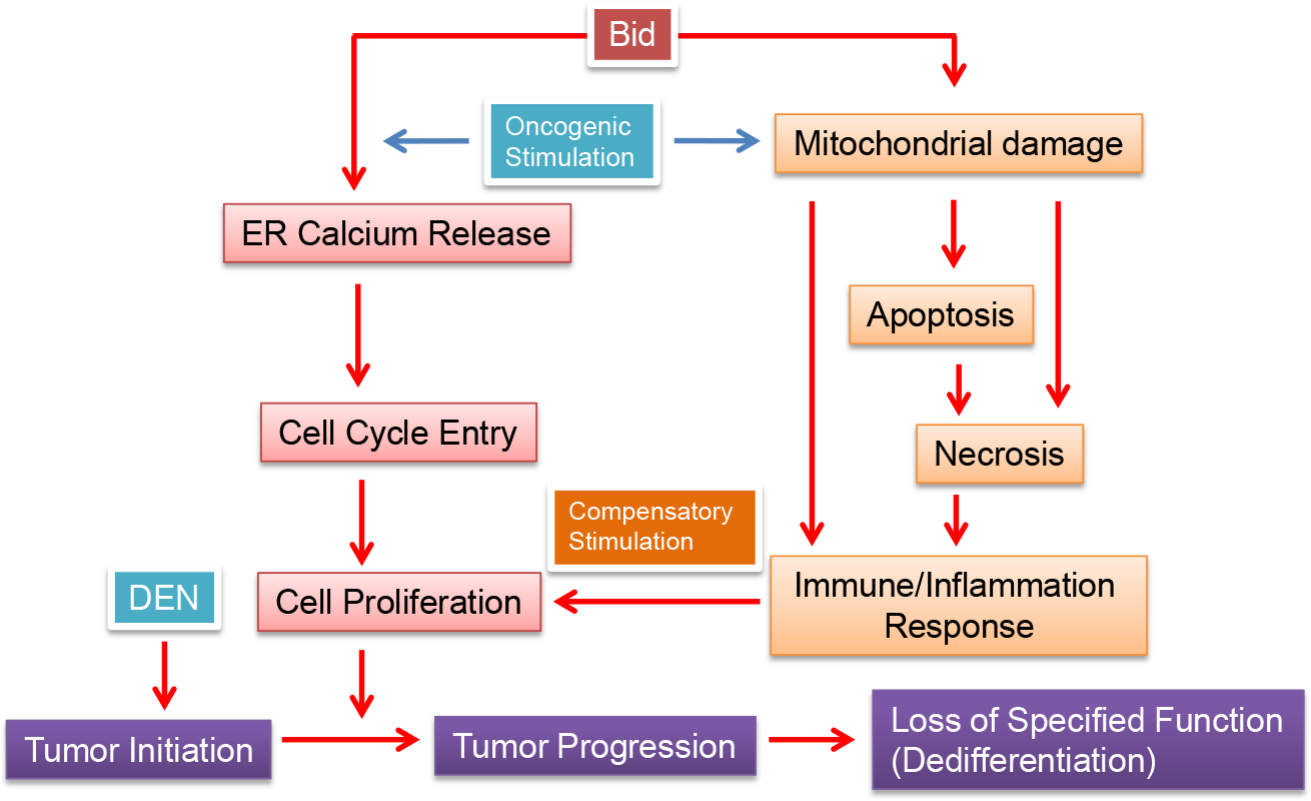
Bid promotes DEN-induced hepatic carcinogenesis. The BH3-only Bcl-2 family molecule Bid can promote DEN-induced hepatic carcinogenesis by enhancing cell proliferation. This function may be mediated by at least two mechanisms. One is internally stimulated by oncogenic transformation, which can be regulated by Bid’s action on calcium release from ER, which is required for cell cycle entrance. The other is externally stimulated by cell death, which could be also affected by Bid-mediated pathway. Although the mechanism is not clear DEN-induced liver injury could be affected by Bid. There could be necrotic cell death, either secondary to apoptosis, or primarily due to DEN-induced damage. Immune response and/or inflammation responses are then activated. Bid can participate in inflammasome activation caused by mitochondrial damage [10]. These factors can stimulate proliferation of neighboring cells that are mutated but survived. This process is known as compensatory proliferation. Note that Bid may at the same time regulate the proliferation of these survived neighboring cells through the intrinsic mechanism on ER-calcium release. Tumor cells may become dedifferentiated and lose some of the key hepatic metabolic functions, such as that for amino acids. Gene expression data in this study indicate a significant upregulation of genes involved in cancer growth and in immune/inflammation response, and a significant downregulation of genes involved in metabolism. All of these changes are diminished in the absence of Bid, suggesting the role of Bid in regulating these events.

The driving force of tumor development is commonly related to deregulated growth. We found that growth-orientated signaling pathways were upregulated in wild type mice more than in the *bid*-deficient mice both in terms of the pathways involved and the number of the genes involved (S12–S15 Tables). This is correlated with the more aggressive tumor growth in the wild type mice. It is possible that the intrinsic ability of Bid to promote cell proliferation [12, 13] contributes to the activation of the growth-orientated pathways. Indirectly, the ability of Bid to promote cell death can also contribute to the growth of tumors because cell death can serve as a compensatory stimulus. The deletion of Bid would cause less cell death and liver injury [12, 19]. Similar observations were made in mice deficient in PUMA, another pro-death BH3-only Bcl-2 family molecule, following DEN treatment [24]. Less cell death could potentially lead to less compensatory stimulation, which, nevertheless, would still depend on the intrinsic cell growth mechanism to promote cell proliferation, and may still be subjected to Bid-regulated ER calcium events (Fig 7).

The mechanism of compensatory proliferation is not fully understood. The inflammatory cytokines may promote cell survival and compensatory proliferation [25, 26, 30]. Although Bid and PUMA mediate apoptosis, not necrosis, apoptosis can be followed by secondary necrosis, which may trigger a secondary immune and inflammatory response. There was some biochemical evidence to support this hypothesis [19, 24]. The present study provided evidence at the gene expression level, supporting the involvement of immune/inflammatory response in DEN-induced carcinogenesis. The external compensatory stimulation and the internal oncogenic stimulation can act on the same cell proliferation machinery, which would be subjected to the direct effect of Bid through the control on ER calcium release [12, 13] (Fig 7). The external compensatory stimulation on cell proliferation could thus have a considerable promoting effect on how DEN-induced tumors develop and expand. It is important to keep in mind that Bid, as a multi-functional molecule, could also negatively regulate tumorigenesis in different contexts of tissues and stimulations via activities such as maintaining S phase checkpoint [14–18]. Which of these functions becomes a dominant factor in a given context is an interesting and meaningful question for future studies.

## Supporting Information

S1 Table Up-regulated genes in livers of wild type mice treated with DEN for 4–6 months. (PDF)

S2 Table Down-regulated genes in livers of wild type mice treated with DEN for 4–6 months. (PDF)

S3 Table Up-regulated genes in livers of wild type mice treated with DEN for 10–12 months. (PDF)

S4 Table Down-regulated genes in livers of wild type mice treated with DEN for 10–12 months. (PDF)

S5 Table Up-regulated genes in livers of Bid-deficient mice treated with DEN for 4–6 months. (PDF)

S6 Table Down-regulated genes in livers of Bid-deficient mice treated with DEN for 4–6 months. (PDF)

S7 Table Up-regulated genes in livers of bid-deficient mice treated with DEN for 10–12 months. (PDF)

S8 Table Down-regulated genes in livers of Bid-deficient pe mice treated with DEN for 10–12 months. (PDF)

S9 Table DAVID pathway analysis of differentially expressed genes in DEN-treated mouse livers. (PDF)

S10 Table Genes that are involved in immune response and/or inflammation, and that are upregulated in WT mice treated with DEN for 10–12 Months. (PDF)

S11 Table Genes that are involved in amino acid metabolism, and that are down-regulated in WT mice treated with DEN for 10–12 Months. (PDF)

S12 Table Pathway analysis of the gene expression profile in wild type mice treated with DEN for 4–6 months. (PDF)

S13 Table Pathway analysis of the gene expression profile in wild type mice treated with DEN for 10–12 months. (PDF)

S14 Table Pathway analysis of the gene expression profile in bid-deficient mice treated with DEN for 4–6 months. (PDF)

S15 Table Pathway analysis of the gene expression profile in bid-deficient mice treated with DEN for 10–12 months. (PDF)

S16 Table List of qRT-PCR primers. (PDF)
