# Supplementary material for: Gene Expression Analysis Indicates Divergent Mechanisms in DEN-Induced Carcinogenesis in Wild Type and Bid-Deficient Livers
Source: PLoS One. 2016 May 19;11(5):e0155211. doi: 10.1371/journal.pone.0155211 (PMC4873180; doi:10.1371/journal.pone.0155211)
Supplement: S1 Table — (PDF) [file pone.0155211.s001.pdf]

S1 Table. Up-regualted genes in livers of wild type mice treated with DEN for 4-6 months

Genes Symbol	Gene Name	Probes	FC	p value	Function
C8G	complement component 8, gamma subunit	161837_r_at	1.2700	0.0344	Complement_ innate immunity
CALCRL	calcitonin receptor-like	97795_at	1.3977	0.0326	Neuroactive_ligand_receptor_interaction, Vascular_smooth_muscle_contraction
CAPG	capping protein (actin filament), gelsolin-like	160106_at	1.2644	0.0107	Actin binding
CAR12	carbonic anyhydrase 12	103905_at	1.2797	0.0306	Nitrogen_metabolism
CCRL2	chemokine (C-C) receptor 1,-like 2	93617_at	1.2961	0.0407	Signaling by GPCR and Akt Signaling
CD151	CD151 antigen	161823_r_at	1.3519	0.0030	Degradation of the extracellular matrix and Cell junction organization
CISH	cytokine inducible SH2-containing protein	100022_at	1.5277	0.0429	TGF-Beta Pathway and Kit Receptor Signaling Pathway
CLCN2	chloride channel 2	92385_at	1.2980	0.0125	Mineral_absorption
CLPX	caseinolytic protease X (E.coli)	94961_at	1.3211	0.0437	ATPase activity and peptidase activator activity
CMIP	c-Maf inducing protein	162239_at	1.2876	0.0137	Regulation of Wnt-beta catenin signaling
CRYAB	crystallin, alpha B	162308_f_at	1.2659	0.0023	Protein_processing_in_endoplasmic_reticulum
DMRT1	doublesex and mab-3 related transcription factor 1	100374_at	1.2580	0.0161	Transcription Regulation
DSG2	desmoglein 2	98319_at	1.3707	0.0288	Arrhythmogenic_right_ventricular_cardiomyopathy_(ARVC)
DSPP	dentin sialophosphoprotein	94721_at	1.2664	0.0020	ERK Signaling and Degradation of the extracellular matrix
EGR2	early growth response 2	102661_at	1.2984	0.0130	Viral_carcinogenesis, infection
EIF4B	eukaryotic translation initiation factor 4B	100557_g_at	1.3262	0.0135	mTOR_signaling_pathway, PI3K_Akt_signaling_pathway
FHL1	four and a half LIM domains 1	97500_g_at	1.3124	0.0474	Notch signaling pathway
GATA6	GATA binding protein 6	104698_at	1.2897	0.0483	Heart Development and Hemostasis
GOLM1	golgi membrane protein 1	95593_at	1.2658	0.0312	Nucleus organization, regulation of lipid metabolic process
H2-Eb2	Histocompatibility 2, class II antigen E beta2	97048_at	1.2736	0.0364	Equivalent to human MHC Class II, HLA-DRB5
HCN1	hyperpolarization-activated, cyclic nucleotide-gated K	99902_at	1.2632	0.0359	Transmission across Chemical Synapses and Sweet Taste Signaling
HOXA1	homeo box A1	95297_at	1.3131	0.0324	Neural Crest Differentiation
IGHG2A	immunoglobulin heavy constant gamma 2A	101752_f_at	1.3640	0.0297	Antigen binding, immunoglobulin receptor binding
IQGAP1	IQ motif containing GTPase activating protein 1	93850_at	1.2924	0.0350	Adherens_junction
ITIH5	inter-alpha-trypsin inhibitor H5	161813_i_at	1.2966	0.0192	Extracellular matrix stabilization, prevention of tumor metastasis
KDM1	lysine-specific Demethylase 1A	98889_at	1.3059	0.0433	Histone demethylase, epigenetics, development, cancer progression
KEG1	kidney expressed gene 1	96938_at	1.4241	0.0006	Transferase activity
KIF1B	kinesin heavy chain member 1B	94379_at	1.4415	0.0142	Cytoskeletal Signaling and Reelin Pathway
KIF20A	Rab6, kinesin-like	161856_f_at	1.2802	0.0444	Class I MHC mediated antigen processing and presentation and Cell Cycle, Mitotic
KMT2A	lysine (K)-specific methyltransferase 2A	104568_at	1.2764	0.0392	Transcriptional misregulation in cancer and Senescence and Autophagy
KPNA1	karyopherin (importin) alpha 1	101370_at	1.3271	0.0485	Influenza_A
LCK	lymphocyte protein tyrosine kinase	102809_s_at	1.3097	0.0020	Natural_killer_cell_mediated_cytotoxicity
MAU2	MAU2 Sister Chromatid Cohesion Factor	104035_at	1.2963	0.0048	Among its related pathways are Cell Cycle, Mitotic and Cell Cycle, Mitotic
MOB2	Mob Kinase Activator 2	162467_r_at	1.2505	0.0123	Stimulates the autophosphorylation and kinase activity of STK38 and STK38L
NAA30	N(alpha)-acetyltransferase 30, NatC catalytic subunit	104404_at	1.3886	0.0479	Among its related pathways are Biological oxidations
NGP	neutrophilic granule protein	96153_at	1.3224	0.0013	Cysteine-type endopeptidase inhibitor activity, peptidase inhibitor activity
NKAIN1	Na+/K+ transporting ATPase interacting 1	162249_f_at	1.2542	0.0465	Interacts with the beta subunit of Na,K-ATPase (ATP1B1; MIM 182330)
OAS1	2'-5' oligoadenylate synthetase 1B	99383_at	1.2598	0.0098	Innate Immune response to viral infection
PBX3	pre B-cell leukemia transcription factor 3	96580_at	1.2650	0.0189	Transcriptional_misregulation_in_cancer
PCDHA10	protocadherin alpha 10	101769_at	1.2756	0.0371	Calcium ion binding
PECAM1	platelet/endothelial cell adhesion molecule	97830_at	1.2572	0.0077	Cell_adhesion_molecules_(CAMs), Leukocyte_transendothelial_migration
PEX6	peroxisomal biogenesis factor 6	99469_at	1.3068	0.0435	Peroxisome
PLA2G6	phospholipase A2, group VI	97965_at	1.2558	0.0431	Alpha_Linolenic_acid_metabolism, Arachidonic_acid_metabolism
PRPH1	peripherin	104751_at	1.2619	0.0216	Amyotrophic lateral sclerosis (ALS)
PSTPIP1	proline-serine-threonine phosphatase-interacting prot	161244_f_at	1.2804	0.0032	NOD_like_receptor_signaling_pathway
PYHIN1	pyrin and HIN domain family, member 1	103615_at	1.3732	0.0026	IFN inducible. Cell cycle control, Tumor suppressing activity
RABEP1	rabaptin 5	102724_at	1.2655	0.0360	Endocytosis
RPS24	ribosomal protein S24	93121_at	1.2840	0.0301	Ribosome
SCGB1B2	secretoglobin, family 1B	101621_at	1.2846	0.0099	Androgen-binding protein in mouse
SELPLG	selectin, platelet (p-selectin) ligand	103488_at	1.3296	0.0068	Hemostasis and amb2 Integrin signaling

SKIL	ski/sno related	94752_s_at	1.2674	0.0006	Signaling_pathways_regulating_pluripotency_of_stem_cells
SLC51A	solute carrier family 51, alpha subunit	160978_at	1.2820	0.0211	Bile secretion and Drug Induction of Bile Acid Pathway
SLC6A4	solute carrier family 6 (neurotransmitter transporter, s	99040_at	1.2923	0.0159	Serotonergic_synapse
STK3	serine/threonine kinase 3 (Ste20, yeast homolog)	98775_at	1.2896	0.0433	Hippo_signaling_pathway, MAPK_signaling_pathway
STMN3	stathmin-like 3	160170_at	1.3368	0.0037	Protein binding
SULT4A1	sulfotransferase family 4A, member 1	94564_at	1.2802	0.0159	Metabolism and Biological oxidations
TP53	transformation related protein 53	101617_s_at	1.3103	0.0204	Tumor suppressor, DNA damage
TRHR	thyrotropin releasing hormone receptor	97735_at	1.2664	0.0240	Calcium_signaling_pathway
TRIM47	tripartite motif-containing 47	161484_r_at	1.3197	0.0182	Metal ion binding
UBR4	ubiquitin protein ligase E3 component n-recognin 4	104041_at	1.2992	0.0379	Class I MHC mediated antigen processing and presentation
VCAM1	vascular cell adhesion molecule 1	92560_g_at	1.2720	0.0445	Cell_adhesion_molecules_(CAMs), Leukocyte_transendothelial_migration
ZC3H14	zinc finger CCCH type containing 14	97380_at	1.3480	0.0094	Poly(A)-binding proteins, regulating mRNA stability, nuclear export, and translation

Microarray gene analysis was conducted as described in the Method section. The probes used to study individual genes are listed along with the gene symbols and gene names. Some genes may have more than one probe. FC stands for fold of change over control (non-DEN treated). P values refer to the significance test. Genes listed in this table have FC of >1.25 with a *p* value <0.05. The function of the genes were obtained via multiple bioinformatics sources. Only main functions are listed. Not all genes have a clearly defined function.
