# Supplementary material for: Gene Expression Analysis Indicates Divergent Mechanisms in DEN-Induced Carcinogenesis in Wild Type and Bid-Deficient Livers
Source: PLoS One. 2016 May 19;11(5):e0155211. doi: 10.1371/journal.pone.0155211 (PMC4873180; doi:10.1371/journal.pone.0155211)
Supplement: S2 Table — (PDF) [file pone.0155211.s002.pdf]

S2 Table. Down-regualted genes in livers of wild type mice treated with DEN for 4-6 months

Genes Symbol	Gene Name	Probes	FC	p value	Function
1810058I24RIK	RIKEN cDNA 1810058I24 gene	100877_at	0.7221	0.0330	
2210039B01Rik	RIKEN cDNA 2210039B01 gene	100920_at	0.7491	0.0280	
AHR	aryl-hydrocarbon receptor	160495_at	0.5767	0.0239	AHR Pathway
AIM1	absent in melanoma 1	103443_at	0.7260	0.0313	May function as suppressor of malignant melanoma
AP2S1	adaptor-related protein complex 2, sigma 1 subunit	96638_at	0.7940	0.0450	Endocrine_regulated_calcium_reabsorption
ATXN2	ataxin 2	162399_f_at	0.6980	0.0139	Akt Signaling and Parkinsons Disease Pathway
AW112010	expressed sequence AW112010	100944_at	0.7000	0.0273	
C1QBP	complement component 1, q subcomponent binding protein	96856_at	0.7821	0.0083	Herpes_simplex_infection
CBX4	chromobox 4	93697_at	0.6200	0.0026	Cellular Senescence
CPT2	carnitine palmitoyltransferase 2	161978_r_at	0.7399	0.0468	Fatty_acid_degradation
CYP8B1	cytochrome P450, 8b1, sterol 12 alpha-hydrolase	103284_at	0.7326	0.0288	Primary_bile_acid_biosynthesis
D9ERTD720E	DNA segment, Chr 9, ERATO Doi 720, expressed	162412_r_at	0.7679	0.0423	
DAP	death-associated protein	93842_at	0.7781	0.0022	Akt Signaling
DCT	dopachrome tautomerase	103597_at	0.6596	0.0239	Melanogenesis, Tyrosine_metabolism
EIF1	eukaryotic translation initiation factor 1	92855_at	0.7436	0.0235	RNA_transport
ELL2	elongation factor RNA polymerase II 2	103891_i_at	0.7771	0.0165	regulation of transcription
GNG5	guanine nucleotide binding protein (G protein), gamma 5	94042_f_at	0.7803	0.0457	Alcoholism
HGD	homogentisate 1, 2-dioxygenase	98582_at	0.7892	0.0083	Tyrosine_metabolism
HSD17B10	hydroxysteroid (17-beta) dehydrogenase 10	101045_at	0.7453	0.0081	Metabolism
IDI1	isopentenyl-diphosphate delta isomerase	96269_at	0.6210	0.0436	Terpenoid_backbone_biosynthesis
LSM7	LSM7 homolog, U6 small nuclear RNA associated (S. cerevisiae)	97907_at	0.7591	0.0106	RNA_degradation, Splicesome
ROMO1	reactive oxygen species modulator 1	96687_f_at	0.7065	0.0326	Replicative cell aging, cell proliferation
SQLE	squalene epoxidase	94322_at	0.6707	0.0198	Steroid_biosynthesis
SRP19	signal recognition particle 19	160343_at	0.7521	0.0361	Protein_export
SULT1B1	sulfotransferase family 1B, member 1	104539_at	0.7671	0.0223	Metabolism and Biological oxidations
TDO2	tryptophan 2,3-dioxygenase	93827_at	0.6712	0.0173	Tryptophan_metabolism
ZBTB48	zinc finger and BTB domain containing 48	103765_at	0.7251	0.0085	DNA binding transcription factor activity

Microarray gene analysis was conducted as described in the Method section. The probes used to study individual genes are listed along with the gene symbols and gene names. Some genes may have more than one probe. FC stands for fold of change over control (non-DEN treated). P values refer to the significance test. Genes listed in this table have FC of <0.80 with a *p* value <0.05. The function of the genes were obtained via multiple bioinformatics sources. Only main functions are listed. Not all genes have a clearly defined function.
