# Supplementary material for: Gene Expression Analysis Indicates Divergent Mechanisms in DEN-Induced Carcinogenesis in Wild Type and Bid-Deficient Livers
Source: PLoS One. 2016 May 19;11(5):e0155211. doi: 10.1371/journal.pone.0155211 (PMC4873180; doi:10.1371/journal.pone.0155211)
Supplement: S3 Table — (PDF) [file pone.0155211.s003.pdf]

S3 Table. Up-regulated genes in livers of wild type mice treated with DEN for 10-12 months

Genes Symbol	Gene Name	Probes	FC	p value	Function
1200016E24RIK	RIKEN cDNA 1200016E24 gene	99849_at	2.3816	0.0016	
ABCC9	ATP-binding cassette, sub-family C (CFTR/MRP), member 9	97172_s_at	1.2941	0.0303	ABC_transporters
ACOT9	acyl-CoA thioesterase 9	160171_f_at	1.4619	0.0233	Acyl-CoA hydrolysis.
AF251705	cDNA sequence AF251705	104023_at	1.3161	0.0014	Immune system process, regulation of cytokine secretion
AGFG1	ArfGAP with FG repeats 1	103397_at	1.3484	0.0172	RNA binding
AIF1	allograft inflammatory factor 1	102330_at	1.3006	0.0178	Spinal Cord Injury
AKR1B7	aldo-keto reductase family 1, member B7	102826_at	2.1831	0.0456	Fructose_and_mannose_metabolism
Ank3	ankyrin 3, epithelial	98476_at	1.3431	0.0250	Proteoglycans in cancer and L1CAM interactions
ANXA1	annexin A1	161703_f_at	1.5460	0.0304	Signaling by GPCR
ANXA2	annexin A2	100569_at	2.3045	0.0066	Tyrosine Kinases / Adaptors and Ca, cAMP and Lipid Signaling
APBB1IP	amyloid beta (A4) precursor protein-binding, family B, member 1 intera	102710_at	1.5918	0.0440	Platelet_activation, Rap1_signaling_pathway
APCS	serum amyloid P-component	104072_at	2.6834	0.0151	Protein complex assembly
APLNR	apelin receptor	99368_at	1.2723	0.0289	Signaling by GPCR
APOA4	apolipoprotein A-IV	100078_at	4.5542	0.0043	Fat_digestion_and_absorption
APOC2	apolipoprotein CII	97887_at	2.2676	0.0090	Signaling by GPCR and Regulation of actin cytoskeleton
ARHGDIB	rho, GDP dissociation inhibitor (GDI) beta	94258_at	1.2653	0.0086	Neurotrophin_signaling_pathway
ARL2BP	ADP-ribosylation factor-like 2 binding protein	98084_at	1.2510	0.0395	transcription coactivator activity
ASNS	asparagine synthetase	95133_at	1.4587	0.0197	Alanine,_aspartate_and_glutamate_metabolism
ASXL1	additional sex combs like 1	104261_at	1.2708	0.0013	Transcription coactivator, retinoic acid receptor binding
ATPIF1	ATPase inhibitor	93984_at	1.5234	0.0096	Glucose / Energy Metabolism
BASP1	brain abundant, membrane attached signal protein 1	95673_s_at	1.8168	0.0006	Transcription corepressor activity
BCL2A1A	B-cell leukemia/lymphoma 2 related protein A1d	93869_s_at	1.9353	0.0232	NF_kappa_B_signaling_pathway
BIN1	Bridging Integrator 1	99668_at	1.2545	0.0460	Arf6 trafficking, Delta508-CFTR traffic / ER-to-Golgi in CF
BIRC5	baculoviral IAP repeat-containing 5	101521_at	1.3447	0.0143	Hippo_signaling_pathway
BLNK	lymphocyte antigen 57	100771_at	1.3264	0.0037	B_cell_receptor_signaling_pathway
BTG2	B-cell translocation gene 2, anti-proliferative	101583_at	2.0186	0.0379	RNA_degradation
BTG3	B-cell translocation gene 3	96146_at	1.9401	0.0278	RNA_degradation
BZW2	basic leucine zipper and W2 domains 2	95462_at	1.2880	0.0239	Multicellular organismal development
C1QA	complement component 1, q subcomponent, alpha polypeptide	98562_at	1.6453	0.0269	Complement_and_coagulation_cascades
C1QB	complement component 1, q subcomponent, beta polypeptide	96020_at	2.0625	0.0271	Complement_and_coagulation_cascades
C1QC	complement component 1, q subcomponent, c polypeptide	92223_at	1.7362	0.0065	Complement_and_coagulation_cascades
CALML4	calmodulin-like 4	96203_at	1.5490	0.0001	Calcium ion binding
CAPG	capping protein (actin filament), gelsolin-like	160106_at	1.3864	0.0019	Actin binding
CCDC86	coiled-coil domain containing 86	103768_at	1.3513	0.0486	Poly(A) RNA binding
CCL4	small inducible cytokine A4	94146_at	1.2846	0.0351	TLR_signaling_pathway, Chemokine_signaling_pathway
CCL5	small inducible cytokine A5	98406_at	1.4894	0.0475	TNF_signaling_pathway, Chemokine_signaling_pathway
CCR2	chemokine (C-C) receptor 2	93397_at	1.3375	0.0066	Chemokine_signaling_pathway
CCRL2	chemokine (C-C) receptor 1,-like 2	93617_at	1.2580	0.0183	Signaling by GPCR and Akt Signaling
CD14	CD14 antigen	98088_at	2.0174	0.0178	TLR_signaling_pathway
CD24	CD24a antigen	100600_at	1.3833	0.0406	Hematopoietic cell lineage
CD2AP	CD2-associated protein	103281_at	1.2718	0.0369	Bacterial_invasion_of_epithelial_cells
CD63	CD63 antigen	160493_at	3.3195	0.0148	Lysosome
CD68	CD68 antigen	103016_s_at	1.4785	0.0030	Lysosome
CD83	CD83 antigen	103040_at	1.3748	0.0159	B cell receptor signaling pathway
CD99	CD99 antigen	101047_at	1.6316	0.0426	Leukocyte_transendothelial-migration, Cell_adhesion_molecules
CDC20	cell division cycle 20 homolog (S. cerevisiae)	96319_at	1.3050	0.0177	Cell_cycle
CIITA	class II transactivator	102917_at	1.2789	0.0190	Antigen_processing_and_presentation
CLCA1	chloride channel calcium activated 1	103812_at	1.8329	0.0072	Pancreatic_secretion
CNN2	calponin 2	94004_at	1.4834	0.0078	Myometrial Relaxation and Contraction Pathways
COL1A1	procollagen, type I, alpha 1	94305_at	1.5132	0.0089	ECM_receptor_interaction, Focal_adhesion

COL5A2	procollagen, type V, alpha 2	92567_at	1.4976	0.0072	ECM_receptor_interaction, Focal_adhesion
COX6B2	cytochrome c oxidase subunit VIb polypeptide 2	97351_i_at	1.3099	0.0154	Oxidative_phosphorylation
CP	ceruloplasmin	92851_at	1.6553	0.0303	Porphyrin_and_chlorophyll_metabolism
CPE	carboxypeptidase E	99642_i_at	9.2062	0.0098	Maturation of insulin and other hormones, neurotransmitters
CPE	carboxypeptidase E	99643_f_at	6.1898	0.0162	Maturation of insulin and other hormones, neurotransmitters
CRIP1	cysteine rich intestinal protein	94061_at	1.4416	0.0032	DNA binding, bending and AT DNA binding
CRYGD	crystallin, gamma D	162268_at	1.2786	0.0479	Structural constituent of eye lens
CTLA2B	cytotoxic T lymphocyte-associated protein 2 beta	103518_at	1.5725	0.0161	Cysteine-type endopeptidase inhibitor, cysteine-type peptidase
CTPS2	cytidine 5'-triphosphate synthase 2	160652_at	1.4708	0.0429	Pyrimidine_metabolism
CTSD	cathepsin D	93810_at	1.3552	0.0209	Lysosome
CTSS	cathepsin S	98543_at	2.0519	0.0108	Antigen_processing_and_presentation
CYBA	cytochrome b-245, alpha polypeptide	100059_at	1.4238	0.0014	Leukocyte_transendothelial_migration
CYBA	cytochrome b-245, alpha polypeptide	97013_f_at	1.5608	0.0332	Leukocyte_transendothelial_migration
CYP17A1	cytochrome P450, 17	102416_at	1.5921	0.0315	Ovarian_steroidogenesis
CYP2A4	cytochrome P450, 2a4	102847_s_at	2.8130	0.0045	Retinol_metabolism
CYP2B9	cytochrome P450, 2b9, phenobarbitol inducible, type a	101862_at	2.9224	0.0355	Arachidonic_acid_metabolism
D17H6S56E-5	G7e protein	104333_at	2.1844	0.0484	
DBNDD2	dysbindin (dystrobrevin binding protein 1) domain containing 2	99641_at	1.3450	0.0112	Negative regulation of protein kinase activity
DDIT3	DNA-damage inducible transcript 3	101429_at	1.3794	0.0143	MAPK_signaling_pathway
DDR1	Discoidin Domain Receptor Tyrosine Kinase 1	100155_at	1.6467	0.0171	GPCR Pathway and Akt Signaling
Dhcr24	24-dehydrocholesterol reductase	102925_at	1.2746	0.0003	MAPK_signaling_pathway
DLGAP4	discs, large homolog-associated protein 4 (Drosophila)	104136_at	1.2534	0.0126	Biological_process, signaling
DMPK	dystrophia myotonica kinase, B15	93431_at	1.3775	0.0165	Protein S/T kinase activity and myosin phosphatase regulation
DNAJB4	DnaJ heat shock protein family (Hsp40) member B4	93853_at	1.2640	0.0472	Protein folding
DNTT	deoxynucleotidyltransferase, terminal	103962_at	1.2917	0.0254	Hematopoietic cell lineage
DRC1	dynein regulatory complex subunit 1	92377_at	1.3962	0.0157	Embryonic development
ELL2	elongation factor RNA polymerase II 2	97704_at	1.3041	0.0194	Regulation of transcription, DNA-templated
EMP1	epithelial membrane protein 1	97426_at	1.4417	0.0445	Cell death, cell growth, bleb assembly
EMP3	epithelial membrane protein 3	93593_f_at	1.2986	0.0183	Cell death, cell growth, bleb assembly
ENTPD6	ectonucleoside triphosphate diphosphohydrolase 6	103965_at	1.3157	0.0075	Purine_metabolism
ERDR1	erythroid differentiation regulator 1	98525_f_at	3.8401	0.0086	Cell proliferation, cell migration, somatic stem cell maintenance
Ero1l	ERO1-like (S. cerevisiae)	97871_at	1.2906	0.0369	protein disulfide isomerase activity, oxidoreductase activity
FABP4	fatty acid binding protein 4, adipocyte	100567_at	1.3364	0.0057	PPAR_signaling_pathway
FABP5	fatty acid binding protein 5, epidermal	160544_at	1.6181	0.0425	PPAR_signaling_pathway
FAM105A	family with sequence similarity 105, member A	95940_f_at	1.2866	0.0222	
FCGR1	Fc receptor, IgG, high affinity I	102879_s_at	1.3349	0.0079	Fc_gamma_R_mediated_phagocytosis
FHIT	fragile histidine triad gene	102628_at	1.2663	0.0363	Purine_metabolism
FKBP5	FK506 binding protein 5 (51 kDa)	94297_at	1.5658	0.0233	Estrogen_signaling_pathway
FLOT1	flotillin 1	95095_at	1.2652	0.0112	Insulin_signaling_pathway
FOS	FBJ osteosarcoma oncogene	160901_at	2.3221	0.0257	Amphetamine_addiction
FSTL1	follistatin-like	94833_at	1.2559	0.0227	Signaling by GPCR and TGF-beta Signaling Pathway
G6PD2	glucose-6-phosphate dehydrogenase 2	101293_at	1.3034	0.0224	Pentose_phosphate_pathway
G6PDX	glucose-6-phosphate dehydrogenase X-linked	94966_at	1.5792	0.0211	Pentose_phosphate_pathway
GADD45A	growth arrest and DNA-damage-inducible 45 alpha	102292_at	1.4416	0.0383	Cell_cycle
GAPDHS	glyceraldehyde-3-phosphate dehydrogenase, spermatogenic	160950_at	1.2941	0.0401	Glycolysis_/_Gluconeogenesis
GOLM1	golgi membrane protein 1	95593_at	1.4610	0.0425	Nucleus organization, regulation of lipid metabolic process
GSTM1	glutathione S-transferase, mu 1	102094_f_at	1.4734	0.0015	Glutathione_metabolism
GSTM1	glutathione S-transferase, mu 1	93543_f_at	1.5452	0.0032	Glutathione_metabolism
GSTM3	glutathione S-transferase, mu 3	97681_f_at	2.1009	0.0037	Glutathione_metabolism
GSTM3	glutathione S-transferase, mu 3	97682_r_at	2.1686	0.0041	Glutathione_metabolism
H2-M1	Mouse MHC class I H2-Qa-Mb1 gene, complete cds	101299_at	1.2727	0.0025	Allograft_rejection
HCK	hemopoietic cell kinase	93483_at	1.4239	0.0146	Fc_gamma_R_mediated_phagocytosis
HMOX1	heme oxygenase (decycling) 1	160101_at	1.4427	0.0183	Porphyrin_and_chlorophyll_metabolism

IER3	immediate early response 3	94384_at	1.8897	0.0308	Protein binding
IFI27	interferon, alpha-inducible protein 27	93775_at	1.6270	0.0278	Interferon Signaling and Interferon Signaling
IGFBP1	insulin-like growth factor binding protein 1	103896_f_at	3.1895	0.0276	N-glycan synthesis
IGH-VJ558	immunoglobulin heavy chain (J558 family)	100583_at	1.5793	0.0421	Immune system process
IL18BP	interferon gamma inducing factor binding protein	92689_at	1.3204	0.0035	NOD-like Receptor Signaling Pathways
INHBB	inhibin beta-B	160828_at	2.0703	0.0453	Cytokine_cytokine_receptor_interaction
IQGAP1	IQ Motif Containing GTPase Activating Protein 1	104300_at	1.7785	0.0221	Adherens_junction
IRF7	interferon regulatory factor 7	104669_at	1.4708	0.0077	TLR_signaling_pathway, Cytosolic_DNA_sensing_pathway
IRF7	interferon regulatory factor 7	162202_f_at	1.2999	0.0464	TLR_signaling_pathway, Cytosolic_DNA_sensing_pathway
ISYNA1	myo-inositol 1-phosphate synthase A1	160337_at	1.4576	0.0358	Inositol_phosphate_metabolism
ITGB2	integrin beta 2	102353_at	1.4139	0.0120	Cell_adhesion_molecules_(CAMs)
JUNB	Jun-B oncogene	102362_i_at	1.4955	0.0079	Osteoclast_differentiation
KLF4	Kruppel-like factor 4 (gut)	99622_at	1.5269	0.0058	Signaling_pathways_regulating_pluripotency_of_stem_cells
KLK1B4	nerve growth factor, alpha	94773_at	1.5075	0.0381	Inflammatory_mediator_regulation_of_TRP_channels
KLRB1B	killer cell lectin-like receptor subfamily B member 1B	94744_at	1.5858	0.0247	Negative regulation of natural killer cell mediated cytotoxicity
Krt8	keratin 8	101009_at	1.3893	0.0147	Cytoskeletal Signaling and EGFR1 Signaling Pathway
LAPTM5	lysosomal-associated protein transmembrane 5	100012_at	1.3768	0.0204	Lysosome
LCN2	lipocalin 2	160564_at	10.0780	0.0026	Transport small hydrophobic molecules
LILR4b	leukocyte immunoglobulin-like receptor, subfamily B, member 4B	100325_at	2.1257	0.0130	
LILR4A	leukocyte immunoglobulin-like receptor, subfamily B, member 4A	92217_s_at	1.3451	0.0107	Adaptive immune response, immune system process
LMNA	lamin A	98059_s_at	1.7219	0.0244	Arrhythmogenic_right_ventricular_cardiomyopathy_(ARVC)
LPIN1	lipin 1	98892_at	1.8654	0.0195	Glycerolipid_metabolism
LTB	lymphotoxin B	102940_at	1.3263	0.0207	Cytokine_cytokine_receptor_interaction
LY6A	lymphocyte antigen 6 complex, locus A	93078_at	1.5480	0.0322	Lymphocyte development
LY6D	lymphocyte antigen 6 complex, locus D	160553_at	3.9520	0.0063	Lymphocyte development
LY6E	lymphocyte antigen 6 complex, locus E	101487_f_at	2.0347	0.0206	AKT signaling
LY86	lymphocyte antigen 86	94425_at	1.3687	0.0012	Immune system process,inflammatory response
LYSMD2	LysM, putative peptidoglycan-binding, domain containing 2	103560_at	1.3249	0.0044	
LYVE1	lymphatic vessel endothelial hyaluronan receptor 1	160583_at	1.4572	0.0471	Disease and Metabolism
MARCKS	myristoylated alanine rich protein kinase C substrate	96865_at	1.5149	0.0145	MicroRNAs in cancer and Metabolism
MARCO	macrophage receptor with collagenous structure	102974_at	1.8721	0.0242	Phagosome
METTL1	methyltransferase-like 1 (S. cerevisiae)	99499_at	1.3099	0.0145	tRNA binding and tRNA (guanine-N7-)-methyltransferase activity
MFGE8	milk fat globule-EGF factor 8 protein	92880_at	1.5729	0.0078	Disease and Integrins in angiogenesis
MLLT3	myeloid/lymphoid or mixed-lineage leukemia (trithorax homolog, Drosc	103925_at	1.6008	0.0161	Transcriptional_misregulation_in_cancer
MMP12	matrix metalloproteinase 12	95339_r_at	1.4252	0.0183	GPCR Pathway and Integrin Pathway
MPEG1	macrophage expressed gene 1	99071_at	2.2921	0.0230	Cell Cycle
MS4A6B	membrane-spanning 4-domains, subfamily A, member 6B	102104_f_at	1.2962	0.0448	
MSH2	mutS homolog 2 (E. coli)	100033_at	1.2760	0.0020	Colorectal_cancer
MSR1	macrophage scavenger receptor 1	94792_at	1.4916	0.0025	Phagosome
MSX2	homeo box, msh-like 2	102956_at	1.2882	0.0307	Neural Crest Differentiation and HTLV-I infection
MT1A	metallothionein 1	93573_at	3.4107	0.0443	Platinum Pathway, Pharmacokinetics/Pharmacodynamics
MVK	mevalonate kinase	95632_f_at	1.2737	0.0477	Terpenoid_backbone_biosynthesis , Peroxisome
MVP	major vault protein	103793_at	1.3361	0.0040	protein kinase binding and protein phosphatase binding
MYL9	transient receptor protein 2	96939_at	1.3586	0.0500	Leukocyte_transendothelial_migration, cAMP_signaling_pathway
MYO1F	myosin If	101708_at	1.2629	0.0095	RhoGDI Pathway
NAPSA	kidney-derived aspartic protease-like protein	101972_at	1.3352	0.0139	Lysosome
NFKB2	nuclear factor of kappa light polypeptide gene enhancer in B-cells 2, p	103614_at	1.4285	0.0185	NF_kappa_B_signaling_pathway
Noct	nocturnin	97181_f_at	1.5862	0.0385	RNA binding, nuclease activity
NPPA	natriuretic peptide precursor type A	103593_at	1.2868	0.0224	HIF_1_signaling_pathway
NQO1	diaphorase 4 (NADH/NADPH)	94350_f_at	1.9863	0.0340	Ubiquinone_and_other_terpenoid_quinone_biosynthesis
NQO1	diaphorase 4 (NADH/NADPH)	94351_r_at	1.7474	0.0411	Ubiquinone_and_other_terpenoid_quinone_biosynthesis
NR1D1	eosinophil-associated ribonuclease 1	100311_f_at	1.4136	0.0008	Circadian_rhythm
NR2F6	eosinophil-associated ribonuclease 3	103240_f_at	1.3725	0.0006	Gene Expression

NR3C1	nuclear receptor subfamily 3, group C, member 1	98818_at	1.3173	0.0155	PEDF Induced Signaling
NTMT1	N-terminal Xaa-Pro-Lys N-methyltransferase 1	104219_f_at	1.2887	0.0434	Protein methyltransferase activity
OXCT1	3-oxoacid CoA transferase 1	92845_at	1.2873	0.0018	3-oxoacid CoA-transferase activity
PDZK1	PDZ domain containing 1	97287_at	1.3895	0.0407	Regulation of CFTR activity
PGD	phosphogluconate dehydrogenase	95420_at	1.3126	0.0064	Glutathione_metabolism, Pentose_phosphate_pathway
PGM1	phosphoglucomutase 1	104534_at	1.3462	0.0461	Amino_sugar_and_nucleotide_sugar_metabolism
PHLDA3	pleckstrin homology-like domain, family A, member 3	98056_at	1.3466	0.0041	Cell Cycle / Checkpoint Control, Apoptosis, Autophagy
PKM	pyruvate kinase, muscle	96066_s_at	1.5758	0.0271	Metabolism and Carbon metabolism
PLA2G7	phospholipase A2 group VII (platelet-activating factor acetylhydrolase,	101923_at	1.5724	0.0176	Ether_lipid_metabolism
PLAC8	placenta-specific 8	98092_at	1.9026	0.0196	Chromatin binding
PLAUR	plasminogen activator, urokinase receptor	102663_at	1.3212	0.0264	Complement_and_coagulation_cascades
PLD4	phospholipase D family, member 4	103299_at	1.2655	0.0006	Ether_lipid_metabolism; Glycerophospholipid_metabolism
PLK3	polo-like kinase 3	161636_r_at	1.3608	0.0069	FoxO_signaling_pathway
POLD4	polymerase (DNA-directed), delta 4	94843_at	1.7665	0.0227	DNA repair, replication, nucleotide metabolism
POSTN	periostin, osteoblast specific factor	92593_at	1.5885	0.0044	Heparin binding
PQLC3	PQ loop repeat containing	99366_at	1.8376	0.0006	Dolichol-linked oligosaccharide biosynthetic process
PRKCQ	protein kinase C, theta	104431_at	1.2634	0.0411	Adipocytokine_signaling_pathway
PSAT1	phosphoserine aminotransferase 1	96295_at	2.8280	0.0166	Glycine,serine, threonine metabolism, VitB6 metabolism
PTP4A3	Protein Tyrosine Phosphatase Type IVA, Member 3	160862_at	1.2709	0.0379	PAK Pathway and Signaling events mediated by PRL
RAB11FIP5	RAB11 family interacting protein 5 (class I)	95618_at	1.2578	0.0455	Endocytosis
RAC2	RAS-related C3 botulinum substrate 2	103579_at	1.2760	0.0174	Fc_gamma_R_mediated_phagocytosis, Adherens_junction
Rad51b	RAD51 homolog B	103944_at	1.7269	0.0121	Hemostasis and Homologous recombination
RASA4	RAS p21 protein activator 4	160965_at	1.3670	0.0115	Ras_signaling_pathway
RBP1	retinol binding protein 1, cellular	104716_at	2.5231	0.0359	Signaling by GPCR and Disease
RFX1	regulatory factor X, 1 (influences HLA class II expression)	99880_at	1.2868	0.0063	DNA binding transcription factor activity
RGS16	regulator of G-protein signaling 16	161609_at	1.5626	0.0228	Activation of cAMP-Dependent PKA and G-AlphaQ Signaling
RGS16	regulator of G-protein signaling 16	94378_at	2.1186	0.0195	Activation of cAMP-Dependent PKA and G-AlphaQ Signaling
RGS19	regulator of G-protein signaling 19 interacting protein 1	103605_g_at	1.3215	0.0001	Signaling by GPCR and Activation of cAMP-Dependent PKA
RHOC	aplysia ras-related homolog 9 (RhoC)	96056_at	1.5844	0.0141	RhoGDI Pathway and Signaling by GPCR
RHOJ	ras homolog gene family, member J	104697_at	1.3572	0.0332	Signaling by GPCR and Akt Signaling
RNF14	ring finger protein 14	93958_at	1.3762	0.0499	Androgen receptor signaling pathway
ROCK2	Rho-associated coiled-coil forming kinase 2	98504_at	1.7854	0.0040	Leukocyte_transendothelial_migration, cAMP_signaling_pathway
ROGDI	rogdi homolog (Drosophila)	97228_at	1.2999	0.0313	Kohlschutter-tonz syndrome and focal epilepsy
RSAD2	radical S-adenosyl methionine domain containing 2	104177_at	1.6761	0.0279	Influenza_A
RUNDC3A	RUN Domain Containing 3A	103960_at	1.2765	0.0448	Regulation of cGMP biosynthesis and guanylate cyclase activity
S100A11	S100 calcium binding protein A11	98600_at	2.4067	0.0163	Calcium ion binding and calcium-dependent protein binding
S100A6	S100 calcium binding protein A6 (calcyclin)	92770_at	1.8826	0.0024	Prostaglandin synthesis and regulation
SCN1B	sodium channel, voltage-gated, type I, beta polypeptide	102808_at	1.3253	0.0281	Adrenergic_signaling_in_cardiomyocytes
SCXA	UI-M-BH2.1-apr-e-01-0-UI.s1 Mus musculus cDNA, 3' end	161030_at	1.2792	0.0415	
SEC63	SEC63-like (S. cerevisiae)	99350_at	1.6208	0.0389	Protein_processing_in_endoplasmic_reticulum
SELPL	selectin, platelet (p-selectin) ligand	103488_at	1.3476	0.0048	Cell adhesion
SIRPA	protein tyrosine phosphatase, non-receptor type substrate 1	103070_at	1.3555	0.0353	Osteoclast_differentiation
SLC11A1	solute carrier family 11 (proton-coupled divalent metal ion transporters	96562_at	1.3166	0.0003	Lysosome
SLC1A4	solute carrier family 1 (glutamate/neutral amino acid transporter), mem	100943_at	1.2640	0.0457	Transport of glucose and other sugars, bile salts and organic acids
SLC7A7	solute carrier family 7 (cationic amino acid transporter, y+ system), me	103818_at	1.4807	0.0249	Protein_digestion_and_absorption
SLFN2	schlafen 2	92471_i_at	1.6295	0.0172	Negative regulation of cell proliferation
SLFN2	schlafen 2	92472_f_at	1.5948	0.0232	Negative regulation of cell proliferation
SNTB1	syntrophin, basic 1	93384_at	1.2758	0.0380	Protein binding
SOCS1	cytokine inducible SH2-containing protein 1	92832_at	1.2732	0.0202	Insulin_signaling_pathway
SOD3	superoxide dismutase 3, extracellular	94902_at	1.3409	0.0089	Cellular Senescence and Selenium Pathway
SOX3	SRY (sex determining region Y)-box 3	161276_i_at	1.3430	0.0207	ERK Signaling
SPI1	SFFV proviral integration 1	102641_at	1.2514	0.0231	Acute_myeloid_leukemia
SPSB1	splA/ryanodine receptor domain and SOCS box containing 1	161013_f_at	1.2841	0.0472	Class I MHC mediated antigen processing and presentation

SPTLC2	serine palmitoyltransferase, long chain base subunit 2	100893_at	1.3959	0.0027	Sphingolipid_metabolism
SSR3	signal sequence receptor, gamma	104248_at	1.3515	0.0367	Protein_processing_in_endoplasmic_reticulum
SST	somatostatin	95436_at	1.3604	0.0096	Gastric_acid_secretion
ST8SIA4	ST8 alpha-N-acetyl-neuraminide alpha-2,8-sialyltransferase 4	102318_at	1.3101	0.0414	L1CAM interactions
STAG1	stromal antigen 1	92478_at	1.2725	0.0067	Cell_cycle
TAGLN2	transgelin 2	160162_at	1.5434	0.0223	Epithelial cell differentiation
TCF20	transcription factor 20	100947_at	1.4135	0.0178	ERK Signaling
TENM4	teneurin transmembrane protein 4	98313_at	1.3088	0.0227	Protein homodimerization activity
TMEM176B	transmembrane protein 176B	97885_at	1.5539	0.0193	Cell differentiation, regulation of dendritic cell differentiation
TMEM71	transmembrane protein 71	93963_at	1.5543	0.0122	
TNFSF12	tumor necrosis factor (ligand) superfamily, member 12	93917_at	1.3354	0.0002	Cytokine_cytokine_receptor_interaction
TPM4	tropomyosin 4	95543_at	1.2748	0.0030	Adrenergic_signaling_in_cardiomyocytes
TRAF1	Tnf receptor-associated factor 1	94186_at	1.2652	0.0095	Epstein_Barr_virus_infection
TWF2	Twinfilin Actin-Binding Protein 2	94020_at	1.5411	0.0236	Signaling by GPCR and Insulin receptor signalling cascade
UBD	ubiquitin D	92715_at	1.3903	0.0311	Signaling by GPCR and NF-KappaB Family Pathway
USP18	ubiquitin specific peptidase 18	95024_at	2.1371	0.0024	Interferon Signaling
VCAM1	vascular cell adhesion molecule 1	92559_at	1.4762	0.0053	Leukocyte_transendothelial_migration
VWF	Von Willebrand factor homolog	103499_at	1.2766	0.0354	Complement_and_coagulation_cascades
WLS	wntless homolog (Drosophila)	101001_at	1.2952	0.0300	GPCR and beta-catenin independent WNT signaling
YBX1	Y box protein 1	95366_at	1.5283	0.0319	Signaling events mediated by PTP1B and Translational Control
ZBTB16	zinc finger and BTB domain containing 16	92202_g_at	1.7482	0.0276	Transcriptional_regulation
ZFP622	zinc finger protein 622	161927_at	1.4038	0.0041	Cell death
ZFP771	zinc finger protein 771	162310_r_at	1.3407	0.0420	Nucleic acid binding, DNA binding, metal ion binding

Microarray gene analysis was conducted as described in the Method section. The probes used to study individual genes are listed along with the gene symbols and gene names. Some genes may have more than one probe. FC stands for fold of change over control (non-DEN treated). P values refer to the significance test. Genes listed in this table have FC of >1.25 with a *p* value <0.05. The function of the genes were obtained via multiple bioinformatics sources. Only main functions are listed. Not all genes have a clearly defined function.

Genes shown in red font are related to immune response and inflammation pathways, and are further detailed in S10 Table. Not all such genes are highlighted.
