# Supplementary material for: Gene Expression Analysis Indicates Divergent Mechanisms in DEN-Induced Carcinogenesis in Wild Type and Bid-Deficient Livers
Source: PLoS One. 2016 May 19;11(5):e0155211. doi: 10.1371/journal.pone.0155211 (PMC4873180; doi:10.1371/journal.pone.0155211)
Supplement: S4 Table — (PDF) [file pone.0155211.s004.pdf]

S4 Table. Down-regulated genes in livers of wild type mice treated with DEN for 10-12months

Genes Symbol	Gene Name	Probes	FC	p value	Function
0610005C13RIK	RIKEN cDNA 0610005C13 gene	104617_at	0.7452	0.0062	
1110001J03RIK	RIKEN cDNA 1110001J03 gene	94907_f_at	0.5614	0.0053	
1110001J03RIK	RIKEN cDNA 1110001J03 gene	94908_r_at	0.6245	0.0074	
1110032A03RIK	RIKEN cDNA 1110032A03 gene	104314_r_at	0.6252	0.0184	
1190005I06RIK	RIKEN cDNA 1190005I06 gene	93143_at	0.6877	0.0187	
2310010J17RIK	RIKEN cDNA 2310010J17 gene	161018_at	0.7521	0.0238	
AADAC	arylacetamide deacetylase (esterase)	95439_at	0.5724	0.0400	Deacetylase activity
AADAT	aminoadipate aminotransferase	98123_at	0.4832	0.0034	Lysine_metabolism, Tryptophan_metabolism
AASS	lysine oxoglutarate reductase, saccharopine dehydrogenase	103389_at	0.6982	0.0154	Lysine_degradation
AATK	apoptosis-associated tyrosine kinase	100994_at	0.7721	0.0400	Protein S/T kinase activity
ABCC6	ATP-binding cassette, sub-family C (CFTR/MRP), member 6	93407_at	0.6301	0.0028	ABC_transporters
ABCD3	ATP-binding cassette, sub-family D (ALD), member 3	93045_at	0.5817	0.0263	ABC_transporters
ACAA2	acetyl-Coenzyme A acyltransferase 2 (mitochondrial 3-oxoacyl-Coenzyme A thiolase)	95064_at	0.7361	0.0450	Fatty_acid_Valine_leucine_isoleucine_degradation
ACADL	acetyl-Coenzyme A dehydrogenase, long-chain	95425_at	0.6559	0.0412	Fatty_acid_degradation
ACOT8	acyl-CoA thioesterase 8	103386_at	0.7811	0.0150	Primary_bile_acid_biosynthesis, Peroxisome
ACOX1	acyl-Coenzyme A oxidase 1, palmitoyl	101515_at	0.5265	0.0057	Alpha_Linolenic_acid_metabolism
ACSL1	fatty acid Coenzyme A ligase, long chain 2	94507_at	0.4094	0.0019	Adipocytokine_signaling_pathway
ACYP1	acylphosphatase 1, erythrocyte (common) type	93560_at	0.7412	0.0425	Pyruvate_metabolism
ADCY9	adenylate cyclase 9	98356_at	0.7514	0.0077	Signaling by FGFR
ADH4	alcohol dehydrogenase 4 (class II), pi polypeptide	103982_s_at	0.3703	0.0052	Drug_metabolism_cytochrome_P450
ADH4	alcohol dehydrogenase 4 (class II), pi polypeptide	103983_at	0.4651	0.0255	Drug_metabolism_cytochrome_P450
ADIPOR2	adiponectin receptor 2	104605_at	0.5921	0.0063	Adipocytokine_signaling_pathway
AGXT	alanine-glyoxylate aminotransferase	93625_at	0.5890	0.0343	Alanine,_aspartate_and_glutamate_metabolism
AHCY	S-adenosylhomocysteine hydrolase	96025_g_at	0.6829	0.0338	Cysteine_and_methionine_metabolism
AI464131	expressed sequence AI464131	104034_at	0.7073	0.0028	Carbohydrate_metabolism
AK2	adenylate kinase 2	95148_at	0.6930	0.0443	Purine_metabolism
AK6	adenylate kinase 6	104356_at	0.7858	0.0243	Basal_transcription_factors
AKAP1	A kinase (PRKA) anchor protein 1	97367_at	0.5783	0.0064	Activation of cAMP-Dependent PKA
AKR1C12	aldo-keto reductase family 1, member C12	95015_at	0.7064	0.0300	Xenobiotic metabolic process
AKR1C6	aldo-keto reductase family 1, member C6	92556_at	0.5849	0.0319	Lipid metabolic process
ALAD	aminolevulinate, delta-, dehydratase	101044_at	0.6406	0.0427	Porphyrin_and_chlorophyll_metabolism
ALDH1A1	aldehyde dehydrogenase family 1, subfamily A1	100068_at	0.5231	0.0489	Retinol_metabolism
ALDH2	aldehyde dehydrogenase 2, mitochondrial	161997_f_at	0.6511	0.0121	Alcohol metabolism, amino acid_metabolism
ALDH2	aldehyde dehydrogenase 2, mitochondrial	96057_at	0.7252	0.0247	Alcohol metabolism, amino acid_metabolism
ALDH2	aldehyde dehydrogenase 2, mitochondrial	96058_s_at	0.5810	0.0485	Alcohol metabolism, amino acid_metabolism
ALDH7A1	aldehyde dehydrogenase family 7, member A1	97449_at	0.6689	0.0263	Alcohol metabolism, Lysine_degradation
ALDH7A1	aldehyde dehydrogenase family 7, member A1	97450_s_at	0.6157	0.0438	Alcohol metabolism, Lysine_degradation
AMACR	alpha-methylacyl-CoA racemase	95588_at	0.5786	0.0052	Primary_bile_acid_biosynthesis, Peroxisome
Ammecr1l	AMME chromosomal region gene 1-like	103082_at	0.6529	0.0018	
AMY1	amylase 1, salivary	101058_at	0.5141	0.0305	Carbohydrate metabolism
ANGPT1	angiogenin	94392_f_at	0.4304	0.0498	PI3K_Akt_signaling, HIF1_signaling, Ras_signaling
ANKRD28	ankyrin repeat domain 28	93627_at	0.7737	0.0015	Membrane trafficking
ANKRD33B	ankyrin repeat domain 33B	104206_at	0.7502	0.0421	DNA binding
ANKRD46	ankyrin repeat domain 46	93056_g_at	0.6107	0.0050	
ANP32A	acidic (leucine-rich) nuclear phosphoprotein 32 family, member A	104390_at	0.7493	0.0039	Gene Expression and Granzyme Pathway
AOX1	aldehyde oxidase 1	104011_at	0.6427	0.0125	Drug_metabolism_cytochrome_P450
AQP9	aquaporin 9	104328_at	0.4900	0.0056	Bile_secretion
ARID1A	AT rich interactive domain 1A (SWI-like)	104330_g_at	0.7718	0.0025	Activation of the ESR1/SP pathway
ARID1A	AT rich interactive domain 1A (SWI-like)	104331_at	0.7369	0.0195	Activation of the ESR1/SP pathway
ARNTL	aryl hydrocarbon receptor nuclear translocator-like	102382_at	0.6636	0.0316	Circadian_rhythm
ATF2	activating transcription factor 2	162010_r_at	0.7155	0.0123	Adrenergic_signaling_in_cardiomyocytes

ATG5	autophagy Related 5	94225_at	0.7469	0.0346	Regulation_of_autophagy
ATP2A2	ATPase, Ca++ transporting, cardiac muscle, slow twitch 2	99570_s_at	0.7943	0.0434	Adrenergic_signaling_in_cardiomyocytes
ATP5C1	ATP synthase, H+ transporting, mitochondrial F1 complex, gamma polypeptide 1	92798_at	0.6785	0.0155	Oxidative_phosphorylation
ATP5C1	ATP synthase, H+ transporting, mitochondrial F1 complex, gamma polypeptide 1	92800_i_at	0.6742	0.0473	Oxidative_phosphorylation
ATP5K	ATP synthase, H+ transporting, mitochondrial F1F0 complex, subunit E	102561_at	0.6407	0.0122	hydrogen ion transmembrane transporter activity
ATP5L	ATP synthase, H+ transporting, mitochondrial F0 complex, subunit g	93014_at	0.7797	0.0190	Oxidative_phosphorylation
ATXN2	ataxin 2	162399_f_at	0.5766	0.0002	Akt Signaling and Parkinsons Disease Pathway
ATXN2	ataxin 2	93535_at	0.6840	0.0138	Akt Signaling and Parkinsons Disease Pathway
ATXN7L3b	ataxin 7-like 3B	99645_at	0.6371	0.0204	Transcription regulatory histone acetylation
AUH	AU RNA binding protein/enoyl-coenzyme A hydratase	96650_at	0.7938	0.0044	Valine,_leucine_and_isoleucine_degradation
AZGP1	Alpha-2-Glycoprotein 1, Zinc-Binding	96867_at	0.6102	0.0262	Antigen binding and ribonuclease activity
BAAT	bile acid-Coenzyme A: amino acid N-acyltransferase	104273_at	0.5687	0.0012	Bile_metabolis, _metabolism
BCAP31	B-cell receptor-associated protein 31	93252_at	0.7662	0.0362	Protein_processing_in_endoplasmic_reticulum
BCKDHA	branched chain ketoacid dehydrogenase E1, alpha polypeptide	96035_at	0.6950	0.0112	Valine,_leucine_and_isoleucine_degradation
BPHL	biphenyl hydrolase-like (serine hydrolase, breast epithelial mucin-associated antigen)	162248_f_at	0.7975	0.0387	Cellular amino acid metabolic process
BPHL	biphenyl hydrolase-like (serine hydrolase, breast epithelial mucin-associated antigen)	96231_at	0.4919	0.0059	Cellular amino acid metabolic process
BRAP	BRCA1 associated protein	160113_at	0.7706	0.0187	Ras_signaling_pathway
BTBD1	BTB (POZ) domain containing 1	94878_at	0.7061	0.0086	Binds to topoisomerase I
C1RA	complement component 1, r subcomponent A	161571_f_at	0.6556	0.0079	Complement activation
C2CD2	C2 calcium-dependent domain containing 2	102862_at	0.7740	0.0158	Insulin binding
CA3	carbonic anhydrase 3	160375_at	0.1800	0.0409	Metabolism and Nitrogen metabolism
CAR5A	carbonic anhydrase 5a, mitochondrial	98137_at	0.5102	0.0298	Nitrogen_metabolism
CASP6	caspase 6	94458_at	0.7771	0.0421	Apoptosis
CASP9	caspase 9	103711_at	0.6943	0.0023	Apoptosis
CAT	catalase 1	160479_at	0.5279	0.0451	Glyoxylate_and_dicarboxylate_metabolism
CCDC94	coiled-coil domain containing 94	104502_f_at	0.7831	0.0057	
CCL27A	chemokine (C-C motif) ligand 27A	100972_s_at	0.7359	0.0379	Cell migration, chemotaxis
CCNI	cyclin I	94819_f_at	0.7367	0.0416	Regulation of cell cycle
CCNI	cyclin I	94820_r_at	0.6850	0.0498	Regulation of cell cycle
CD1D1	CD1d1 antigen	103422_at	0.5826	0.0487	Hematopoietic_cell_lineage
CD8B1	CD8 antigen, beta chain	94000_at	0.7187	0.0395	Immune system process
CDC42EP4	CDC42 Effector Protein (Rho GTPase Binding) 4	94036_at	0.7293	0.0065	ERK signaling, actin nucleation, ARP-WASP
CDIP1	cell death inducing Trp53 target 1	100587_f_at	0.7212	0.0392	Cell death
CDK8	cyclin-dependent kinase 8	96726_at	0.7910	0.0452	Signaling by GPCR and Disease
CEBPA	CCAAT/enhancer binding protein (C/EBP), alpha	98447_at	0.6203	0.0146	Transcriptional_regulation
CES3A	carboxylesterase 3A	101538_i_at	0.4904	0.0217	Hydrolysis or transesterification of xenobiotics
CES3A	carboxylesterase 3A	101539_f_at	0.3680	0.0110	Hydrolysis or transesterification of xenobiotics
CES3A	carboxylesterase 3A	99941_at	0.2373	0.0258	Hydrolysis or transesterification of xenobiotics
CFAP20	cilia and flagella associated protein 20	93588_at	0.6359	0.0451	poly(A) RNA binding
CIPC	CLOCK interacting protein, circadian	104298_at	0.6768	0.0018	regulation of transcription
CISD1	CDGSH iron sulfur domain 1	94526_at	0.6599	0.0176	regulation of cellular respiration
CLPB	ClpB caseinolytic peptidase B	99111_at	0.7919	0.0447	cellular response to heat
Cmbl	carboxymethylenebutenolidase-like (Pseudomonas)	96122_at	0.5927	0.0296	Metabolism
CMTM6	CKLF-like MARVEL transmembrane domain containing 6	160312_at	0.6632	0.0198	chemotaxis
CMTM8	CKLF-like MARVEL transmembrane domain containing 8	104626_at	0.7482	0.0077	chemotaxis
COASY	Coenzyme A synthase	94241_at	0.5742	0.0082	Pantothenate_and_CoA_biosynthesis
COL18A1	collagen, type XVIII, alpha 1	162483_f_at	0.7097	0.0104	Protein_digestion_and_absorption
COL18A1	collagen, type XVIII, alpha 1	99638_at	0.7356	0.0139	Protein_digestion_and_absorption
COMMD9	COMM domain containing 9	104302_f_at	0.6795	0.0328	transcription, DNA-templated, transport
COMT	catechol-O-methyltransferase	98535_at	0.5664	0.0478	Dopaminergic_synapse
COQ10A	coenzyme Q10A	95587_at	0.7607	0.0266	Mitochondrial respiration
COX4I1	cytochrome c oxidase, subunit IVa	102124_f_at	0.7220	0.0207	Oxidative_phosphorylation
COX6C	cytochrome c oxidase, subunit VIc	100550_f_at	0.6288	0.0196	Oxidative_phosphorylation
COX7A2	cytochrome c oxidase, subunit VIIa 3	93820_at	0.6308	0.0188	Oxidative_phosphorylation

COX7B	cytochrome c oxidase subunit VIIb	101580_at	0.5443	0.0203	Oxidative_phosphorylation
COX7C	cytochrome c oxidase, subunit VIIc	99660_f_at	0.7863	0.0098	Oxidative_phosphorylation
CPT2	carnitine palmitoyltransferase 2	95646_at	0.5520	0.0159	Fatty_acid_degradation
CRB3	crumbs family member 3	104584_f_at	0.7797	0.0122	Tight_junction
CRY1	cryptochrome 1 (photolyase-like)	94420_f_at	0.5822	0.0062	Circadian_rhythm
CRYZ	crystallin, zeta	98131_at	0.6877	0.0042	xenobiotic catabolic process
CSAD	deoxyribonuclease II alpha	99184_at	0.4862	0.0322	Taurine_and_hypotaurine_metabolism
CSTF2T	cleavage stimulation factor, 3' pre-RNA subunit 2, 64 kDa, tau	104126_at	0.6822	0.0387	mRNA_surveillance_pathway
CXCL12	stromal cell derived factor 1	100112_at	0.6010	0.0021	Axon_guidance, chemokine_signaling
CXCL12	stromal cell derived factor 1	160511_at	0.5600	0.0140	Axon_guidance, chemokine_signaling
CXXC5	CXXC finger 5	95701_at	0.5630	0.0374	signal transducer activity
CYB5A	cytochrome b5 type A (microsomal)	98533_at	0.6759	0.0033	Disease and Metabolism
CYB5B	cytochrome b5 type B	103619_at	0.6396	0.0167	Cytochrome P450 - arranged by substrate type
CYP1A2	cytochrome P450, 1a2, aromatic compound inducible	102998_at	0.4880	0.0438	Caffeine_metabolism
CYP2C29	cytochrome P450, 2c29	93585_at	0.6824	0.0207	Arachidonic_acid_metabolism
CYP3A25	cytochrome P450, 3a25	104024_at	0.5297	0.0044	Chemical_carcinogenesis
CYP4B1	cytochrome P450, subfamily IV B, polypeptide 1	162044_f_at	0.6918	0.0413	Metabolism
CYP4F13	Cyp4f13 cytochrome P450, family 4, subfamily f, polypeptide 13	104129_at	0.7225	0.0035	Arachidonic_acid_metabolism
CYP7B1	cytochrome P450, 7b1	161345_f_at	0.2495	0.0018	Primary_bile_acid_biosynthesis
CYP7B1	cytochrome P450, 7b1	92898_at	0.2128	0.0098	Primary_bile_acid_biosynthesis
CYP8B1	cytochrome P450, 8b1, sterol 12 alpha-hydrolase	103284_at	0.4282	0.0162	PPAR_signaling_pathway
D10JHU81E	DNA segment, Chr 10, Johns Hopkins University 81 expressed	101537_at	0.6468	0.0434	
DALRD3	DALR anticodon binding domain containing 3	160346_at	0.7693	0.0170	Arginine-tRNA ligase activity
DCTPP1	dCTP pyrophosphatase 1	96294_s_at	0.7909	0.0221	Nucleoside triphosphate catabolism
DDT	D-dopachrome tautomerase	100564_at	0.6016	0.0042	Dopachrome isomerase, D-dopachrome decarboxylase
DDX17	DEAD (Asp-Glu-Ala-Asp) box polypeptide 17	162424_f_at	0.6571	0.0424	GPCR Pathway
DEB1	differentially expressed in B16F10 1	95478_at	0.6225	0.0148	
DGCR2	DiGeorge syndrome critical region gene 2	161247_f_at	0.7501	0.0000	cell adhesion
DHFR	mouse dihydrofolate reductase gene	104547_at	0.7931	0.0259	Folate_biosynthesis
DHRS3	retinal short-chain dehydrogenase/reductase 1	102797_at	0.5479	0.0170	Retinol_metabolism
DHRS4	DNA segment, Chr 14, University of California at Los Angeles 2	96678_at	0.6098	0.0112	Retinol_metabolism, Peroxisome
DHX16	DEAD/H (Asp-Glu-Ala-Asp/His) box polypeptide 16	100559_at	0.7989	0.0489	Spliceosome
DICER1	DNA segment, Chr 12, ERATO Doi 7, expressed	160957_at	0.7086	0.0013	MicroRNAs_in_cancer
DIO1	deiodinase, iodothyronine, type I	95552_at	0.3775	0.0430	Thyroid_hormone_signaling_pathway
DMGDH	dimethylglycine dehydrogenase	104086_at	0.5034	0.0018	Glycine,_serine_and_threonine_metabolism
DNAJA3	DnaJ (Hsp40) homolog, subfamily A, member 3	97868_at	0.7467	0.0427	Viral_carcinogenesis
DYNLRB1	dynein light chain roadblock-type 1	94862_i_at	0.7951	0.0136	Membrane transport
E2F8	E2F transcription factor 8	103203_f_at	0.6759	0.0220	Transcription factor, transcription corepressor
ECH1	enoyl coenzyme A hydratase 1, peroxisomal	93754_at	0.5257	0.0364	Peroxisome
ECHS1	enoyl Coenzyme A hydratase, short chain, 1, mitochondrial	95426_at	0.6640	0.0062	Beta_Alanine_metabolism
ECI1	enoyl-Coenzyme A delta isomerase 1	98527_at	0.5575	0.0099	Dodecenoyl-CoA delta-isomerase activity
EEPD1	endonuclease/exonuclease/phosphatase family domain containing 1	160916_at	0.7494	0.0319	DNA repair
EGFR	epidermal growth factor receptor	101841_at	0.4814	0.0411	ErbB_signaling_pathway
EI24	etoposide induced 2.4 mRNA	99629_at	0.5920	0.0118	p53_signaling_pathway
ELL	Elongation Factor RNA Polymerase II	102996_at	0.7149	0.0151	Disease and Gene Expression
ELOVL3	elongation of very long chain fatty acids (FEN1/Elo2, SUR4/Elo3, yeast)-like 3	103469_at	0.4662	0.0008	Fatty_acid_elongation
Emc6	ER membrane protein complex subunit 6	160267_at	0.6139	0.0176	autophagosome assembly
EPHX2	epoxide hydrolase 2, cytoplasmic	93051_at	0.3991	0.0156	Arachidonic_acid_metabolism
ERBB3	erb-b2 receptor tyrosine kinase 3	96771_at	0.5912	0.0468	ErbB_signaling_pathway
ERO1LB	ERO1-like beta (S. cerevisiae)	103531_f_at	0.5649	0.0461	Protein_processing_in_endoplasmic_reticulum
ETHE1	ethylmalonic encephalopathy 1	95660_at	0.5627	0.0148	Sulfur_metabolism
FAH	fumarylacetoacetate hydrolase	98588_at	0.7564	0.0262	Tyrosine_metabolism
FAM107B	family with sequence similarity 107, member B	96640_at	0.6291	0.0249	sensory perception of sound
FAM134A	family with sequence similarity 134, member A	95401_at	0.7580	0.0388	sensory perception of sound

FAM136A	family with sequence similarity 136, member A	160955_at	0.6259	0.0249	sensory perception of sound
FBXO8	f-box protein 8	94917_at	0.7205	0.0492	Arf6 signaling events
FBXO9	f-box protein 9	98906_at	0.6188	0.0141	ubiquitin-protein transferase activity
FERMT2	fermitin family member 2	96774_at	0.5158	0.0106	ERK Signaling and Cell junction organization
FGGY	FGGY carbohydrate kinase domain containing	102783_at	0.7845	0.0145	Kinase and phosphotransferase activity
FOXA1	forkhead box A1	92697_at	0.5515	0.0163	FOXA1 transcription factor network
FOXA2	forkhead box A2	93950_at	0.5227	0.0005	Maturity_onset_diabetes_of_the_young
FOXQ1	HNF-3/forkhead homolog 1 like	92658_at	0.4664	0.0029	Double-stranded DNA binding
FPGS	folylpolyglutamyl synthetase	160850_at	0.5864	0.0032	Folate_biosynthesis
FXYD1	FXYD domain-containing ion transport regulator 1	93040_at	0.6747	0.0258	cAMP_signaling_pathway
GABARAPL1	gamma-aminobutyric acid (GABA(A)) receptor-associated protein-like 1	93011_at	0.6356	0.0147	GABAergic_synapse, FoxO_signaling_pathway
GALM	Galactose Mutarotase (Aldose 1-Epimerase)	96789_i_at	0.5211	0.0184	Galactose_metabolism
GALM	Galactose Mutarotase (Aldose 1-Epimerase)	96790_f_at	0.5661	0.0139	Galactose_metabolism
GALT	galactose-1-phosphate uridyl transferase	104616_g_at	0.6170	0.0320	Galactose_metabolism
GAS1	growth arrest specific 1	94813_at	0.5385	0.0111	Hedgehog_signaling_pathway
GAS2	growth arrest specific 2	94338_g_at	0.3985	0.0266	GPCR Pathway
GCDH	glutaryl-Coenzyme A dehydrogenase	160194_at	0.6743	0.0206	Fatty_acid_degradation
GCGR	glucagon receptor	103498_at	0.6603	0.0085	Neuroactive_ligand_receptor_interaction
GFM1	G elongation factor, mitochondrial 1	160226_at	0.7020	0.0087	GTP binding and translation elongation factor activity
GLO1	glyoxalase 1	93268_at	0.4633	0.0185	Pyruvate_metabolism
GLO1	glyoxalase 1	93269_at	0.4075	0.0012	Pyruvate_metabolism
GNA12	guanine nucleotide binding protein, alpha 12	97226_at	0.6510	0.0218	cGMP_PKG_signaling_pathway
GNE	UDP-N-acetylglucosamine-2-epimerase/N-acetylmannosamine kinase	97924_at	0.7898	0.0485	Amino_sugar_and_nucleotide_sugar_metabolism
GNPDA1	glucosamine-6-phosphate deaminase	100565_at	0.7696	0.0184	Amino_sugar_and_nucleotide_sugar_metabolism
GPHN	gephyrin	99441_at	0.4842	0.0085	GABAergic_synapse
GRB7	growth factor receptor bound protein 7	103095_at	0.7624	0.0476	PI-3K cascade and Signaling by FGFR
GSN	gelsolin	93750_at	0.7776	0.0478	Fc_gamma_R_mediated_phagocytosis
GSTA3	glutathione S-transferase, alpha 3	93015_at	0.3953	0.0140	Glutathione_metabolism
GSTK1	glutathione S-transferase kappa 1	96670_at	0.4952	0.0147	Glutathione_metabolism
GSTM6	glutathione S-transferase, mu 6	104636_at	0.7036	0.0061	Glutathione_metabolism
GSTO1	glutathione S-transferase omega 1	97819_at	0.6456	0.0418	Glutathione_metabolism
GUCD1	guanylyl cyclase domain containing 1	95119_at	0.6825	0.0194	
H2AFV	H2A histone family, member V	96710_at	0.5371	0.0225	Alcoholism
H2-KE6	H2-K region expressed gene 6	102991_s_at	0.6125	0.0291	metabolic process
HACD3	3-hydroxyacyl-CoA dehydratase 3	93747_at	0.7956	0.0401	lipid metabolic process
HAGH	hydroxyacyl glutathione hydrolase	100042_at	0.4620	0.0067	Pyruvate_metabolism
HDAC5	histone deacetylase 5	104376_at	0.7336	0.0429	Histone deacetylation
HES6	Hes Family BHLH Transcription Factor 6	97334_at	0.5705	0.0302	Notch signaling pathway
HHEX	hematopoietically expressed homeobox	98408_at	0.5139	0.0003	Maturity_onset_diabetes_of_the_young
HIBADH	3-hydroxyisobutyrate dehydrogenase	97279_at	0.4746	0.0115	Valine,_leucine_and_isoleucine_degradation
HINT2	histidine triad nucleotide binding protein 2	94365_at	0.6912	0.0424	nucleotide binding and hydrolase activity
HIST1H2BA	histone cluster 1, H2ba	93889_f_at	0.6270	0.0186	Core component of nucleosome
HIST1H2BC	histone cluster 1, H2bc	93833_s_at	0.4920	0.0247	Core component of nucleosome
HJURP	Holliday junction recognition protein	160682_at	0.7444	0.0305	Cell cycle
HMGCS2	3-hydroxy-3-methylglutaryl-Coenzyme A synthase 2	92590_at	0.4359	0.0057	Butanoate_metabolism
HNF1B	HNF1 homeobox B	101396_at	0.6919	0.0166	L1CAM interactions, beta-cell development
HSD17B4	hydroxysteroid 17-beta dehydrogenase 4	97515_at	0.5871	0.0476	Primary_bile_acid_biosynthesis
HSD3B2	hydroxysteroid dehydrogenase-2, delta<5>-3-beta	101659_at	0.4311	0.0027	Steroid_hormone_biosynthesis
HSD3B5	hydroxysteroid dehydrogenase-5, delta<5>-3-beta	94795_at	0.0759	0.0006	Steroid_hormone_biosynthesis
HSD3B6	hydroxysteroid dehydrogenase-6, delta<5>-3-beta	102729_f_at	0.4753	0.0129	Steroid_hormone_biosynthesis
HSD3B7	hydroxy-delta-5-steroid dehydrogenase, 3 beta- and steroid delta-isomerase 7	160104_at	0.6002	0.0100	Primary_bile_acid_biosynthesis
HSDL2	hydroxysteroid dehydrogenase like 2	96095_i_at	0.4913	0.0084	oxidoreductase activity and sterol binding
HSDL2	hydroxysteroid dehydrogenase like 2	96096_f_at	0.5761	0.0069	oxidoreductase activity and sterol binding
IGFALS	insulin-like growth factor binding protein, acid labile subunit	97987_at	0.5882	0.0135	IGF transport

IMMP1L	IMP1 inner mitochondrial membrane peptidase-like (S. cerevisiae)	96068_at	0.4722	0.0249	Protein_export
IMPA1	inositol (myo)-1(or 4)-monophosphatase 1	101498_at	0.7097	0.0019	Inositol_phosphate_metabolism
INHBC	inhibin beta-C	103986_at	0.7720	0.0213	Cytokine_cytokine_receptor_interaction
IPO8	importin 8	104163_at	0.6865	0.0194	Ran GTPase binding
ITM2B	integral membrane protein 2B	101123_at	0.7203	0.0305	Disease and RNA Polymerase I Promoter Opening
IVD	isovaleryl coenzyme A dehydrogenase	104153_at	0.6673	0.0115	Valine,_leucine_and_isoleucine_degradation
LYD	iodotyrosine deiodinase	103714_at	0.6111	0.0461	Metabolism of amino acids and derivatives
KCTD20	potassium channel tetramerisation domain containing 20	99940_at	0.7223	0.0117	positive regulation of phosphorylation
KDELR2	KDEL (Lys-Asp-Glu-Leu) Endoplasmic Reticulum Protein Retention	101922_at	0.6952	0.0454	Activation of cAMP-Dependent PKA
KEG1	kidney expressed gene 1	96938_at	0.2884	0.0067	transferase activity
KLKB1	kallikrein B, plasma 1	104344_at	0.4446	0.0094	Complement_and_coagulation_cascades
KPNA6	karyopherin (importin) alpha 6	160624_at	0.7501	0.0028	Interferon Signaling
LAMP2	lysosomal membrane glycoprotein 2	100136_at	0.6116	0.0266	Lysosome
LAP3	leucine aminopeptidase 3	98112_r_at	0.5592	0.0127	Arginine_and_proline_metabolism
LDAH	lipid droplet associated hydrolase	94382_at	0.5609	0.0113	phospholipase, prenylcysteine methylesterase, lipase
LIAS	lipoic acid synthetase	98909_at	0.7216	0.0003	Lipoic_acid_metabolism
LIPC	lipase, hepatic	98962_at	0.4264	0.0043	Glycerolipid_metabolism
LONP2	lon peptidase 2, peroxisomal	96890_at	0.5234	0.0493	Glycosylatin
LPCAT3	lysophosphatidylcholine acyltransferase 3	98965_at	0.7236	0.0049	Metabolism
LPPOS	LIM domain containing preferred translocation partner in lipoma, opposite strand	92941_at	0.7577	0.0210	
LRP1	low density lipoprotein receptor-related protein 1	101073_at	0.7826	0.0451	Alzheimer's_disease
LSM1	LSM1 homolog, mRNA degradation associated	96946_at	0.7993	0.0109	RNA_degradation
LSM14B	LSM family member 14B	103449_at	0.7269	0.0297	regulation of translation
LTBR	lymphotoxin B receptor	97980_at	0.7881	0.0199	Cytokine_cytokine_receptor_interaction
MAN1A	mannosidase 1, alpha	160580_at	0.6993	0.0158	N_Glycan_biosynthesis
MASP1	mannan-binding lectin serine protease 1	102284_at	0.6176	0.0431	Complement_and_coagulation_cascades
MBL1	mannose binding lectin, liver (A)	101474_at	0.4630	0.0110	Complement_and_coagulation_cascades
MCCC1	methylcrotonoyl-Coenzyme A carboxylase 1 (alpha)	94940_at	0.6747	0.0136	Valine,_leucine_and_isoleucine_degradation
MCEE	methylmalonyl CoA epimerase	102022_at	0.5565	0.0146	Glyoxylate_and_dicarboxylate_metabolism
MCM10	minichromosome maintenance deficient 10 (S. cerevisiae)	103553_at	0.4215	0.0036	Cell Cycle, Mitotic
MECOM	MDS1 and EVI1 complex locus	100754_at	0.7915	0.0115	MAPK signaling pathway and Pathways in cancer
METTL20	methyltransferase like 20	102233_at	0.6341	0.0118	protein methyltransferase activity
MFHAS1	malignant fibrous histiocytoma amplified sequence 1	93195_at	0.7481	0.0343	small GTPase mediated signal transduction
MFN1	mitofusin 1	95594_at	0.7733	0.0312	GTP binding and GTPase activity
MLYCD	malonyl-CoA decarboxylase	103622_at	0.7920	0.0105	AMPK_signaling_pathway
MPDZ	multiple PDZ domain protein	93887_at	0.7860	0.0144	Tight_junction
MRPL15	mitochondrial ribosomal protein L15	96296_at	0.7473	0.0207	Ribosome
MRPL34	mitochondrial ribosomal protein L34	160345_at	0.6721	0.0271	Ribosome
MRPL39	mitochondrial ribosomal protein L39	93062_at	0.6268	0.0428	mitochondrial genome maintenance
MRPL43	mitochondrial ribosomal protein L43	99674_at	0.7305	0.0229	translation
MSRB1	methionine sulfoxide reductase B1	160275_at	0.5891	0.0099	Selenium Metabolism and Selenoproteins
MTERFD1	mitochondrial transcription termination factor 3	160425_at	0.7620	0.0473	regulation of transcription, DNA-templated
MTHFR	5,10-methylenetetrahydrofolate reductase	102360_at	0.7668	0.0215	One_carbon_pool_by_folate
MTIF2	mitochondrial translational initiation factor 2	93859_at	0.6974	0.0149	TNF-alpha/NF-kB Signaling Pathway
MUG-PS1	murinoglobulin, pseudogene 1	92837_f_at	0.6589	0.0407	
MUP1	major urinary protein 1	102096_f_at	0.3336	0.0326	pheromone activity
NAA60	N(alpha)-acetyltransferase 60, NatF catalytic subunit	160195_at	0.6445	0.0201	Alpha-N-acetyltransferase, H4 acetyltransferase
NACC2	nucleus accumbens associated 2, BEN and BTB (POZ) domain containing	92526_f_at	0.6707	0.0136	Transcription
NAT2	N-acetyl transferase 2	92746_at	0.7273	0.0083	Caffeine_metabolism
NAT8	N-acetyltransferase 8 (GCN5-related)	92835_at	0.4445	0.0053	Sphingolipid metabolism
NCAPH	non-SMC condensin I complex, subunit H	161172_f_at	0.7862	0.0176	Cell Cycle, Mitotic
NCAPH2	non-SMC condensin II complex, subunit H2	160292_at	0.7499	0.0336	Cell Cycle, Mitotic
NDE1	nuclear distribution gene E homolog 1 (A nidulans)	94910_at	0.6146	0.0396	Cell Cycle, Mitotic
NDFIP1	Nedd4 family interacting protein 1	93025_at	0.6087	0.0429	signal transducer activity and WW domain binding

NDRG2	N-myc downstream regulated 2	161610_at	0.4207	0.0028	Angiogenesis (CST) and Apoptosis and Autophagy
NDRG2	N-myc downstream regulated 2	96088_at	0.4946	0.0118	Angiogenesis (CST) and Apoptosis and Autophagy
NDST1	N-deacetylase/N-sulfotransferase (heparan glucosaminyl) 1	93590_at	0.6089	0.0238	Glycosaminoglycan_heparan_sulfate_heparin
NDUFAB1	NADH dehydrogenase (ubiquinone) 1, alpha/beta subcomplex, 1	96909_at	0.5972	0.0361	Oxidative_phosphorylation
NDUFAF1	NADH dehydrogenase (ubiquinone) 1 alpha subcomplex, assembly factor 1	100892_at	0.7204	0.0110	unfolded protein binding
NDUFB11	NADH dehydrogenase (ubiquinone) 1 beta subcomplex, 11	96887_at	0.5715	0.0249	Oxidative_phosphorylation
NDUFB3	NADH dehydrogenase (ubiquinone) 1 beta subcomplex 3	93562_at	0.6858	0.0104	Oxidative_phosphorylation
NDUFB5	NADH dehydrogenase (ubiquinone) 1 beta subcomplex 5	97307_f_at	0.7186	0.0384	Oxidative_phosphorylation
NDUFB9	NADH dehydrogenase (ubiquinone) 1 beta subcomplex, 9	100079_at	0.5512	0.0419	Oxidative_phosphorylation
NDUFS1	NADH dehydrogenase (ubiquinone) Fe-S protein 1	93572_at	0.6780	0.0076	Oxidative_phosphorylation
NDUFS2	NADH dehydrogenase (ubiquinone) Fe-S protein 2	93972_at	0.5839	0.0300	Oxidative_phosphorylation
NDUFS6	NADH dehydrogenase (ubiquinone) Fe-S protein 6	96291_f_at	0.6980	0.0469	Oxidative_phosphorylation
NFIX	nuclear factor I/X	101930_at	0.5655	0.0003	Transcription
NGRN	neugrin	103875_at	0.5673	0.0143	Nervous system development
NIT2	nitrilase family, member 2	160135_at	0.6279	0.0116	Alanine,_aspartate_and_glutamate_metabolism
NME3	NME/NM23 nucleoside diphosphate kinase 3	94981_i_at	0.6567	0.0363	Purine_metabolism, Pyrimidine_metabolism
NME3	NME/NM23 nucleoside diphosphate kinase 3	94982_f_at	0.5612	0.0426	Purine_metabolism, Pyrimidine_metabolism
NOP10	NOP10 ribonucleoprotein	97250_at	0.6531	0.0443	Ribosome biogenesis in eukaryotes
NPC1	Niemann Pick type C1	98114_at	0.7936	0.0388	Lysosome
NR1H4	nuclear receptor subfamily 1, group H, member 4	97969_at	0.6524	0.0159	Bile_secretion
NSMCE4A	non-SMC element 4 homolog A (S. cerevisiae)	93986_at	0.5714	0.0389	DNA damage
ORMDL3	ORM1-like 3 (S. cerevisiae)	98065_at	0.7437	0.0394	Ceramide metabism, sphingolipid homeostasis
OSBPL11	oxysterol binding protein-like 11	94439_at	0.6251	0.0329	Phospholipid binding
OSBPL1A	oxysterol binding protein-like 1A	93316_at	0.6539	0.0499	Lipid transport
OTC	ornithine transcarbamylase	94414_at	0.5284	0.0260	Arginine_and_proline_metabolism
PAH	phenylalanine hydroxylase	95407_at	0.5724	0.0371	Phenylalanine,_tyrosine_and_tryptophan_biosynthesis
PCBD2	pterin 4 alpha carbinolamine dehydratase/dimerization cofactor of hepatocyte nuclear fact	104237_at	0.6759	0.0331	phenylalanine degradation I
PCMT1	protein-L-isoaspartate (D-aspartate) O-methyltransferase 1	94347_i_at	0.7248	0.0001	O-methyltransferase
PCYT2	Phosphate Cytidylyltransferase 2, Ethanolamine	103914_at	0.7438	0.0145	Glycerophospholipid_metabolism
PDE9A	phosphodiesterase 9A	102338_at	0.5803	0.0083	Purine_metabolism
PDZK1	PDZ domain containing 1	97288_at	0.5720	0.0301	Regulation of CFTR activity (norm and CF)
PEX13	peroxisomal biogenesis factor 13	94491_at	0.7598	0.0364	Peroxisome
PEX5	peroxin 5	104318_at	0.6694	0.0060	Peroxisome
PEX6	peroxisomal biogenesis factor 6	99469_at	0.6840	0.0370	Peroxisome
PGRMC2	progesterone receptor membrane component 2	94801_at	0.7487	0.0162	heme binding and steroid binding
PHYH	phytanoyl-CoA hydroxylase	96608_at	0.5183	0.0245	Peroxisome
PIK3C3	calsyntenin 1	104699_at	0.6621	0.0019	Inositol_phosphate_metabolism
PIPOX	peroxisomal sarcosine oxidase	101844_at	0.5879	0.0012	Glycine,_serine_and_threonine_metabolism
PJA1	praja ring finger 1, E3 ubiquitin protein ligase	101461_f_at	0.6384	0.0220	Class I MHC mediated Ag processing_presentation
PKP4	plakophilin 4	96187_at	0.6192	0.0373	Cell adhesion_Endothelial cell contacts
POLE4	polymerase (DNA-directed), epsilon 4 (p12 subunit)	95121_at	0.6754	0.0159	Base_excision_repair
POLR3K	polymerase (RNA) III (DNA directed) polypeptide K	104304_r_at	0.7715	0.0304	Cytosolic_DNA_sensing_pathway
PON1	paraoxonase 1	96895_at	0.3819	0.0278	Metabolism and Phase I, non P450
PPDPF	pancreatic progenitor cell differentiation and proliferation factor	93838_at	0.7812	0.0003	Cell fate, multicellular organismal development
PPP1R15B	protein phosphatase 1, regulatory (inhibitor) subunit 15b	95609_at	0.6652	0.0400	protein serine/threonine phosphatase activity
PPP2CA	protein phosphatase 2a, catalytic subunit, alpha isoform	92638_at	0.7850	0.0398	Adrenergic_signaling_in_cardiomyocytes
PPP6C	Protein Phosphatase 6, Catalytic Subunit	160821_r_at	0.7748	0.0112	protein serine/threonine phosphatase activity
PRADC1	protease-associated domain containing 1	95619_at	0.6975	0.0152	
PRODH2	proline oxidase 1	103452_at	0.7139	0.0454	Arginine_and_proline_metabolism
PROSC	proline synthetase co-transcribed	101568_at	0.6637	0.0090	pyridoxal phosphate binding
PSEN2	presenilin 2	99930_s_at	0.5262	0.0437	Notch_signaling_pathway
PSMD11	proteasome (prosome, macropain) 26S subunit, non-ATPase, 11	160305_at	0.6523	0.0300	Epstein_Barr_virus_infection
PSMD12	proteasome (prosome, macropain) 26S subunit, non-ATPase, 12	93971_f_at	0.7219	0.0262	Epstein_Barr_virus_infection
PTDSS1	phosphatidylserine synthase 1	101931_at	0.6253	0.0367	Glycerophospholipid_metabolism

PTPRD	protein tyrosine phosphatase, receptor type, D	93485_at	0.5825	0.0006	Receptor protein tyrosine phosphatase activity
PTS	6-pyruvoyl-tetrahydropterin synthase	160844_at	0.7869	0.0078	Folate_biosynthesis
PXMP2	peroxisomal membrane protein 2, 22 kDa	104098_at	0.7532	0.0127	Peroxisome
QDPR	quinoid dihydropteridine reductase	96948_at	0.6497	0.0487	Folate_biosynthesis
RAD23B	RAD23b homolog (S. cerevisiae)	96102_i_at	0.7459	0.0229	Nucleotide_excision_repair
RARS2	arginyl-tRNA synthetase 2, mitochondrial	104305_at	0.7396	0.0244	translation
RBBP4	retinoblastoma binding protein 4	92647_at	0.5233	0.0216	Cell Cycle, Mitotic
RBBP9	retinoblastoma binding protein 9	104618_at	0.7300	0.0361	hydrolase activity
RBP4	retinol binding protein 4, plasma	96047_at	0.7816	0.0346	Signaling by GPCR and Disease
RDH7	retinol dehydrogenase 7	100634_at	0.7132	0.0097	Metabolic process, oxidation-reduction process
REG1	regenerating islet-derived 1	162312_f_at	0.7385	0.0285	Negative regulation of cell proliferation
RNF8	ring finger protein 8	99028_at	0.7898	0.0186	UPS-dependent proteolysis, SMAD signaling
RPP25L	ribonuclease P/MRP 25 subunit-like	102194_at	0.7498	0.0050	nucleic acid binding
RREB1	Ras Responsive Element Binding Protein 1	160824_at	0.5778	0.0063	DNA binding transcription factor activity
RTFDC1	replication termination factor 2 domain containing 1	95501_at	0.7313	0.0180	
S100A1	S100 calcium binding protein A1	95453_f_at	0.5064	0.0188	Ca, cAMP and Lipid Signaling
S100A13	S100 calcium binding protein A13	161121_f_at	0.6865	0.0305	calcium ion binding and lipid binding
S1PR1	Sphingosine-1-Phosphate Receptor 1	161788_f_at	0.7089	0.0027	Signaling by GPCR and NF-KappaB Family Pathway
SAR1B	SAR1 gene homolog B (S. cerevisiae)	94870_f_at	0.5861	0.0175	Protein_processing_in_endoplasmic_reticulum
SAR1B	SAR1 gene homolog B (S. cerevisiae)	94871_r_at	0.5905	0.0230	Protein_processing_in_endoplasmic_reticulum
SARDH	sarcosine dehydrogenase	96763_at	0.4726	0.0003	Glycine,_serine_and_threonine_metabolism
SDHA	succinate dehydrogenase complex, subunit A, flavoprotein (Fp)	94080_at	0.4666	0.0237	Oxidative_phosphorylation
SDHB	succinate dehydrogenase complex, subunit B, iron sulfur (Ip)	95053_s_at	0.6625	0.0359	Oxidative_phosphorylation
SEC14L2	SEC14-Like 2	96758_s_at	0.5493	0.0043	Phospholipid binding and vitamin E binding
SEC14L2	SEC14-Like 2	96759_r_at	0.6252	0.0047	Phospholipid binding and vitamin E binding
SEL1L	sel-1 suppressor of lin-12-like (C. elegans)	92870_at	0.6521	0.0078	Notch signaling pathway, ERAD pathway
SELENBP1	selenium binding protein 1	100596_at	0.5805	0.0147	transport, brown fat cell differentiation
SEPHS2	selenophosphate synthetase 2	93557_at	0.5030	0.0037	Selenocompound_metabolism
SERPINE2	serine (or cysteine) peptidase inhibitor, clade E, member 2	97487_at	0.4846	0.0017	Insulin regulation of translation
SFXN1	sideroflexin 1	92831_at	0.5840	0.0074	cation transmembrane transporter activity
SIRT3	sirtuin 3 (silent mating type information regulation 2, homolog) 3 (S. cerevisiae)	160869_at	0.6589	0.0070	Central_carbon_metabolism_in_cancer
SKP1A	transcription elongation factor B (SIII), polypeptide 1 (15 kDa),-like	99607_at	0.6424	0.0017	Cell_cycle
SLC16A2	solute carrier family 16, member 2	162219_f_at	0.6057	0.0052	Thyroid homone transporter
SLC16A2	solute carrier family 16, member 2	100491_at	0.6162	0.0136	Thyroid_hormone_signaling_pathway
SLC17A1	solute carrier family 17 (sodium/hydrogen exchanger), member 1	96078_g_at	0.7019	0.0095	Transport of glucose, bile salts, organic acids
SLC22A30	solute carrier family 22, member 30	103703_f_at	0.4284	0.0058	sodium-independent organic anion transport
SLC25A15	solute carrier family 25 (mitochondrial carrier; ornithine transporter), member 15	104007_at	0.6849	0.0185	Cell Cycle, Mitotic
SLC25A44	solute carrier family 25, member 44	160392_at	0.7584	0.0487	translation, transport
SLC27A2	solute carrier family 27 (fatty acid transporter), member 2	100967_at	0.4794	0.0031	PPAR_signaling_pathway, peroxisome
SLC2A1	solute carrier family 3, member 1	93304_at	0.7939	0.0318	Facilitated glucose transporter
SLC2A2	solute carrier family 2 (facilitated glucose transporter), member 2	103357_at	0.4505	0.0356	Carbohydrate_digestion_and_absorption
SMIM11	small integral membrane protein 11	104076_at	0.7441	0.0031	
SNRPD3	small nuclear ribonucleoprotein D3	98077_at	0.6045	0.0115	Spliceosome
SPIN1	spindlin 1	99563_at	0.6988	0.0237	methylated histone binding
SPPL3	signal peptide peptidase 3	98908_at	0.7497	0.0379	Aspartic endopeptidase activity
SRSF4	serine/arginine-rich splicing factor 4	104754_at	0.7991	0.0012	Gene Expression and Influenza A
SRXN1	sulfiredoxin 1 homolog (S. cerevisiae)	161090_i_at	0.7362	0.0173	Oxidoreductase activity
STOML2	Stomatin (EPB72)-Like 2	96289_at	0.7974	0.0208	receptor binding and cardiolipin binding
SUDS3	suppressor of defective silencing 3 homolog (S. cerevisiae)	98070_at	0.7985	0.0064	Notch1 pathway for NF-KB activity modulation
SURF1	Surfeit 1	101031_at	0.7428	0.0394	Respiratory electron transport
SYF2	SYF2 homolog, RNA splicing factor (S. cerevisiae)	96341_at	0.7972	0.0142	Spliceosome
Tada1	transcriptional adaptor 1	104740_at	0.6717	0.0032	transcription coactivator, histone acetyltransferase
TARS	threonyl-tRNA synthetase	95054_at	0.5808	0.0304	Aminoacyl_tRNA_biosynthesis
TBX3	T-box 3	103538_at	0.6190	0.0141	Signaling_pluripotency_of_stem_cells

TCERG1	TATA box binding protein (TBP)-associated factor, RNA polymerase II, S, 150kD	101008_at	0.7801	0.0439	Spliceosome
TFPI2	tissue factor pathway inhibitor 2	94383_at	0.5698	0.0435	Clotting cascade, cell adhesion_plasmin signaling
TFR2	transferrin receptor 2	160674_at	0.6773	0.0079	AMPK pathway and Iron metabolism in placenta
TGDS	TDP-glucose 4,6-dehydratase	103635_at	0.7817	0.0226	Metabolism
TGOLN1	trans-golgi network protein	99144_s_at	0.7038	0.0170	Golgi to endosome transport
TGOLN1	trans-golgi network protein 2	99143_at	0.6376	0.0415	Golgi to endosome transport
TIMD2	T cell immunoglobulin and mucin domain containing 2	97335_at	0.6164	0.0322	
TIMM9	Translocase Of Inner Mitochondrial Membrane 9 Homolog (Yeast)	96760_at	0.6716	0.0007	Biosynthesis of the N-glycan precursor
TMEM14C	transmembrane protein 14C	96353_at	0.5901	0.0401	Heme biosynthetic, RBC differentiation
TMEM183A	transmembrane protein 183A	93301_at	0.7540	0.0258	
TMEM30B	transmembrane protein 30B	96749_f_at	0.7414	0.0194	transport
TOLLIP	TOLLIP protein	160809_at	0.7694	0.0436	Toll_like_receptor_signaling_pathway
TOM1L1	target of myb1-like 1 (chicken)	104063_at	0.6088	0.0119	SH3 binding and protein kinase activator activity
TRP53INP2	transformation related protein 53 inducible nuclear protein 2	160376_at	0.5293	0.0021	Transcription regulation
TTC30B	tetratricopeptide repeat domain 30B	160908_r_at	0.7184	0.0199	cell projection organization, intraciliary transport
TXNRD2	thioredoxin reductase 2	160437_at	0.7593	0.0046	Pyrimidine_metabolism
UBXN1	UBX domain protein 1	97239_at	0.7561	0.0221	ubiquitin protein ligase binding and ATPase binding
UQCRB	ubiquinol-cytochrome c reductase binding protein	95472_f_at	0.6067	0.0478	Oxidative_phosphorylation
UQCRC2	ubiquinol cytochrome c reductase core protein 2	102000_f_at	0.5602	0.0059	Oxidative_phosphorylation
USMG5	upregulated during skeletal muscle growth 5	95718_f_at	0.6143	0.0214	Maintaining ATP synthase in mitochondria.
USP4	ubiquitin specific protease 4 (proto-oncogene)	99064_at	0.7599	0.0018	Cell Cycle / Checkpoint Control and Protein Stability
VAPB	vesicle-associated membrane protein, associated protein B and C	104501_at	0.7426	0.0009	Sphingolipid metabolism and Metabolism
WDR45	WD repeat domain 45	96728_at	0.7697	0.0414	PI-3-phosphate and PI-3,5-bisphosphate binding
WDR45B	WE repeat domain 45B	96264_at	0.7817	0.0157	PI-3-phosphate and PI-3,5-bisphosphate binding
WDR48	WE Repeat domain 48	96654_at	0.7754	0.0009	Activator of deubiquitinase USP1, USP12 and USP46
WRNIP1	Werner helicase interacting protein 1	103415_at	0.4734	0.0061	identical protein binding and ATPase activity
YAP1	yes-associated protein, 65 kDa	161308_f_at	0.7855	0.0065	Hippo_signaling_pathway
ZDHHC14	zinc finger, DHHC domain containing 14	161782_r_at	0.6918	0.0425	Protein palmitoylation
ZMYND11	zinc finger, MYND domain containing 11	97484_at	0.6810	0.0204	Transcription regulation

Microarray gene analysis was conducted as described in the Method section. The probes used to study individual genes are listed along with the gene symbols and gene names. Some genes may have more than one probe. FC stands for fold of change over control (non-DEN treated). P values refer to the significance test. Genes listed in this table have FC of <0.80 with a *p* value <0.05. The function of the genes were obtained via multiple bioinformatics sources. Only main functions are listed. Not all genes have a clearly defined function.

Genes shown in red font are involved in amino acids metabolism, and are further detailed in S11 Table. Not all genes related to metabolism are highlighted.
