# Supplementary material for: Gene Expression Analysis Indicates Divergent Mechanisms in DEN-Induced Carcinogenesis in Wild Type and Bid-Deficient Livers
Source: PLoS One. 2016 May 19;11(5):e0155211. doi: 10.1371/journal.pone.0155211 (PMC4873180; doi:10.1371/journal.pone.0155211)
Supplement: S5 Table — (PDF) [file pone.0155211.s005.pdf]

S5 Table. Up-regulated genes in livers of Bid-deficient mice treated with DEN for 4-6 months

Genes Symbol	Gene Name	Probes	FC	p value	Function
ACTB	actin, beta, cytoplasmic	101578_f_at	1.4941	0.0426	Adherens_junction
ALAS1	aminolevulinic acid synthase 1	93500_at	2.0168	0.0376	Glycine,_serine_and_threonine_metabolism
APCDD1	adenomatosis polyposis coli down-regulated 1	96132_at	1.2651	0.0084	Wnt-protein binding
ATP5D	ATP synthase, H+ transporting, mitochondrial F1 complex, delta subunit	161047_at	1.2512	0.0091	Oxidative_phosphorylation
ATP6V0A2	ATPase, H+ Transporting, Lysosomal V0 Subunit A2	102273_at	1.2758	0.0145	Collecting_duct_acid_secretion
B3GALT2	UDP-Gal:betaGlcNAc beta 1,3-galactosyltransferase, polypeptide 2	92341_at	1.3625	0.0007	Glycosphingolipid_biosynthesis_lacto_and_neolacto_series
BAG3	Bcl2-associated athanogene 3	161980_f_at	1.5291	0.0253	Apoptosis
CBX3	chromobox homolog 3	100405_at	1.3720	0.0235	Gene Expression and Chromatin Regulation / Acetylation
CDKN1A	cyclin-dependent kinase inhibitor 1A (P21)	94881_at	1.3300	0.0182	Bladder_cancer
CLPS	colipase, pancreatic	160132_at	1.3634	0.0313	Fat_digestion_and_absorption
CYP2B9	cytochrome P450, 2b9, phenobarbitol inducible, type a	101862_at	3.2242	0.0265	Arachidonic_acid_metabolism
DMBT1	crp-ductin	99479_at	1.6017	0.0217	Salivary_secretion
DNAJC3	DnaJ (Hsp40) homolog, subfamily C, member 3	102415_r_at	1.4056	0.0270	Protein_processing_in_endoplasmic_reticulum
ERCC2	excision repair cross-complementing rodent repair deficiency, complementation group 2	161572_r_at	1.2842	0.0091	Cell Cycle / Checkpoint Control
GCK	glucokinase activity	102651_at	1.4118	0.0490	Amino_sugar_and_nucleotide_sugar_metabolism
GNAS	neuroendocrine secretory protein-like	102082_at	1.2871	0.0329	Adrenergic_signaling_in_cardiomyocytes
HSPA4	heat shock protein 4	100352_at	1.3709	0.0052	Antigen_processing_and_presentation
IGKV10-96	immunoglobulin kappa variable 10-96	102155_f_at	1.2502	0.0038	Immune response
ITGB7	integrin beta 7	162193_f_at	1.3135	0.0098	Focal_adhesion
MARK3	MAP/microtubule affinity-regulating kinase 3	161744_f_at	1.2813	0.0427	Cytoskeletal Signaling and TGF-beta Signaling Pathway
MOBP	myelin-associated oligodendrocytic basic protein	100536_at	1.2711	0.0478	Rab GTPase binding, structural constituent of myelin sheath
NDC80	NDC80 homolog, kinetochore complex component (S. cerevisiae)	161017_at	1.2674	0.0195	Structural constituent of cytoskeleton, protein binding
NDUFB2	NADH dehydrogenase (ubiquinone) 1 beta subcomplex, 2	162473_r_at	1.4321	0.0108	Oxidative_phosphorylation
OLFR1507	olfactory receptor 1507	96981_at	1.2804	0.0263	Olfactory receptor activity
OMP	olfactory marker protein	99407_at	1.2849	0.0177	Signal transducer activity
ONECUT1	one cut domain, family member 1	100737_at	1.3674	0.0098	Signaling_pathways_regulating_pluripotency_of_stem_cells
PDGFRB	platelet derived growth factor receptor, beta polypeptide	160867_at	1.2529	0.0291	Calcium_signaling_pathway
PTPN22	protein tyrosine phosphatase, non-receptor type 8	92356_at	1.2730	0.0243	PAK Pathway and CD28 co-stimulation
PTPRN2	protein tyrosine phosphatase, receptor-type, N polypeptide 2	94708_at	1.2708	0.0020	Type_I_diabetes_mellitus
REG1	regenerating islet-derived 1	160213_at	1.5265	0.0446	Growth factor activity, binding to phosphatase and carbohydrate
SCX	scleraxis	161030_at	1.2538	0.0237	DNA binding, protein binding
SPSB1	splA/ryanodine receptor domain and SOCS box containing 1	161013_f_at	1.3559	0.0346	Class I MHC mediated antigen processing and presentation
TRAV8	T-cell receptor alpha, variable 8	93223_at	1.2541	0.0326	Immune response
TSSK2	testis-specific serine kinase 2	102846_at	1.2610	0.0123	Sweet taste signaling. Transcription_CREM signaling
VNN1	vanin 1	104165_at	1.3957	0.0144	Pantothenate_and_CoA_biosynthesis
ZFHX4	zinc finger homeodomain 4	98760_at	1.2520	0.0136	DNA binding transcription factor activity

Microarray gene analysis was conducted as described in the Method section. The probes used to study individual genes are listed along with the gene symbols and gene names. Some genes may have more than one probe. FC stands for fold of change over control (non-DEN treated). P values refer to the significance test. Genes listed in this table have FC of >1.25 with a *p* value <0.05. The function of the genes were obtained via multiple bioinformatics sources. Only main functions are listed. Not all genes have a clearly defined function.
