# Supplementary material for: Gene Expression Analysis Indicates Divergent Mechanisms in DEN-Induced Carcinogenesis in Wild Type and Bid-Deficient Livers
Source: PLoS One. 2016 May 19;11(5):e0155211. doi: 10.1371/journal.pone.0155211 (PMC4873180; doi:10.1371/journal.pone.0155211)
Supplement: S6 Table — (PDF) [file pone.0155211.s006.pdf]

S6 Table. Down-regulated genes in livers of Bid-deficietn mice treated with DEN for 4-6 months

Genes Symbol	Gene Name	Probes	FC	p value	Function
ADCY6	adenylate cyclase 6	102321_at	0.7786	0.0178	Adrenergic_signaling_in_cardiomyocytes
AGXT	alanine-glyoxylate aminotransferase	93625_at	0.7602	0.0250	Alanine,_aspartate_and_glutamate_metabolism
AK4	adenylate kinase 4	99959_at	0.7386	0.0482	Hemostasis and Metabolism
AK4	adenylate kinase 4	99521_at	0.7948	0.0317	Hemostasis and Metabolism
AMACR	alpha-methylacyl-CoA racemase	95588_at	0.7749	0.0476	Primary_bile_acid_biosynthesis, Peroxisome
AMOT	angiomotin	95531_at	0.7640	0.0026	Hippo_signaling_pathway
ANKRD17	ankyrin repeat domain 17	104683_at	0.7847	0.0463	RNA binding
ATP5J	ATP synthase, H+ transporting, mitochondrial F0 complex subunit F	98128_at	0.7875	0.0161	Oxidative_phosphorylation
BCL6	B-cell leukemia/lymphoma 6	103015_at	0.4921	0.0074	FoxO_signaling_pathway
BTG1	B-cell translocation gene 1, anti-proliferative	93104_at	0.7857	0.0025	RNA_degradation
CBX4	chromobox 4	93697_at	0.6328	0.0069	Cellular Senescence
CFAP20	cilia and flagella associated protein 20	93588_at	0.6926	0.0012	Multicellular organismal development
CKS2	CDC28 protein kinase regulatory subunit 2	97527_at	0.6478	0.0082	Small_cell_lung_cancer
CLDN3	claudin 3	94493_at	0.7240	0.0107	Cell_adhesion_molecules_(CAMs)
CLTC	hemolytic complement	103458_at	0.7985	0.0051	Bacterial_invasion_of_epithelial_cells
COMMD4	COMM domain containing 4	99167_at	0.7743	0.0455	Transcription, DNA-templated
CTSZ	cathepsin Z	92633_at	0.7856	0.0262	Lysosome
CYB5R1	cytochrome b5 reductase 1	103922_f_at	0.7720	0.0174	Amino_sugar_and_nucleotide_sugar_metabolism
DBP	D site albumin promoter binding protein	160841_at	0.5924	0.0318	Circadian Clock
DCT	dopachrome tautomerase	103597_at	0.6239	0.0205	Tyrosine_metabolism, Melanogenesis
DCTN2	dynactin 2	94300_f_at	0.7557	0.0049	Vasopressin_regulated_water_reabsorption
DSC2	desmocollin 2	103506_f_at	0.6235	0.0135	Arrhythmogenic_right_ventricular_cardiomyopathy_(ARVC)
EFNA1	ephrin A1	103007_at	0.7789	0.0287	PI3K_Akt_, Ras_, Rap1_signaling_pathway
EGFR	epidermal growth factor receptor	101841_at	0.7391	0.0235	ErbB_signaling_pathway
ERBB3	erb-b2 receptor tyrosine kinase 3	96771_at	0.6169	0.0199	ErbB_signaling_pathway
ERDR1	erythroid differentiation regulator 1	98525_f_at	0.4556	0.0309	Regulation of cell proliferation, and cell migration
FBXO21	F-box protein 21	104109_at	0.6821	0.0170	Ubiquitin protein ligase complex
FERMT2	fermitin family member 2	96774_at	0.7684	0.0260	ERK Signaling and Cell junction organization
FLCN	folliculin	160242_at	0.6669	0.0106	Renal_cell_carcinoma
GNB2L1	guanine nucleotide binding protein, beta 2, related sequence 1	99340_at	0.7869	0.0222	ERK signaling, GPCR signaling
GRPEL2	GrpE-like 2, mitochondrial	102761_at	0.7790	0.0319	Biosynthesis of the N-glycan precursor
HEBP1	heme binding protein 1	103085_at	0.7317	0.0222	Signaling by GPCR
HINT1	histidine triad nucleotide binding protein 1	99581_at	0.7270	0.0044	nucleotide binding and protein kinase C binding
HIST1H2BA	histone cluster 1, H2ba	93889_f_at	0.7896	0.0342	Alcoholism
HJURP	Holliday junction recognition protein	160682_at	0.7233	0.0111	Cell Cycle, Mitotic and Packaging Of Telomere Ends
ID2	inhibitor of DNA binding 2	93013_at	0.6823	0.0360	Hippo_signaling_pathway
IFNAR2	interferon (alpha and beta) receptor 2	101014_at	0.7512	0.0414	Cytokine_cytokine_receptor_interaction
KIT	kit oncogene	99956_at	0.7972	0.0156	Hematopoietic_cell_lineage
KRT8	keratin 8	101009_at	0.7934	0.0457	Cytoskeletal Signaling and EGFR1 Signaling Pathway
MAP3K1	mitogen activated protein kinase kinase kinase 1	103021_r_at	0.7891	0.0100	GnRH_signaling_pathway
MID1IP1	Mid1 interacting protein 1 (gastrulation specific G12-like (zebrafish))	95135_at	0.7149	0.0471	protein C-terminus binding
MPDZ	multiple PDZ domain protein	93887_at	0.7500	0.0350	Tight_junction
MRPL48	mitochondrial ribosomal protein L48	96059_at	0.7887	0.0088	Structural constituent of ribosome
MRPS24	mitochondrial ribosomal protein S24	101085_at	0.7870	0.0427	Structural constituent of ribosome
MTM1	X-linked myotubular myopathy gene 1	96199_at	0.7966	0.0434	Inositol_phosphate_metabolism
NDUFA6	NADH dehydrogenase (ubiquinone) 1 alpha subcomplex, 6 (B14)	160237_at	0.7902	0.0497	Oxidative_phosphorylation
NEDD8	neural precursor cell expressed, developmentally down-regulated ge	93519_s_at	0.7903	0.0394	Signaling by GPCR
NME1	NME/NM23 nucleoside diphosphate kinase 1	92794_f_at	0.7649	0.0186	Purine_metabolism, pyrimidine_metabolism
NR2F2	nuclear receptor subfamily 2, group F, member 2	103052_r_at	0.7344	0.0361	L1CAM interactions, Oct4 in mammalian ESC pluripotency

NSUN4	NOL1/NOP2/Sun domain family, member 4	97434_at	0.7822	0.0113	RNA binding and methyltransferase activity
PEMT	phosphatidylethanolamine N-methyltransferase	94987_at	0.7856	0.0397	Glycerophospholipid_metabolism
PERP	p53 apoptosis effector related to Pmp22	97825_at	0.7554	0.0181	p53_signaling_pathway
PKP2	plakophilin 2	104338_r_at	0.7678	0.0428	Arrhythmogenic_right_ventricular_cardiomyopathy_(ARVC)
PPIB	peptidylprolyl isomerase B	94915_at	0.7941	0.0435	Degradation of the ECM and RANK signaling
PRDX4	peroxiredoxin 4	93495_at	0.7394	0.0434	Selenium pathway and Prostaglandin 2 metabolism
PRKD3	protein kinase D3	160632_at	0.7060	0.0201	Rap1_signaling_pathway
PRODH2	proline oxidase 1	103452_at	0.7800	0.0265	Arginine_and_proline_metabolism
PTPRD	protein tyrosine phosphatase, receptor type, D	93485_at	0.7977	0.0347	PAK Pathway
RAB11A	RAB11a, member RAS oncogene family	92854_at	0.7800	0.0112	Membrane traficking
RASIP1	Ras interacting protein 1	104146_at	0.7738	0.0216	Vasculogenesis and angiogenesis.
RBL2	retinoblastoma-like 2	95617_at	0.7656	0.0316	Cell_cycle
RBP1	retinol binding protein 1, cellular	104716_at	0.7309	0.0348	Transporter activity and retinal binding
RHPN2	rhophilin, Rho GTPase binding protein 2	98485_at	0.6179	0.0413	Signal transduction
ROMO1	reactive oxygen species modulator 1	96687_f_at	0.7461	0.0076	Production of reactive oxygen species
RPL10A	ribosomal protein L10A	100711_at	0.7543	0.0466	Ribosome
RPL13	ribosomal protein L13	102109_at	0.7397	0.0336	Ribosome
RPL22L1	ribosomal protein L22 like 1	93987_f_at	0.5923	0.0361	Ribosome
RPL23A	ribosomal protein L23A	94823_at	0.7769	0.0149	Ribosome
RPL26	ribosomal protein L26	100729_at	0.7837	0.0026	Ribosome
RPL27A	ribosomal protein L27a	101573_f_at	0.7481	0.0203	Ribosome
RPL27A	ribosomal protein L27a	101680_at	0.6794	0.0324	Ribosome
RPL29	ribosomal protein L29	94240_i_at	0.7777	0.0102	Ribosome
RPL30	ribosomal protein L30	98119_at	0.7660	0.0417	Ribosome
RPL34	ribosomal protein L34	96307_s_at	0.7566	0.0291	Ribosome
RPL37	ribosomal protein L37	92577_f_at	0.7535	0.0443	Ribosome
RPL8	ribosomal protein L8	96575_at	0.7850	0.0044	Ribosome
RPS10	ribosomal protein S10	99093_at	0.7610	0.0312	Ribosome
RPS11	ribosomal protein S11	94767_at	0.7709	0.0008	Ribosome
RPS12	ribosomal protein S12	102126_at	0.6923	0.0116	Ribosome
RPS16	ribosomal protein S16	97647_at	0.7073	0.0100	Ribosome
RPS17	ribosomal protein S17	99590_at	0.7544	0.0156	Ribosome
RPS18	procollagen, type XI, alpha 2	98333_at	0.6859	0.0157	Ribosome
RPS23	ribosomal protein S23	96358_at	0.7634	0.0014	Ribosome
RPS28	H2-K region expressed gene 2	100758_at	0.7388	0.0186	Ribosome
RPS7	ribosomal protein S7	101212_at	0.7713	0.0055	Ribosome
S100A13	S100 calcium binding protein A13	100959_at	0.7697	0.0302	Calcium ion binding and lipid binding
SELENBP1	selenium binding protein 1	100596_at	0.5873	0.0225	Selenium Metabolism and Selenoproteins
SESN1	sestrin 1	95731_at	0.6982	0.0074	p53_signaling_pathway
SHFM1	split hand/foot deleted gene 1	95456_r_at	0.7782	0.0377	Homologous_recombination
SIRT3	sirtuin 3 (silent mating type information regulation 2, homolog) 3 (S.	160869_at	0.7627	0.0056	Central_carbon_metabolism_in_cancer
SLC22A30	solute carrier family 22, member 30	103703_f_at	0.7965	0.0317	sodium-independent organic anion transport
SLIRP	SRA stem-loop interacting RNA binding protein	96743_at	0.7236	0.0494	RNA binding and nucleotide binding
SNHG6	small nucleolar RNA host gene 6	96156_at	0.6984	0.0039	
SNRPD2	small nuclear ribonucleoprotein D2	95049_at	0.7774	0.0314	Spliceosome
SPATA13	spermatogenesis associated 13	100958_at	0.7011	0.0172	G-protein signaling_Regulation of CDC42 activity
STAG2	stromal antigen 2	104595_at	0.7964	0.0352	Cell_cycle
TALDO1	transaldolase 1	95066_at	0.7767	0.0439	Pentose_phosphate_pathway
TCEB2	transcription elongation factor B (SIII), polypeptide 2	160402_at	0.7467	0.0468	HIF_1_signaling_pathway
THOC3	THO complex 3	99490_at	0.7916	0.0197	RNA_transport, spliceosome
TIA1	cytotoxic granule-associated RNA binding protein 1	98085_f_at	0.7888	0.0030	IL-10 Pathway
TIMD2	T cell immunoglobulin and mucin domain containing 2	103794_i_at	0.7867	0.0219	immune response
TIMD2	T cell immunoglobulin and mucin domain containing 2	97335_at	0.7984	0.0198	immune response

TMEM97	transmembrane protein 97	95137_at	0.7340	0.0293	cholesterol homeostasis
TYMP	thymidine phosphorylase	160292_at	0.7102	0.0436	NF-KappaB Family Pathway
UBL5	ubiquitin-like 5	94268_f_at	0.7863	0.0256	mRNA splicing, protein targeting to mitochondrion
UBL5	ubiquitin-like 5	94267_i_at	0.6658	0.0130	mRNA splicing, protein targeting to mitochondrion
XPA	xeroderma pigmentosum, complementation group A	92384_at	0.7862	0.0032	Nucleotide_excision_repair
ZAP70	zeta-chain (TCR) associated protein kinase (70kD)	93661_at	0.7439	0.0137	T_cell_receptor_signaling_pathway
ZAP70	zeta-chain (TCR) associated protein kinase (70kD)	93662_s_at	0.7346	0.0004	T_cell_receptor_signaling_pathway
ZBTB48	zinc finger and BTB domain containing 48	103765_at	0.7539	0.0416	Regulation of transcription, DNA-templated
ZFP871	zinc finger protein 871	104327_at	0.6605	0.0234	

Microarray gene analysis was conducted as described in the Method section. The probes used to study individual genes are listed along with the gene symbols and gene names. Some genes may have more than one probe. FC stands for fold of change over control (non-DEN treated). P values refer to the significance test. Genes listed in this table have FC of <0.80 with a *p* value <0.05. The function of the genes were obtained via multiple bioinformatics sources. Only main functions are listed. Not all genes have a clearly defined function
