# Supplementary material for: Gene Expression Analysis Indicates Divergent Mechanisms in DEN-Induced Carcinogenesis in Wild Type and Bid-Deficient Livers
Source: PLoS One. 2016 May 19;11(5):e0155211. doi: 10.1371/journal.pone.0155211 (PMC4873180; doi:10.1371/journal.pone.0155211)
Supplement: S7 Table — (PDF) [file pone.0155211.s007.pdf]

S7 Table. Up-regulated genes in livers of Bid-deficient mice treated with DEN for 10-12 months

Genes Symbol	Gene Name	Probes	FC	p value	Function
2700097O09RIK	RIKEN cDNA 2700097O09 gene	160980_at	1.3632	0.0485	Methylation
ABCB7	ATP-binding cassette, sub-family B (MDR/TAP), member 7	103300_at	1.3669	0.0440	ABC_transporters
ACTR6	ARP6 actin-related protein 6	160666_at	1.4174	0.0103	Chromatin remodeling
AKAP8	A kinase (PRKA) anchor protein 8	94390_at	1.3083	0.0019	Activation of cAMP-Dependent PKA
ATP13A3	ATPase type 13A3	103667_at	1.9039	0.0091	Cation transport, cellular calcium ion homeostasis
AW549877	expressed sequence AW549877	103895_at	1.3719	0.0254	Human ortholog is C5orf51
BIRC5	baculoviral IAP repeat-containing 5	101521_at	1.2735	0.0468	Hippo_signaling_pathway
CASP3	caspase 3	98437_at	1.2503	0.0313	Apoptosis
CASP8AP2	caspase 8 associated protein 2	104290_at	1.3157	0.0222	TWEAK pathway, TNF-alpha/NF-kB pathway
CCDC90B	coiled-coil domain containing 90B	103873_i_at	1.2679	0.0403	
CCNC	cyclin C	99558_at	1.2621	0.0224	Signaling by GPCR and Disease
CETN3	centrin 3	92788_f_at	1.3739	0.0478	calcium binding and G-protein beta/gamma binding
CFDP1	craniofacial development protein 1	93784_at	1.3065	0.0341	Cell adhesion, cell shape, cell proliferation
CLCN4	chloride channel, voltage-sensitive 4	104704_at	1.3253	0.0166	Activation of cAMP-Dependent PKA
CLK1	CDC-like kinase	93274_at	1.5750	0.0162	Legionellosis
CLK4	CDC like kinase 4	101936_at	1.4602	0.0057	mRNA Splicing - Major Pathway
CUL1	cullin 1	94002_at	1.4110	0.0384	Cell_cycle
D030029J20RIK	RIKEN cDNA D030029J20 gene	96526_at	1.3800	0.0222	
DBT	dihydrolipoamide branched chain transacylase E2	98966_at	1.6458	0.0427	Valine,_leucine_and_isoleucine_degradation
DNAJC13	DnaJ heat shock protein family (Hsp40) member C13	94422_at	1.5047	0.0336	osteoblast differentiation, endosome organization
EFNB2	ephrin B2	160857_at	1.3235	0.0200	Axon_guidance
EIF1AX	eukaryotic translation initiation factor 1A, X-linked	160111_at	1.2923	0.0309	Apoptosis, biosynthesis of the N-glycan precursor
EIF3E	eukaryotic translation initiation factor 3, subunit E	160554_at	1.4432	0.0355	p70S6K signaling, apoptosis
EIF5	eukaryotic translation initiation factor 5	160265_at	1.5873	0.0358	RNA_transport
FABP2	fatty acid binding protein 2, intestinal	97889_at	1.4586	0.0405	Fat_digestion_and_absorption
FAM103A1	family with sequence similarity 103, member A1	93059_at	1.2791	0.0461	RNA binding
FAM13B	family with sequence similarity 13, member B	104119_at	1.4319	0.0266	Signaling by GPCR and Rho GTPase cycle
FAM76B	family with sequence similarity 76, member B	104193_at	1.4154	0.0406	Protein desumoylation
FAM8A1	family with sequence similarity 8, member A1	97897_at	1.4156	0.0130	
GKAP1	G kinase anchoring protein 1	160900_at	1.4976	0.0244	Signal transduction
GM12966	predicted gene 12966	99628_at	1.5592	0.0291	
GOLGA4	golgi autoantigen, golgin subfamily a, 4	102060_at	1.3642	0.0213	GTPase binding
GTPBP2	GTP binding protein 2	104144_at	1.2528	0.0225	GTP binding and GTPase activity
GYK	glycerol kinase	97525_at	1.2638	0.0425	Glycerolipid_metabolism
H19	H19, imprinted maternally expressed transcript	93028_at	3.6046	0.0373	Cell proliferation, gene expression
HMGB3	high mobility group box 3	98038_at	1.3731	0.0184	DNA binding and bending
HNRNPA1	heterogeneous nuclear ribonucleoprotein A1	92724_at	1.5623	0.0041	Translational control and gene expression
HNRNPDL	heterogeneous nuclear ribonucleoprotein D-like	96083_s_at	1.6247	0.0069	RNA binding and double-stranded DNA binding
HNRNPH1	heterogeneous nuclear ribonucleoprotein H1	93990_at	1.9114	0.0309	Gene Expression and mRNA Splicing - Major Pathway
HNRNPM	heterogeneous nuclear ribonucleoprotein M	96266_at	1.3745	0.0114	Gene Expression and Spliceosome
HRSP12	heat-responsive protein 12	96048_at	1.7095	0.0413	endonuclease activity and deaminase activity
IIGP1	interferon-inducible GTPase	96764_at	1.8264	0.0047	immune system process, regulation of autophagy
INTS8	integrator complex subunit 8	104142_at	1.2698	0.0134	snRNA processing
Kat2b	K(lysine) acetyltransferase 2B	104070_at	1.5550	0.0440	Signaling by GPCR and Disease
KLHL9	kelch-like 9	160198_at	1.3862	0.0434	Ubiquitin_mediated_proteolysis
KLKB1	kallikrein B, plasma 1	104344_at	1.3219	0.0246	Complement_and_coagulation_cascades
LIFR	leukemia inhibitory factor receptor	103697_at	1.4945	0.0204	GPCR Pathway
LIN7C	lin-7 homolog C (C. elegans)	103370_at	1.4249	0.0138	Cytoskeletal protein binding
LPGAT1	lysophosphatidylglycerol acyltransferase 1	104260_at	1.3585	0.0042	Metabolism
MARCH7	membrane associated ring-CH-type finger 7	160103_at	1.5332	0.0365	
MBTD1	mbt domain containing 1	94352_at	1.5524	0.0102	Transcription, DNA-templated

MCAM	melanoma cell adhesion molecule	160458_at	1.2879	0.0054	Angiogenesis, cell adhesion
MGEA5	meningioma expressed antigen 5 (hyaluronidase)	94499_at	1.3622	0.0348	Histone acetyltransferase, hyaluronoglucosaminidase
MUT	methylmalonyl-Coenzyme A mutase	99613_at	1.3509	0.0033	Glyoxylate_and_dicarboxylate_metabolism
MYO1B	myosin Ib	98409_at	1.4364	0.0297	RhoGDI Pathway
NCL	nucleolin	100144_at	1.7474	0.0376	Insulin regulation of translation
NFIB	nuclear factor I/B	99440_at	1.4233	0.0176	RNA Polymerase I promoter opening and gene expression
NFIB	nuclear factor I/B	160859_s_at	1.3085	0.0418	RNA Polymerase I promoter opening and gene expression
NFKBIZ	molecule possessing ankyrin-repeats induced by lipopolysaccharide	98988_at	1.2829	0.0352	Transcriptional_misregulation_in_cancer
NIPBL	Nipped-B homolog (Drosophila)	102895_at	1.4203	0.0432	Cell Cycle, Mitotic
NPM1	nucleophosmin 1	101634_at	1.9320	0.0200	Disease and Cell Cycle, Mitotic
NRBP2	nuclear receptor binding protein 2	102207_at	1.2586	0.0306	nucleotide binding and protein kinase activity
NXF1	nuclear RNA export factor 1 homolog (S. cerevisiae)	101079_at	1.2516	0.0143	mRNA_surveillance_pathway
OGT	BTB (POZ) domain containing 1	94818_at	1.5585	0.0278	Other_types_of_O_glycan_biosynthesis
OTUD4	OTU domain containing 4	94923_f_at	1.2850	0.0253	Ubiquitin-specific protease activity
PAXBP1	PAX3 and PAX7 binding protein 1	92233_at	1.3430	0.0181	DNA binding transcription factor activity
PBRM1	polybromo 1	98893_at	1.3494	0.0310	chromatin binding
PDCD4	programmed cell death 4	103029_at	1.5554	0.0241	MicroRNAs_in_cancer
PELI1	pellino 1	94505_at	1.5640	0.0186	Interferon signaling and TLR7/8 cascade
PHLDB2	pleckstrin homology-like domain, family B, member 2	102907_at	1.5953	0.0405	Regulation of Microtubule Cytoskeleton
PLEKHB1	pleckstrin homology domain containing, family B (evectins) member 1	93424_at	1.4612	0.0335	Protein homodimerization and signal transducer activity
PNRC2	proline-rich nuclear receptor coactivator 2	95059_at	1.4499	0.0364	Transcription, DNA-templated
PSPC1	paraspeckle protein 1	103393_at	1.2564	0.0003	RNA binding and nucleotide binding
PUM2	pumilio RNA-binding family member 2	93580_at	1.3269	0.0496	Translational Control
RBM39	RNA binding motif protein 39	93267_at	1.5615	0.0220	mRNA Splicing - Major Pathway
RBMX	RNA binding motif protein, X chromosome retrogene	160192_at	1.5226	0.0142	Spliceosome
REV3L	REV3-like, catalytic subunit of DNA polymerase zeta RAD54 like (S. cerevisiae)	103457_at	1.3077	0.0449	Fanconi_anemia_pathway
RNF141	ring finger protein 141	101939_at	1.3066	0.0384	Gene Expression
RPL7	ribosomal protein L7	97696_r_at	1.5151	0.0325	Ribosome
SDC2	syndecan 2	95104_at	1.2865	0.0216	Cell_adhesion_molecules_(CAMs)
SELT	selenoprotein T	97292_at	1.4358	0.0355	Selenium metabolism and Selenoproteins
SFRS11	serine/arginine-rich splicing factor 11	96609_at	1.5297	0.0011	Gene Expression and mRNA Splicing - Major Pathway
SLC30A4	solute carrier family 30 (zinc transporter), member 4	95571_at	1.3327	0.0074	Zinc ion transmembrane transporter activity
SMARCA5	SWI/SNF related, matrix associated, actin dependent regulator of chromatin, sub	97394_at	1.3952	0.0304	Cell Cycle, Mitotic and PEDF Induced Signaling
SMC3	chondroitin sulfate proteoglycan 6	102853_at	1.2935	0.0091	Cell_cycle
SMC4	structural maintenance of chromosomes 4	101906_at	1.5062	0.0415	Cell Cycle, Mitotic
SNORD22	RNA, U22 small nucleolar	97838_at	1.4647	0.0307	
SNX5	sorting nexin 5	93598_at	1.2676	0.0308	Clathrin derived vesicle budding
SON	Son DNA binding protein	94386_at	1.3661	0.0091	RNA binding and RS domain binding
SPIC	Spi-C transcription factor (Spi-1/PU.1 related)	103454_at	1.2940	0.0165	Sequence-specific DNA binding transcription factor activity
SS18	synovial sarcoma translocation, Chromosome 18	99610_at	1.3886	0.0328	Transcriptional_misregulation_in_cancer
SYNJ2BP	synaptojanin 2 binding protein	96734_at	1.2665	0.0021	Protein C-terminus and type II activin receptor binding
TAB3	TGF-beta activated kinase 1/MAP3K7 binding protein 3	160982_at	1.2711	0.0041	Metal ion binding
TCF4	transcription factor 4	160483_at	1.3799	0.0466	L1CAM interactions and ERK Signaling
TMEM56	transmembrane protein 56	103257_at	1.5869	0.0108	
TMEM68	transmembrane protein 68	104620_at	1.3409	0.0397	Transferase activity, transferring acyl groups
TNKS2	tankyrase, TRF1-interacting ankyrin-related ADP-ribose polymerase 2	102912_at	1.5552	0.0287	Apoptosis, nicotinate and nicotinamide metabolism
TUG1	taurine upregulated gene 1	103400_at	1.8949	0.0081	
USP15	ubiquitin specific peptidase 15	95416_at	1.2627	0.0491	Signaling by GPCR and Disease
USP33	ubiquitin specific peptidase 33	160710_at	1.3502	0.0225	Ubiquitin-Proteasome Dependent Proteolysis
USP34	ubiquitin specific peptidase 34	102647_g_at	1.2558	0.0389	Ubiquitin-Proteasome Dependent Proteolysis
WBP5	WW domain binding protein 5	100522_s_at	2.4756	0.0363	WW domain binding
YIPF4	Yip1 domain family, member 4	160475_at	1.3159	0.0372	
YIPF5	Yip1 domain family, member 5	94273_at	1.5571	0.0273	ER-Golgi trafficking
YTHDC1	YTH domain containing 1	160313_at	1.3431	0.0133	mRNA splicing, via spliceosome

ZFP51	zinc finger protein 51	102872_f_at	1.4094	0.0424	Regulation of transcription, DNA-templated
ZFP62	zinc finger protein 62	103063_at	1.7055	0.0109	Nucleic acid binding
ZFP68	Zinc finger protein 68	95521_s_at	1.2679	0.0443	Regulation of transcription, DNA-templated
ZFP871	zinc finger protein 871	104327_at	1.5768	0.0310	
ZFP97	zinc finger protein 97	98761_i_at	1.5705	0.0239	
ZFR	zinc finger RNA binding protein	104050_at	1.3594	0.0114	Diurnally regulated genes with circadian orthologs
ZMYM6	zinc finger, MYM-type 6	104263_at	1.4834	0.0426	Cytoskeleton organization, cell morphogenesis
ZNRF2	zinc and ring finger 2	104396_at	1.3597	0.0436	Ligase activity
ZSCAN26	zinc finger and SCAN domain containing 26	160966_at	1.3275	0.0223	Sequence-specific DNA binding transcription factor activity
ZZZ3	zinc finger, ZZ domain containing 3	103753_at	1.2560	0.0238	RNA Polymerase I Promoter Opening

Microarray gene analysis was conducted as described in the Method section. The probes used to study individual genes are listed along with the gene symbols and gene names. Some genes may have more than one probe. FC stands for fold of change over control (non-DEN treated). P values refer to the significance test. Genes listed in this table have FC of >1.25 with a *p* value <0.05. The function of the genes were obtained via multiple bioinformatics sources. Only main functions are listed. Not all genes have a clearly defined function.
