# Supplementary material for: Gene Expression Analysis Indicates Divergent Mechanisms in DEN-Induced Carcinogenesis in Wild Type and Bid-Deficient Livers
Source: PLoS One. 2016 May 19;11(5):e0155211. doi: 10.1371/journal.pone.0155211 (PMC4873180; doi:10.1371/journal.pone.0155211)
Supplement: S8 Table — (PDF) [file pone.0155211.s008.pdf]

S8 Table. Down-regulated genes in livers of Bid-deficient pe mice treated with DEN for 10-12 months

Genes Symbol	Gene Name	Probes	FC	p value	Function
4933416E03RIK	RIKEN cDNA 4933416E03 gene	98358_at	0.6932	0.0016	
AARS	alanyl-tRNA synthetase	94918_at	0.7498	0.0448	Aminoacyl_tRNA_biosynthesis
ADRA2A	adrenergic receptor, alpha 2a	102556_at	0.7922	0.0423	cGMP_PKG_signaling_pathway
AGPAT6	putative lysophosphatidic acid acyltransferase	100996_at	0.7989	0.0361	MAPK_signaling_pathway
ALAS1	aminolevulinic acid synthase 1	93500_at	0.4648	0.0312	Metabolism
ANGPTL1	angiopoietin-like 1	95228_f_at	0.7086	0.0000	TGF-Beta Pathway
B4GALNT2	beta-1,4-N-acetyl-galactosaminyl transferase 2	96548_at	0.7789	0.0327	Acetylgalactosaminyltransferase activity
BCL3	B-cell leukemia/lymphoma 3	102239_at	0.7242	0.0244	TNF_signaling_pathway
BCL7B	B-cell CLL/lymphoma 7B	97490_at	0.7583	0.0466	Actin binding
CAPN3	calpain 3	99812_at	0.7917	0.0272	ERK Signaling and Apoptosis Pathway
CD36	CD36 antigen	93332_at	0.7748	0.0330	Adipocytokine_signaling_pathway
CGA	glycoprotein hormones, alpha subunit	162502_f_at	0.7784	0.0490	GnRH_signaling_pathway
CHGA	chromogranin A	92422_at	0.7592	0.0135	Signaling by GPCR
COG8	component of oligomeric golgi complex 8	98097_r_at	0.7274	0.0304	Transport
DEF8	differentially expressed in FDCP 8	160490_at	0.7658	0.0383	Intracellular signal transduction
DUSP1	dual specificity phosphatase 1	104598_at	0.6923	0.0459	MAPK signaling pathway and ERK Signaling
FBXO15	F-box protein 15	96162_at	0.7673	0.0388	Oct4 in Mammalian ESC Pluripotency
FCGRT	Fc receptor, IgG, alpha chain transporter	97533_at	0.7862	0.0368	Antigen binding and beta-2-microglobulin binding
FMO5	flavin containing monooxygenase 5	162259_f_at	0.7564	0.0387	Drug_metabolism_cytochrome_P450
HCFC1R1	host cell factor C1 regulator 1 (XPO1-dependent)	94266_at	0.7409	0.0494	
HSPB8	heat shock protein 8	162448_f_at	0.6773	0.0305	Protein conformation
ILVBL	ilvB (bacterial acetolactate synthase)-like	161738_f_at	0.7765	0.0417	Magnesium ion and thiamine pyrophosphate binding
ITGA7	integrin alpha 7	160086_at	0.7841	0.0341	Focal_adhesion
JAM3	junction cell adhesion molecule 2	98957_at	0.7621	0.0421	Cell_adhesion_molecules_(CAMs)
LGALS9	lectin, galactose binding, soluble 9	161301_f_at	0.7270	0.0437	Signal transducer activity and galactose binding
MCART1	tripartite motif protein 8	161951_f_at	0.7146	0.0393	Complement_and_coagulation_cascades
METTL1	methyltransferase like 1	99499_at	0.7666	0.0163	tRNA binding and tRNA (guanine-N7-)-methyltransferase activity
NAP1L4	nucleosome assembly protein 1-like 4	161505_i_at	0.7835	0.0121	Unfolded protein binding
NDN	necdin	101059_at	0.7529	0.0475	p75(NTR)-mediated signaling and Adipogenesis
PTMS	parathymosin	95723_r_at	0.6429	0.0223	Immune system process
RSPO1	R-spondin 1	98312_at	0.7728	0.0105	Wnt signaling network
SERPINI1	serine (or cysteine) peptidase inhibitor, clade I, member 1	99494_at	0.7709	0.0331	Cell adhesion_Plasmin signaling
SNRPN	small nuclear ribonucleoprotein N	102163_at	0.7894	0.0496	mRNA splicing, via spliceosome
SULT5A1	sulfotransferase family 5A, member 1	93434_at	0.5753	0.0398	Phenanthrol sulfotransferase activity
SWAP70	SWA-70 protein	93218_at	0.7716	0.0404	Calcium ion binding and phospholipid binding
TEAD2	TEA domain family member 2	96940_at	0.7669	0.0418	Hippo signaling pathway and Metabolism
THBD	thrombomodulin	162023_f_at	0.7282	0.0317	Hippo_signaling_pathway
TYSND1	trypsin domain containing 1	104164_at	0.7331	0.0478	Glycerolipid_metabolism
USP2	ubiquitin specific protease 2	92820_at	0.7380	0.0471	Cell Cycle / Checkpoint Control and Protein Stability

Microarray gene analysis was conducted as described in the Method section. The probes used to study individual genes are listed along with the gene symbols and gene names. Some genes may have more than one probe. FC stands for fold of change over control (non-DEN treated). P values refer to the significance test. Genes listed in this table have FC of <0.80 with a *p* value <0.05. The function of the genes were obtained via multiple bioinformatics sources. Only main functions are listed. Not all genes have a clearly defined function
