# Supplementary material for: Gene Expression Analysis Indicates Divergent Mechanisms in DEN-Induced Carcinogenesis in Wild Type and Bid-Deficient Livers
Source: PLoS One. 2016 May 19;11(5):e0155211. doi: 10.1371/journal.pone.0155211 (PMC4873180; doi:10.1371/journal.pone.0155211)
Supplement: S9 Table — (PDF) [file pone.0155211.s009.pdf]

S9 Table. DAVID pathway analsis of differentially expressed genes in DEN-treated mouse livers

Genotype	Time	Expression	Gene List	# of Genes	# of Pathway	KEGG Pathway	Count	p value	FDR
**wild type**	4-6 month	Increased	Table S1	62	1	mmu04514:Cell adhesion molecules (CAMs)	3	0.090879	61.38094
		Decreased	Table S2	27	1	mmu00350:Tyrosine metabolism	2	0.072336	41.32808
	10-12-month	Increased	Table S3<br>Table S10	227	10	mmu00030:Pentose phosphate pathway	4	0.007942	8.529313
						mmu00480:Glutathione metabolism	5	0.009318	9.938112
						mmu04142:Lysosome	7	0.011739	12.36882
						mmu04670:Leukocyte transendothelial migration	7	0.011739	12.36882
						mmu05020:Prion diseases	4	0.018027	18.40534
						mmu04610:Complement and coagulation cascades	5	0.031713	30.25613
						mmu04640:Hematopoietic cell lineage	5	0.045214	40.38902
						mmu04666:Fc gamma R-mediated phagocytosis	5	0.071688	56.4709
						mmu04620:Toll-like receptor signaling pathway	5	0.073828	57.5802
						mmu05322:Systemic lupus erythematosus	5	0.082714	61.9147
		Decreased	Table S4<br>Table S11	381	29	mmu00190:Oxidative phosphorylation	19	4.77E-08	5.61E-05
						mmu00071:Fatty acid metabolism	12	5.68E-08	6.67E-05
						mmu00280:Valine, leucine and isoleucine degradation	12	7.27E-08	8.54E-05
						mmu05012:Parkinson's disease	18	3.77E-07	4.43E-04
						mmu05010:Alzheimer's disease	21	4.15E-07	4.88E-04
						mmu05016:Huntington's disease	18	3.10E-05	0.036431
						mmu00120:Primary bile acid biosynthesis	6	5.52E-05	0.064922
						mmu00380:Tryptophan metabolism	8	1.61E-04	0.189367
						mmu00310:Lysine degradation	7	0.001288	1.503739
						mmu00982:Drug metabolism	9	0.00175	2.037629
						mmu00980:Metabolism of xenobiotics by cytochrome P450	8	0.003489	4.024642
						mmu00790:Folate biosynthesis	4	0.003718	4.283521
						mmu00650:Butanoate metabolism	6	0.004687	5.372944
						mmu04260:Cardiac muscle contraction	8	0.008756	9.820746
						mmu00140:Steroid hormone biosynthesis	6	0.01083	12.01375
						mmu00640:Propanoate metabolism	5	0.011856	13.08048
						mmu00830:Retinol metabolism	7	0.016084	17.35338
						mmu00680:Methane metabolism	3	0.017178	18.42653
						mmu00410:beta-Alanine metabolism	4	0.027222	27.70478
						mmu00350:Tyrosine metabolism	5	0.028885	29.14363
						mmu03320:PPAR signaling pathway	7	0.031215	31.11627
						mmu00620:Pyruvate metabolism	5	0.033963	33.37825
						mmu04950:Maturity onset diabetes of the young	4	0.042122	39.69978
						mmu00300:Lysine biosynthesis	2	0.059396	51.31219
						mmu00260:Glycine, serine and threonine metabolism	4	0.070578	57.69714
						mmu00480:Glutathione metabolism	5	0.070607	57.71252
						mmu00903:Limonene and pinene degradation	3	0.073394	59.17942
						mmu00330:Arginine and proline metabolism	5	0.074657	59.82855
						mmu00150:Androgen and estrogen metabolism	4	0.075985	60.50095

bid -/-	4-6 month	Increased	Table S5	36	3	mmu05414:Dilated cardiomyopathy	3	0.032936	28.33809
						mmu00190:Oxidative phosphorylation	3	0.061523	46.83394
						mmu04950:Maturity onset diabetes of the young	2	0.078607	55.71561
		Decreased	Table S6	106	1	mmu03010:Ribosome	19	8.35E-20	8.61E-17
	10-12-month	Increased	Table S7	114	2	mmu03320:PPAR signaling pathway	3	0.036188	27.51004
						mmu03040:Spliceosome	3	0.080812	52.07345
		Decreased	Table S8	39	0				

1. Genes differentially expressed in DEN-treated wild type or *bid* -deficient livers for 4-6 months or for 10-12 months were subjected to DAVID analysis using KEGG pathway designation. The up-regulated or down-regulated genes are listed in S1-8 Tables. Some are further elaborated in S10-11 Tables.

2. For the wild type 10-12 month group, 6 out of the 10 pathways associated with the up-regualted genes are related to immune system (in red font); whereas 23 out of the 29 pathways associated with the down-regulated genes are related to metabolism regulation (in red or in green font), in which 8 are related to amino acid regulation (in red font).

3. Counts: number of genes in the gene set related to the pathway; p value: the statistical signficance of the enrichment; FDR: false discovery rate: the estimated probability that the normalized enrichment represents a false positive finding.

S10 Table. Genes that are involved in immune response and/or inflammation, and that are upregulated in WT mice treated with DEN for 10-12 Months

Gene Symbol	Gene Name	Probe ID	Fold of Change	p value	Pathways	Functional Class
C1QA	complement component 1, q subcomponent, alpha polypeptide	98562_at	1.6453	0.0269	Complement_and_coagulation_cascades	Organismal Systems: Immune System
					Systemic_lupus_erythematosus	Human Diseases: Immune diseases
					Chagas_disease_(American_trypanosomiasis)	Human Diseases: Infectious diseases
					Pertussis	Human Diseases: Infectious diseases
					Staphylococcus_aureus_infection	Human Diseases: Infectious diseases
					Prion_diseases	Human Diseases: Neurodegenerative diseases
C1QB	complement component 1, q subcomponent, beta polypeptide	96020_at	2.0625	0.0271	Complement_and_coagulation_cascades	Organismal Systems: Immune System
					Systemic_lupus_erythematosus	Human Diseases: Immune diseases
					Chagas_disease_(American_trypanosomiasis)	Human Diseases: Infectious diseases
					Pertussis	Human Diseases: Infectious diseases
					Staphylococcus_aureus_infection	Human Diseases: Infectious diseases
					Prion_diseases	Human Diseases: Neurodegenerative diseases
C1QC	complement component 1, q subcomponent, c polypeptide	92223_at	1.7362	0.0065	Complement_and_coagulation_cascades	Organismal Systems: Immune System
					Systemic_lupus_erythematosus	Human Diseases: Immune diseases
					Chagas_disease_(American_trypanosomiasis)	Human Diseases: Infectious diseases
					Pertussis	Human Diseases: Infectious diseases
					Staphylococcus_aureus_infection	Human Diseases: Infectious diseases
					Prion_diseases	Human Diseases: Neurodegenerative diseases
CCL4	small inducible cytokine A4	94146_at	1.2846	0.0351	Toll_like_receptor_signaling_pathway	Organismal Systems: Immune System
					Chemokine_signaling_pathway	Organismal Systems: Immune System
					Cytosolic_DNA_sensing_pathway	Organismal Systems: Immune System
					Cytokine_cytokine_receptor_interaction	Environmental Information Processing: Signaling molecules and interation
					NF_kappa_B_signaling_pathway	Environmental Information Processing: Signal transdution
					Salmonella_infection	Human Diseases: Infectious diseases
CCL5	small inducible cytokine A5	98406_at	1.4894	0.0475	TNF_signaling_pathway	Environmental Information Processing: Signal transdution
					Cytokine_cytokine_receptor_interaction	Environmental Information Processing: Signaling molecules and interation
					Chemokine_signaling_pathway	Organismal Systems: Immune System

					NOD_like_receptor_signaling_pathway	Organismal Systems: Immune System
					Cytosolic_DNA_sensing_pathway	Organismal Systems: Immune System
					Rheumatoid_arthritis	Human Diseases: Immune diseases
					Herpes_simplex_infection	Human Diseases: Infectious diseases
					Influenza_A	Human Diseases: Infectious diseases
					Chagas_disease_(American_trypanosomiasis)	Human Diseases: Infectious diseases
					Prion_diseases	Human Diseases: Neurodegenerative diseases
CD14	CD14 antigen	98088_at	2.0174	0.0178	Hematopoietic_cell_lineage	Organismal Systems: Immune System
					Toll_like_receptor_signaling_pathway	Organismal Systems: Immune System
					NF_kappa_B_signaling_pathway	Environmental Information Processing: Signal transdution
					Amoebiasis	Human Diseases: Infectious diseases
					Legionellosis	Human Diseases: Infectious diseases
					Pertussis	Human Diseases: Infectious diseases
					Salmonella_infection	Human Diseases: Infectious diseases
					MAPK_signaling_pathway	Environmental Information Processing: Signal transdution
					Phagosome	Cellular Process: Transport and catabolism
					Regulation_of_actin_cytoskeleton	Cellular Process: Cell motility
CD99	CD99 antigen	101047_at	1.6316	0.0426	Leukocyte_transendothelial_migration	Organismal Systems: Immune System
					Cell_adhesion_molecules_(CAMs)	Environmental Information Processing: Signaling molecules and interation
CYBA	cytochrome b-245, alpha polypeptide	100059_at;	1.4238	0.0014	Leukocyte_transendothelial_migration	Organismal Systems: Immune System
		97013_f_at	1.5608	0.0332	Leishmaniasis	Human Diseases: Infectious diseases
					Osteoclast_differentiation	Organismal Systems: Development
					Phagosome	Cellular Process: Transport and catabolism
DNTT	deoxynucleotidyltransferase, terminal	103962_at	1.2917	0.0254	Hematopoietic_cell_lineage	Organismal Systems: Immune System
					Non_homologous_end_joining	Genetic Information Processing: Replicatoin and repair
FCGR1	Fc receptor, IgG, high affinity I	102879_s_at	1.3349	0.0079	Fc_gamma_R_mediated_phagocytosis	Organismal Systems: Immune System
					Hematopoietic_cell_lineage	Organismal Systems: Immune System
					Systemic_lupus_erythematosus	Human Diseases: Immune diseases
					Leishmaniasis	Human Diseases: Infectious diseases
					Staphylococcus_aureus_infection	Human Diseases: Infectious diseases

					Tuberculosis	Human Diseases: Infectious diseases
					Phagosome	Cellular Process: Transport and catabolism
					Osteoclast_differentiation	Organismal Systems: Development
					Transcriptional_misregulation_in_cancer	Human Diseases: Cancers
HCK	hemopoietic cell kinase	93483_at	1.4239	0.0146	Fc_gamma_R_mediated_phagocytosis	Organismal Systems: Immune System
					Chemokine_signaling_pathway	Organismal Systems: Immune System
IRF7	interferon regulatory factor 7	104669_at	1.4708	0.0077	Toll_like_receptor_signaling_pathway	Organismal Systems: Immune System
		162202_f_at	1.2999	0.0464	RIG_I_like_receptor_signaling_pathway	Organismal Systems: Immune System
					Cytosolic_DNA_sensing_pathway	Organismal Systems: Immune System
					Hepatitis_B	Human Diseases: Infectious diseases
					Hepatitis_C	Human Diseases: Infectious diseases
					Herpes_simplex_infection	Human Diseases: Infectious diseases
					Influenza_A	Human Diseases: Infectious diseases
					Measles	Human Diseases: Infectious diseases
					Viral_carcinogenesis	Human Diseases: Cancers
ITGB2	integrin beta 2	102353_at	1.4139	0.0120	Leukocyte_transendothelial_migration	Organismal Systems: Immune System
					Natural_killer_cell_mediated_cytotoxicity	Organismal Systems: Immune System
					Amoebiasis	Human Diseases: Infectious diseases
					HTLV_I_infection	Human Diseases: Infectious diseases
					Legionellosis	Human Diseases: Infectious diseases
					Leishmaniasis	Human Diseases: Infectious diseases
					Malaria	Human Diseases: Infectious diseases
					Pertussis	Human Diseases: Infectious diseases
					Cell_adhesion_molecules_(CAMs)	Environmental Information Processing: Signaling molecules and interation
					Hippo_signaling_pathway	Environmental Information Processing: Signal transdution
MYL9	transient receptor protein 2	96939_at	1.3586	0.0500	Leukocyte_transendothelial_migration	Organismal Systems: Immune System
					cAMP_signaling_pathway	Environmental Information Processing: Signal transdution
					cGMP_PKG_signaling_pathway	Environmental Information Processing: Signal transdution
					Focal_adhesion	Cellular Process: Cellular community
					Regulation_of_actin_cytoskeleton	Cellular Process: Cell motility
					Tight_junction	Cellular Process: Cellular community
					Vascular_smooth_muscle_contraction	Organismal Systems: Circulatory system
					Oxytocin_signaling_pathway	Organismal Systems: Endocrine system

PLAUR	urokinase plasminogen activator receptor	102663_at	1.3212	0.0264	Complement_and_coagulation_cascades	Organismal Systems: Immune System
					Proteoglycans_in_cancer	Human Diseases: Cancers
RAC2	RAS-related C3 botulinum substrate 2	103579_at	1.2760	0.0174	Fc_gamma_R_mediated_phagocytosis	Organismal Systems: Immune System
					Fc_epsilon_RI_signaling_pathway	Organismal Systems: Immune System
					Chemokine_signaling_pathway	Organismal Systems: Immune System
					B_cell_receptor_signaling_pathway	Organismal Systems: Immune System
					Axon_guidance	Organismal Systems: Development
					Choline_metabolism_in_cancer	Human Diseases: Cancers
					Colorectal_cancer	Human Diseases: Cancers
					cAMP_signaling_pathway	Environmental Information Processing: Signal transdution
					Adherens_junction	Cellular Process: Cellular community
					Focal_adhesion	Cellular Process: Cellular community
ROCK2	Rho-associated coiled-coil forming kinase 2	98504_at	1.7854	0.0040	Leukocyte_transendothelial_migration	Organismal Systems: Immune System
					Chemokine_signaling_pathway	Organismal Systems: Immune System
					Platelet_activation	Organismal Systems: Immune System
					cAMP_signaling_pathway	Environmental Information Processing: Signal transdution
					cGMP_PKG_signaling_pathway	Environmental Information Processing: Signal transdution
					Focal_adhesion	Cellular Process: Cellular community
					Pathways_in_cancer	Human Diseases: Cancers
					Proteoglycans_in_cancer	Human Diseases: Cancers
					Axon_guidance	Organismal Systems: Development
					Oxytocin_signaling_pathway	Organismal Systems: Endocrine system
VCAM1	vascular cell adhesion molecule 1	92559_at	1.4762	0.0053	Leukocyte_transendothelial_migration	Organismal Systems: Immune System
					NF_kappa_B_signaling_pathway	Environmental Information Processing: Signal transdution
					TNF_signaling_pathway	Environmental Information Processing: Signal transdution
					African_trypanosomiasis	Human Diseases: Infectious diseases
					HTLV_I_infection	Human Diseases: Infectious diseases
					Malaria	Human Diseases: Infectious diseases
					Cell_adhesion_molecules_(CAMs)	Environmental Information Processing: Signaling molecules and interation
VWF	Von Willebrand factor homolog	103499_at	1.2766	0.0354	Complement_and_coagulation_cascades	Organismal Systems: Immune System
					Platelet_activation	Organismal Systems: Immune System
					PI3K_Akt_signaling_pathway	Environmental Information Processing: Signal transdution
					ECM_receptor_interaction	Environmental Information Processing: Signaling molecules and interation
					Focal_adhesion	Cellular Process: Cellular community

The expression of these genes are signficantly upregulated in DEN-treated wild type mouse livers (10-12 month) compared to the age-matched control samples. DAVID analysis coupled with KEGG Pathway indicates they belong to mulitple functional groups but with the theme of "immune system", "immune diseases", and/or inflammation response (in red font). They are often involved in "infectious diseaes" (in blue font), likely due to immune or inflammatory resposne to the pathogens. Many of them are also implicated in cell growth or cancer developmen

(in blue font).

S11 Table. Genes that are involved in amino acid metabolism, and that are down-regulated in WT mice treated with DEN for 10-12 Months

Gene Symbol	Gene name	Probe ID	Fold of Change	p value	Pathways
AADAT	aminoadipate aminotransferase	98123_at	0.483	0.0034	Lysine_biosynthesis<br>Lysine_degradation<br>Tryptophan_metabolism
AASS	lysine oxoglutarate reductase, saccharopine dehydrogenase	103389_at	0.698	0.0154	Lysine_degradation
AGXT	alanine-glyoxylate aminotransferase	93625_at	0.589	0.0343	Alanine,_aspartate_and_glutamate_metabolism<br>Glycine,_serine_and_threonine_metabolism<br>Glyoxylate_and_dicarboxylate_metabolism
AHCY	S-adenosylhomocysteine hydrolase	96025_g_at	0.683	0.0338	Cysteine_and_methionine_metabolism
ALDH2	aldehyde dehydrogenase 2, mitochondrial	96057_at<br>96058_s_at	0.725<br>0.581	0.0247<br>0.0485	Arginine_and_proline_metabolism<br>beta_Alanine_metabolism<br>Histidine_metabolism<br>Lysine_degradation<br>Tryptophan_metabolism<br>Valine,_leucine_and_isoleucine_degradation<br>Ascorbate_and_aldarate_metabolism<br>Glycolysis_/_Gluconeogenesis<br>Pentose_and_glucuronate_interconversions<br>Pyruvate_metabolism<br>Fatty_acid_degradation<br>Glycerolipid_metabolism
ALDH7A1	aldehyde dehydrogenase 7 family member A1	97449_at<br>97450_s_at	0.669<br>0.616	0.0263<br>0.0438	Arginine_and_proline_metabolism<br>beta_Alanine_metabolism<br>Glycine,_serine_and_threonine_metabolism<br>Histidine_metabolism<br>Lysine_biosynthesis<br>Lysine_degradation<br>Tryptophan_metabolism<br>Valine,_leucine_and_isoleucine_degradation<br>Ascorbate_and_aldarate_metabolism<br>Glycolysis_/_Gluconeogenesis<br>Pyruvate_metabolism<br>Fatty_acid_degradation

					Glycerolipid_metabolism
AUH	AU RNA binding protein/enoyl-coenzyme A hydratase	96650_at	0.794	0.0044	Valine,_leucine_and_isoleucine_degradation
BCKDHA	branched chain ketoacid dehydrogenase E1, alpha polypeptide	96035_at	0.695	0.0112	Valine,_leucine_and_isoleucine_degradation
DMGDH	dimethylglycine dehydrogenase	104086_at	0.503	0.0018	Glycine,_serine_and_threonine_metabolism
ECHS1	enoyl Coenzyme A hydratase, short chain, 1, mitochondrial	95426_at	0.664	0.0062	beta_Alanine_metabolism<br>Lysine_degradation<br>Tryptophan_metabolism<br>Valine,_leucine_and_isoleucine_degradation<br>Butanoate_metabolism<br>Propanoate_metabolism<br>Fatty_acid_degradation<br>Fatty_acid_elongation
FAH	fumarylacetoacetate hydrolase	98588_at	0.756	0.0262	Tyrosine_metabolism
HIBADH	3-hydroxyisobutyrate dehydrogenase	97279_at	0.475	0.0115	Valine,_leucine_and_isoleucine_degradation
IVD	isovaleryl coenzyme A dehydrogenase	104153_at	0.667	0.0115	Valine,_leucine_and_isoleucine_degradation
LAP3	leucine amionpeptidase 3	98112_r_at	0.559	0.0127	Arginine_and_proline_metabolism<br>Glutathione_metabolism
MCCC1	methylcrotonoyl-Coenzyme A carboxylase 1 (alpha)	94940_at	0.675	0.0136	Valine,_leucine_and_isoleucine_degradation
NIT2	Nitrilase Family, Member 2	160135_at	0.628	0.0116	Alanine,_aspartate_and_glutamate_metabolism
OTC	ornithine transcarbamylase	94414_at	0.528	0.0260	Arginine_and_proline_metabolism
PAH	phenylalanine hydroxylase	95407_at	0.572	0.0371	Phenylalanine,_tyrosine_and_tryptophan_biosynthesis<br>Phenylalanine_metabolism
PIPOX	peroxisomal sarcosine oxidase	101844_at	0.588	0.0012	Glycine,_serine_and_threonine_metabolism<br>Lysine_degradation
PRODH2	proline oxidase 1	103452_at	0.714	0.0454	Arginine_and_proline_metabolism

SARDH	sarcosine dehydrogenase	96763_at	0.473	0.0003	Glycine,_serine_and_threonine_metabolism

The expression of these genes are signficantly downregulated in DEN-treated wild type mouse livers (10-12 month) compared to the age-matched control samples. DAVID analysis coupled with KEGG Pathway indicates they belong to the functional group of "Metabolism: amino acid metabolism", and "Metabolism: Metabolism of other amino acids". Some of the genes also have functions in carbohydrate metabolism (in blue font) and/or lipid metabolism (in green font)
