# Supplementary material for: Gene Expression Analysis Indicates Divergent Mechanisms in DEN-Induced Carcinogenesis in Wild Type and Bid-Deficient Livers
Source: PLoS One. 2016 May 19;11(5):e0155211. doi: 10.1371/journal.pone.0155211 (PMC4873180; doi:10.1371/journal.pone.0155211)
Supplement: S10 Table — (PDF) [file pone.0155211.s010.pdf]

S10 Table. Genes that are involved in immune response and/or inflammation, and that are upregulated in WT mice treated with DEN for 10-12 Months

Gene Symbol	Gene Name	Probe ID	Fold of Change	p value	Pathways	Functional Class
C1QA	complement component 1, q subcomponent, alpha polypeptide	98562_at	1.6453	0.0269	Complement_and_coagulation_cascades	Organismal Systems: Immune System
					Systemic_lupus_erythematosus	Human Diseases: Immune diseases
					Chagas_disease_(American_trypanosomiasis)	Human Diseases: Infectious diseases
					Pertussis	Human Diseases: Infectious diseases
					Staphylococcus_aureus_infection	Human Diseases: Infectious diseases
					Prion_diseases	Human Diseases: Neurodegenerative diseases
C1QB	complement component 1, q subcomponent, beta polypeptide	96020_at	2.0625	0.0271	Complement_and_coagulation_cascades	Organismal Systems: Immune System
					Systemic_lupus_erythematosus	Human Diseases: Immune diseases
					Chagas_disease_(American_trypanosomiasis)	Human Diseases: Infectious diseases
					Pertussis	Human Diseases: Infectious diseases
					Staphylococcus_aureus_infection	Human Diseases: Infectious diseases
					Prion_diseases	Human Diseases: Neurodegenerative diseases
C1QC	complement component 1, q subcomponent, c polypeptide	92223_at	1.7362	0.0065	Complement_and_coagulation_cascades	Organismal Systems: Immune System
					Systemic_lupus_erythematosus	Human Diseases: Immune diseases
					Chagas_disease_(American_trypanosomiasis)	Human Diseases: Infectious diseases
					Pertussis	Human Diseases: Infectious diseases
					Staphylococcus_aureus_infection	Human Diseases: Infectious diseases
					Prion_diseases	Human Diseases: Neurodegenerative diseases
CCL4	small inducible cytokine A4	94146_at	1.2846	0.0351	Toll_like_receptor_signaling_pathway	Organismal Systems: Immune System
					Chemokine_signaling_pathway	Organismal Systems: Immune System
					Cytosolic_DNA_sensing_pathway	Organismal Systems: Immune System
					Cytokine_cytokine_receptor_interaction	Environmental Information Processing: Signaling molecules and interation
					NF_kappa_B_signaling_pathway	Environmental Information Processing: Signal transdution
					Salmonella_infection	Human Diseases: Infectious diseases
CCL5	small inducible cytokine A5	98406_at	1.4894	0.0475	TNF_signaling_pathway	Environmental Information Processing: Signal transdution
					Cytokine_cytokine_receptor_interaction	Environmental Information Processing: Signaling molecules and interation
					Chemokine_signaling_pathway	Organismal Systems: Immune System

					NOD_like_receptor_signaling_pathway	Organismal Systems: Immune System
					Cytosolic_DNA_sensing_pathway	Organismal Systems: Immune System
					Rheumatoid_arthritis	Human Diseases: Immune diseases
					Herpes_simplex_infection	Human Diseases: Infectious diseases
					Influenza_A	Human Diseases: Infectious diseases
					Chagas_disease_(American_trypanosomiasis)	Human Diseases: Infectious diseases
					Prion_diseases	Human Diseases: Neurodegenerative diseases
CD14	CD14 antigen	98088_at	2.0174	0.0178	Hematopoietic_cell_lineage	Organismal Systems: Immune System
					Toll_like_receptor_signaling_pathway	Organismal Systems: Immune System
					NF_kappa_B_signaling_pathway	Environmental Information Processing: Signal transdution
					Amoebiasis	Human Diseases: Infectious diseases
					Legionellosis	Human Diseases: Infectious diseases
					Pertussis	Human Diseases: Infectious diseases
					Salmonella_infection	Human Diseases: Infectious diseases
					MAPK_signaling_pathway	Environmental Information Processing: Signal transdution
					Phagosome	Cellular Process: Transport and catabolism
					Regulation_of_actin_cytoskeleton	Cellular Process: Cell motility
CD99	CD99 antigen	101047_at	1.6316	0.0426	Leukocyte_transendothelial_migration	Organismal Systems: Immune System
					Cell_adhesion_molecules_(CAMs)	Environmental Information Processing: Signaling molecules and interation
CYBA	cytochrome b-245, alpha polypeptide	100059_at;	1.4238	0.0014	Leukocyte_transendothelial_migration	Organismal Systems: Immune System
		97013_f_at	1.5608	0.0332	Leishmaniasis	Human Diseases: Infectious diseases
					Osteoclast_differentiation	Organismal Systems: Development
					Phagosome	Cellular Process: Transport and catabolism
DNTT	deoxynucleotidyltransferase, terminal	103962_at	1.2917	0.0254	Hematopoietic_cell_lineage	Organismal Systems: Immune System
					Non_homologous_end_joining	Genetic Information Processing: Replicatoin and repair
FCGR1	Fc receptor, IgG, high affinity I	102879_s_at	1.3349	0.0079	Fc_gamma_R_mediated_phagocytosis	Organismal Systems: Immune System
					Hematopoietic_cell_lineage	Organismal Systems: Immune System
					Systemic_lupus_erythematosus	Human Diseases: Immune diseases
					Leishmaniasis	Human Diseases: Infectious diseases
					Staphylococcus_aureus_infection	Human Diseases: Infectious diseases

					Tuberculosis	Human Diseases: Infectious diseases
					Phagosome	Cellular Process: Transport and catabolism
					Osteoclast_differentiation	Organismal Systems: Development
					Transcriptional_misregulation_in_cancer	Human Diseases: Cancers
HCK	hemopoietic cell kinase	93483_at	1.4239	0.0146	Fc_gamma_R_mediated_phagocytosis	Organismal Systems: Immune System
					Chemokine_signaling_pathway	Organismal Systems: Immune System
IRF7	interferon regulatory factor 7	104669_at	1.4708	0.0077	Toll_like_receptor_signaling_pathway	Organismal Systems: Immune System
		162202_f_at	1.2999	0.0464	RIG_I_like_receptor_signaling_pathway	Organismal Systems: Immune System
					Cytosolic_DNA_sensing_pathway	Organismal Systems: Immune System
					Hepatitis_B	Human Diseases: Infectious diseases
					Hepatitis_C	Human Diseases: Infectious diseases
					Herpes_simplex_infection	Human Diseases: Infectious diseases
					Influenza_A	Human Diseases: Infectious diseases
					Measles	Human Diseases: Infectious diseases
					Viral_carcinogenesis	Human Diseases: Cancers
ITGB2	integrin beta 2	102353_at	1.4139	0.0120	Leukocyte_transendothelial_migration	Organismal Systems: Immune System
					Natural_killer_cell_mediated_cytotoxicity	Organismal Systems: Immune System
					Amoebiasis	Human Diseases: Infectious diseases
					HTLV_I_infection	Human Diseases: Infectious diseases
					Legionellosis	Human Diseases: Infectious diseases
					Leishmaniasis	Human Diseases: Infectious diseases
					Malaria	Human Diseases: Infectious diseases
					Pertussis	Human Diseases: Infectious diseases
					Cell_adhesion_molecules_(CAMs)	Environmental Information Processing: Signaling molecules and interation
					Hippo_signaling_pathway	Environmental Information Processing: Signal transdution
MYL9	transient receptor protein 2	96939_at	1.3586	0.0500	Leukocyte_transendothelial_migration	Organismal Systems: Immune System
					cAMP_signaling_pathway	Environmental Information Processing: Signal transdution
					cGMP_PKG_signaling_pathway	Environmental Information Processing: Signal transdution
					Focal_adhesion	Cellular Process: Cellular community
					Regulation_of_actin_cytoskeleton	Cellular Process: Cell motility
					Tight_junction	Cellular Process: Cellular community
					Vascular_smooth_muscle_contraction	Organismal Systems: Circulatory system
					Oxytocin_signaling_pathway	Organismal Systems: Endocrine system

PLAUR	urokinase plasminogen activator receptor	102663_at	1.3212	0.0264	Complement_and_coagulation_cascades	Organismal Systems: Immune System
					Proteoglycans_in_cancer	Human Diseases: Cancers
RAC2	RAS-related C3 botulinum substrate 2	103579_at	1.2760	0.0174	Fc_gamma_R_mediated_phagocytosis	Organismal Systems: Immune System
					Fc_epsilon_RI_signaling_pathway	Organismal Systems: Immune System
					Chemokine_signaling_pathway	Organismal Systems: Immune System
					B_cell_receptor_signaling_pathway	Organismal Systems: Immune System
					Axon_guidance	Organismal Systems: Development
					Choline_metabolism_in_cancer	Human Diseases: Cancers
					Colorectal_cancer	Human Diseases: Cancers
					cAMP_signaling_pathway	Environmental Information Processing: Signal transdution
					Adherens_junction	Cellular Process: Cellular community
					Focal_adhesion	Cellular Process: Cellular community
ROCK2	Rho-associated coiled-coil forming kinase 2	98504_at	1.7854	0.0040	Leukocyte_transendothelial_migration	Organismal Systems: Immune System
					Chemokine_signaling_pathway	Organismal Systems: Immune System
					Platelet_activation	Organismal Systems: Immune System
					cAMP_signaling_pathway	Environmental Information Processing: Signal transdution
					cGMP_PKG_signaling_pathway	Environmental Information Processing: Signal transdution
					Focal_adhesion	Cellular Process: Cellular community
					Pathways_in_cancer	Human Diseases: Cancers
					Proteoglycans_in_cancer	Human Diseases: Cancers
					Axon_guidance	Organismal Systems: Development
					Oxytocin_signaling_pathway	Organismal Systems: Endocrine system
VCAM1	vascular cell adhesion molecule 1	92559_at	1.4762	0.0053	Leukocyte_transendothelial_migration	Organismal Systems: Immune System
					NF_kappa_B_signaling_pathway	Environmental Information Processing: Signal transdution
					TNF_signaling_pathway	Environmental Information Processing: Signal transdution
					African_trypanosomiasis	Human Diseases: Infectious diseases
					HTLV_I_infection	Human Diseases: Infectious diseases
					Malaria	Human Diseases: Infectious diseases
					Cell_adhesion_molecules_(CAMs)	Environmental Information Processing: Signaling molecules and interation
VWF	Von Willebrand factor homolog	103499_at	1.2766	0.0354	Complement_and_coagulation_cascades	Organismal Systems: Immune System
					Platelet_activation	Organismal Systems: Immune System
					PI3K_Akt_signaling_pathway	Environmental Information Processing: Signal transdution
					ECM_receptor_interaction	Environmental Information Processing: Signaling molecules and interation
					Focal_adhesion	Cellular Process: Cellular community

The expression of these genes are signficantly upregulated in DEN-treated wild type mouse livers (10-12 month) compared to the age-matched control samples. DAVID analysis coupled with KEGG Pathway indicates they belong to mulitple functional groups but with the theme of "immune system", "immune diseases", and/or inflammation response (in red font). They are often involved in "infectious diseaes" (in blue font), likely due to immune or inflammatory resposne to the pathogens. Many of them are also implicated in cell growth or cancer development

(in blue font).
