# Supplementary material for: Gene Expression Analysis Indicates Divergent Mechanisms in DEN-Induced Carcinogenesis in Wild Type and Bid-Deficient Livers
Source: PLoS One. 2016 May 19;11(5):e0155211. doi: 10.1371/journal.pone.0155211 (PMC4873180; doi:10.1371/journal.pone.0155211)
Supplement: S11 Table — (PDF) [file pone.0155211.s011.pdf]

S11 Table. Genes that are involved in amino acid metabolism, and that are down-regulated in WT mice treated with DEN for 10-12 Months

Gene Symbol	Gene name	Probe ID	Fold of Change	p value	Pathways
AADAT	aminoadipate aminotransferase	98123_at	0.483	0.0034	Lysine_biosynthesis<br>Lysine_degradation<br>Tryptophan_metabolism
AASS	lysine oxoglutarate reductase, saccharopine dehydrogenase	103389_at	0.698	0.0154	Lysine_degradation
AGXT	alanine-glyoxylate aminotransferase	93625_at	0.589	0.0343	Alanine,_aspartate_and_glutamate_metabolism<br>Glycine,_serine_and_threonine_metabolism<br>Glyoxylate_and_dicarboxylate_metabolism
AHCY	S-adenosylhomocysteine hydrolase	96025_g_at	0.683	0.0338	Cysteine_and_methionine_metabolism
ALDH2	aldehyde dehydrogenase 2, mitochondrial	96057_at	0.725	0.0247	Arginine_and_proline_metabolism
		96058_s_at	0.581	0.0485	beta_Alanine_metabolism<br>Histidine_metabolism<br>Lysine_degradation<br>Tryptophan_metabolism<br>Valine,_leucine_and_isoleucine_degradation<br>Ascorbate_and_aldarate_metabolism<br>Glycolysis_/_Gluconeogenesis<br>Pentose_and_glucuronate_interconversions<br>Pyruvate_metabolism<br>Fatty_acid_degradation<br>Glycerolipid_metabolism
ALDH7A1	aldehyde dehydrogenase 7 family member A1	97449_at	0.669	0.0263	Arginine_and_proline_metabolism
		97450_s_at	0.616	0.0438	beta_Alanine_metabolism<br>Glycine,_serine_and_threonine_metabolism<br>Histidine_metabolism<br>Lysine_biosynthesis<br>Lysine_degradation<br>Tryptophan_metabolism<br>Valine,_leucine_and_isoleucine_degradation<br>Ascorbate_and_aldarate_metabolism<br>Glycolysis_/_Gluconeogenesis<br>Pyruvate_metabolism<br>Fatty_acid_degradation

					Glycerolipid_metabolism
AUH	AU RNA binding protein/enoyl-coenzyme A hydratase	96650_at	0.794	0.0044	Valine,_leucine_and_isoleucine_degradation
BCKDHA	branched chain ketoacid dehydrogenase E1, alpha polypeptide	96035_at	0.695	0.0112	Valine,_leucine_and_isoleucine_degradation
DMGDH	dimethylglycine dehydrogenase	104086_at	0.503	0.0018	Glycine,_serine_and_threonine_metabolism
ECHS1	enoyl Coenzyme A hydratase, short chain, 1, mitochondrial	95426_at	0.664	0.0062	beta_Alanine_metabolism<br>Lysine_degradation<br>Tryptophan_metabolism<br>Valine,_leucine_and_isoleucine_degradation<br>Butanoate_metabolism<br>Propanoate_metabolism<br>Fatty_acid_degradation<br>Fatty_acid_elongation
FAH	fumarylacetoacetate hydrolase	98588_at	0.756	0.0262	Tyrosine_metabolism
HIBADH	3-hydroxyisobutyrate dehydrogenase	97279_at	0.475	0.0115	Valine,_leucine_and_isoleucine_degradation
IVD	isovaleryl coenzyme A dehydrogenase	104153_at	0.667	0.0115	Valine,_leucine_and_isoleucine_degradation
LAP3	leucine amionpeptidase 3	98112_r_at	0.559	0.0127	Arginine_and_proline_metabolism<br>Glutathione_metabolism
MCCC1	methylcrotonoyl-Coenzyme A carboxylase 1 (alpha)	94940_at	0.675	0.0136	Valine,_leucine_and_isoleucine_degradation
NIT2	Nitrilase Family, Member 2	160135_at	0.628	0.0116	Alanine,_aspartate_and_glutamate_metabolism
OTC	ornithine transcarbamylase	94414_at	0.528	0.0260	Arginine_and_proline_metabolism
PAH	phenylalanine hydroxylase	95407_at	0.572	0.0371	Phenylalanine,_tyrosine_and_tryptophan_biosynthesis<br>Phenylalanine_metabolism
PIPOX	peroxisomal sarcosine oxidase	101844_at	0.588	0.0012	Glycine,_serine_and_threonine_metabolism<br>Lysine_degradation
PRODH2	proline oxidase 1	103452_at	0.714	0.0454	Arginine_and_proline_metabolism

SARDH	sarcosine dehydrogenase	96763_at	0.473	0.0003	Glycine,_serine_and_threonine_metabolism

The expression of these genes are signficantly downregulated in DEN-treated wild type mouse livers (10-12 month) compared to the age-matched control samples. DAVID analysis coupled with KEGG Pathway indicates they belong to the functional group of "Metabolism: amino acid metabolism", and "Metabolism: Metabolism of other amino acids". Some of the genes also have functions in carbohydrate metabolism (in blue font) and/or lipid metabolism (in green font)
