# Supplementary material for: Gene Expression Analysis Indicates Divergent Mechanisms in DEN-Induced Carcinogenesis in Wild Type and Bid-Deficient Livers
Source: PLoS One. 2016 May 19;11(5):e0155211. doi: 10.1371/journal.pone.0155211 (PMC4873180; doi:10.1371/journal.pone.0155211)
Supplement: S12 Table — (PDF) [file pone.0155211.s012.pdf]

S12 Table. Pathway analysis of the gene expression profile in wild type mice treated with DEN for 4-6 months

Class	Pathways	Size	Change	ES	NES	NOM p-val	FDR q-val	FWER p-val	RANK AT MAX
Cellular Processes; Cellular commiunity	Signaling_pathways_regulating_pluripotency_of_stem_cells	113	↑	0.3615	1.354	0.0365	0.3226	1	2084
Environmental Information Processing; Signal transduction	Hippo_signaling_pathway	119	↑	0.3486	1.3249	0.0495	0.3514	1	3076
Environmental Information Processing; Signal transduction	Jak_STAT_signaling_pathway	101	↑	0.3942	1.4516	0.0143	0.3344	1	3826
Environmental Information Processing; Signal transduction	MAPK_signaling_pathway	182	↑	0.3992	1.5723	0	0.2393	0.927	2200
Environmental Information Processing; Signal transduction	NF_kappa_B_signaling_pathway	63	↑	0.418	1.4447	0.0289	0.3226	1	2350
Environmental Information Processing; Signal transduction	TNF_signaling_pathway	81	↑	0.4675	1.6721	0	0.3389	0.521	2350
Environmental Information Processing; Signal transduction	Wnt_signaling_pathway	101	↑	0.3787	1.4162	0.0286	0.3194	1	3054
Environmental Information Processing; Signaling molecules and interaction	Cytokine_cytokine_receptor_interaction	173	↑	0.3645	1.4378	0.0042	0.3264	1	2577
Environmental Information Processing; Signaling molecules and interaction	Neuroactive_ligand_receptor_interaction	165	↑	0.395	1.5369	0.0021	0.2364	0.973	2721
Genetic Information Processing; Folding, sorting and degradation	Protein_processing_in_endoplasmic_reticulum	114	↑	0.398	1.4844	0.0089	0.2821	0.998	574
Human Diseases; Cancers	Basal_cell_carcinoma	43	↑	0.4471	1.4452	0.0388	0.3366	1	3054
Human Diseases; Cancers	Pathways_in_cancer	306	↑	0.3395	1.392	0.0051	0.3308	1	3073
Human Diseases; Cardiovascular diseases	Hypertrophic_cardiomyopathy_(HCM)	57	↑	0.4566	1.5587	0.0073	0.2272	0.942	2359
Human Diseases; Cardiovascular diseases	Viral_myocarditis	57	↑	0.4304	1.4853	0.0177	0.2963	0.998	1948
Human Diseases; Immune diseases	Allograft_rejection	36	↑	0.5177	1.645	0.0026	0.1911	0.653	1897
Human Diseases; Immune diseases	Asthma	23	↑	0.6615	1.8823	0.0027	0.0309	0.035	1175
Human Diseases; Immune diseases	Autoimmune_thyroid_disease	44	↑	0.5054	1.6397	0.0065	0.1675	0.675	1910
Human Diseases; Infectious diseases	Chagas_disease_(American_trypanosomiasis)	83	↑	0.4475	1.6113	0.0012	0.172	0.795	2354
Human Diseases; Infectious diseases	Hepatitis_B	106	↑	0.3709	1.3779	0.0319	0.3379	1	3450
Human Diseases; Infectious diseases	Herpes_simplex_infection	123	↑	0.3822	1.4367	0.0167	0.3156	1	3037
Human Diseases; Infectious diseases	HTLV_I_infection	211	↑	0.381	1.5172	0.0021	0.2322	0.988	3054
Human Diseases; Infectious diseases	Influenza_A	116	↑	0.4244	1.6171	0.0022	0.1848	0.774	2403
Human Diseases; Infectious diseases	Malaria	38	↑	0.5276	1.6673	0.0089	0.2433	0.555	588
Human Diseases; Infectious diseases	Measles	92	↑	0.4077	1.5196	0.0115	0.2417	0.987	2386

Human Diseases; Infectious diseases	Toxoplasmosis	88	↑	0.3705	1.3546	0.0483	0.3286	1	2403
Organismal Systems; Development	Osteoclast_differentiation	96	↑	0.3871	1.4208	0.0289	0.3314	1	2253
Organismal Systems; Endocrine system	Renin_angiotensin_system	13	↑	0.6105	1.5267	0.0408	0.2424	0.982	2338
Organismal Systems; Immune system	Antigen_processing_and_presentation	55	↑	0.4657	1.571	0.0109	0.2191	0.929	1780
Organismal Systems; Immune system	Chemokine_signaling_pathway	130	↑	0.3585	1.3619	0.0328	0.3344	1	2577
Organismal Systems; Immune system	Cytosolic_DNA_sensing_pathway	36	↑	0.462	1.4605	0.0429	0.3265	1	2629
Organismal Systems; Immune system	Leukocyte_transendothelial_migration	83	↑	0.431	1.5528	0.0058	0.2194	0.952	1287
Organismal Systems; Immune system	NOD_like_receptor_signaling_pathway	37	↑	0.5241	1.6587	0.0078	0.2036	0.597	1753
Organismal Systems; Immune system	Toll_like_receptor_signaling_pathway	74	↑	0.387	1.3797	0.0417	0.3425	1	3427
Cellular Processes; Transport and catabolism	Peroxisome	49	↓	-0.4221	-1.7847	0	0.0455	0.248	1761
Genetic Information Processing; Folding, sorting and degradation	Proteasome	40	↓	-0.4647	-1.8343	0	0.0379	0.172	2234
Genetic Information Processing; Replication and repair	Nucleotide_excision_repair	34	↓	-0.449	-1.7251	0.0091	0.0613	0.373	1677
Genetic Information Processing; Translation	Ribosome	89	↓	-0.4184	-1.9344	0	0.034	0.068	2144
Human Diseases; Cancers	Chemical_carcinogenesis	48	↓	-0.3691	-1.5237	0.0105	0.1625	0.833	1568
Human Diseases; Neurodegenerative diseases	Alzheimer's_disease	127	↓	-0.2665	-1.3076	0.0426	0.3459	1	1772
Human Diseases; Neurodegenerative diseases	Huntington's_disease	123	↓	-0.3579	-1.7481	0	0.0559	0.325	1852
Human Diseases; Neurodegenerative diseases	Parkinson's_disease	95	↓	-0.3538	-1.6546	0	0.0842	0.538	1772
Metabolism; Amino acid metabolism	Valine,_leucine_and_isoleucine_degradation	33	↓	-0.6365	-2.4262	0	0	0	1624
Metabolism; Biosynthesis of other secondary metabolites	Caffeine_metabolism	5	↓	-0.7956	-1.6793	0.013	0.0757	0.473	1121
Metabolism; Carbohydrate metabolism	Propanoate_metabolism	17	↓	-0.5877	-1.9027	0.0035	0.033	0.089	1854
Metabolism; Energy metabolism	Oxidative_phosphorylation	88	↓	-0.4602	-2.1523	0	0.0044	0.006	1772
Metabolism; Lipid metabolism	Biosynthesis_of_unsaturated_fatty_acids	11	↓	-0.6365	-1.792	0.0058	0.0463	0.224	1770
Metabolism; Lipid metabolism	Fatty_acid_degradation	27	↓	-0.4325	-1.5552	0.0326	0.1403	0.772	1563
Metabolism; Lipid metabolism	Primary_bile_acid_biosynthesis	11	↓	-0.5735	-1.6159	0.0356	0.1029	0.643	1555
Metabolism; Lipid metabolism	Steroid_biosynthesis	14	↓	-0.631	-1.8541	0	0.0359	0.143	1137
Metabolism; Metabolism of terpenoids and polyketides	Terpenoid_backbone_biosynthesis	14	↓	-0.6398	-1.8867	0	0.0309	0.103	417

Metabolism; Xenobiotics biodegradation and metabolism	Drug_metabolism_cytochrome_P450	29	↓	-0.4173	-1.5058	0.041	0.1739	0.869	1568

1. The complete gene expression profile in DEN-treated *bid* -deficient livers for 4-6 months were subjected to GSEA using KEGG pathway desgination.
2. Size: Number of genes in the gene set after filtering out these genes not in the expression dataset.
3. ES: Enrichment score for the gene set, the degree to which this gene set is overrepresented at the top or bottom of the ranked list of genes in the expression dataset.
4. NES: Normalized enrichment score, the enrichment score for the gene set after it has been normalized across analyzed gene sets.
5. NOR p-value: Norminal p value, the statistical significance of the enrichment score. It is not adjusted for gene set size or multiple hypothesis testing.
6. FRD q-value: False discovery rate, the estimated probability that the nromalized enrichment score representes a false positive finding.
7. FWER p-value: Familywise-error rate, a more conservatively estimated probabillity that the normalzied enrichment score represents a false positive findnig.
8. RANK AT MAX: The positiion in the ranked list at which the maximum enrichment socre occurred.
9. For the up-regulated pathways, those related to immune response/inflammation are shown in red font, and those related to cancer and growth regulation are shown in blue font.

For the down-regulated pathways, those related to amino acids metabolism are shown in red font, and those related to other types of metabolisms are shown in blue font.
