# Supplementary material for: Gene Expression Analysis Indicates Divergent Mechanisms in DEN-Induced Carcinogenesis in Wild Type and Bid-Deficient Livers
Source: PLoS One. 2016 May 19;11(5):e0155211. doi: 10.1371/journal.pone.0155211 (PMC4873180; doi:10.1371/journal.pone.0155211)
Supplement: S13 Table — (PDF) [file pone.0155211.s013.pdf]

S13 Table. Pathway analysis of the gene expression profile in wild type mice treated with DEN for 10-12 months

Class	Pathways	Size	Change	ES	NES	NOM p-val	FDR q-val	FWER p-val	RANK AT MAX
Cellular Processes; Cell growth and death	Cell_cycle	85	↑	0.369	1.4383	0.0123	0.1406	0.998	1977
Cellular Processes; Cell motility	Regulation_of_actin_cytoskeleton	154	↑	0.3709	1.5897	0.002	0.0631	0.864	1219
Cellular Processes; Cellular community	Focal_adhesion	143	↑	0.4182	1.7641	0.0022	0.0285	0.336	1053
Cellular Processes; Transport and catabolism	Phagosome	107	↑	0.4474	1.8477	0	0.0156	0.142	640
Environmental Information Processing; Membrane transport	ABC_transporters	26	↑	0.4999	1.4943	0.0442	0.1088	0.985	305
Environmental Information Processing; Signal transduction	cAMP_signaling_pathway	128	↑	0.3235	1.3429	0.0377	0.2105	1	2071
Environmental Information Processing; Signal transduction	Hippo_signaling_pathway	119	↑	0.3613	1.469	0.0103	0.1234	0.994	2385
Environmental Information Processing; Signal transduction	Jak_STAT_signaling_pathway	101	↑	0.3632	1.4494	0.0208	0.1373	0.998	2526
Environmental Information Processing; Signal transduction	MAPK_signaling_pathway	182	↑	0.3513	1.5363	0	0.082	0.953	2489
Environmental Information Processing; Signal transduction	NF_kappa_B_signaling_pathway	63	↑	0.4621	1.6878	0.006	0.0433	0.59	1217
Environmental Information Processing; Signal transduction	TGF_beta_signaling_pathway	64	↑	0.3839	1.4177	0.0391	0.1532	1	1936
Environmental Information Processing; Signal transduction	TNF_signaling_pathway	81	↑	0.5365	2.0333	0	0.0026	0.006	971
Environmental Information Processing; Signal transduction	Wnt_signaling_pathway	101	↑	0.3609	1.4342	0.0211	0.1418	0.998	1150
Environmental Information Processing; Signaling molecules and interaction	Cell_adhesion_molecules_(CAMs)	93	↑	0.4457	1.7465	0	0.0331	0.39	1571
Environmental Information Processing; Signaling molecules and interaction	Cytokine_cytokine_receptor_ interaction	173	↑	0.4725	2.0204	0	0.0023	0.007	2481
Environmental Information Processing; Signaling molecules and interaction	ECM_receptor_interaction	58	↑	0.4241	1.5372	0.0122	0.083	0.952	2109
Environmental Information Processing; Signaling molecules and interaction	Neuroactive_ligand_receptor_ interaction	165	↑	0.3374	1.4486	0.0022	0.1356	0.998	3332

Human Diseases; Cancers	Basal_cell_carcinoma	43	↑	0.4667	1.5783	0.0162	0.0657	0.889	3228
Human Diseases; Cancers	Colorectal_cancer	52	↑	0.4582	1.6354	0.0022	0.0503	0.747	1150
Human Diseases; Cancers	MicroRNAs_in_cancer	110	↑	0.3444	1.3955	0.0277	0.1701	1	1223
Human Diseases; Cancers	Pancreatic_cancer	55	↑	0.3852	1.3925	0.0361	0.1704	1	1686
Human Diseases; Cancers	Pathways_in_cancer	306	↑	0.3556	1.6436	0	0.0513	0.726	2140
Human Diseases; Cancers	Proteoglycans_in_cancer	155	↑	0.3582	1.5341	0.0043	0.0819	0.955	1206
Human Diseases; Cancers	Small_cell_lung_cancer	64	↑	0.4507	1.6761	0.0023	0.0444	0.625	1223
Human Diseases; Cancers	Transcriptional_misregulation_in_cancer	132	↑	0.3888	1.6141	0.0021	0.0583	0.799	1599
Human Diseases; Cardiovascular diseases	Viral_myocarditis	57	↑	0.5316	1.9373	0	0.0065	0.034	1486
Human Diseases; Endocrine and metabolic diseases	Type_I_diabetes_mellitus	41	↑	0.5121	1.7416	0.002	0.0327	0.404	2305
Human Diseases; Immune diseases	Allograft_rejection	36	↑	0.5258	1.7203	0.0107	0.0379	0.469	1499
Human Diseases; Immune diseases	Asthma	23	↑	0.5777	1.685	0.0083	0.0427	0.601	2678
Human Diseases; Immune diseases	Autoimmune_thyroid_disease	44	↑	0.4796	1.607	0.009	0.0589	0.821	2313
Human Diseases; Immune diseases	Graft_versus_host_disease	36	↑	0.5934	1.9523	0	0.0063	0.028	1713
Human Diseases; Immune diseases	Inflammatory_bowel_disease_(IBD)	45	↑	0.4838	1.6641	0.0104	0.0447	0.668	2678
Human Diseases; Immune diseases	Rheumatoid_arthritis	64	↑	0.4427	1.6382	0.0083	0.0519	0.739	1588
Human Diseases; Infectious diseases	African_trypanosomiasis	24	↑	0.5538	1.6737	0.0125	0.0439	0.631	1336
Human Diseases; Infectious diseases	Amoebiasis	78	↑	0.4072	1.5529	0.0219	0.0771	0.936	1417
Human Diseases; Infectious diseases	Chagas_disease_(American_ trypanosomiasis)	83	↑	0.4785	1.8617	0	0.0149	0.115	1985

Human Diseases; Infectious diseases	Hepatitis_B	106	↑	0.3621	1.463	0.0191	0.1271	0.994	1382
Human Diseases; Infectious diseases	Herpes_simplex_infection	123	↑	0.4309	1.7743	0	0.0284	0.306	1250
Human Diseases; Infectious diseases	HTLV_I_infection	211	↑	0.4034	1.8069	0	0.0203	0.22	2254
Human Diseases; Infectious diseases	Influenza_A	116	↑	0.4599	1.8747	0	0.0163	0.102	1985
Human Diseases; Infectious diseases	Legionellosis	42	↑	0.5141	1.7063	0.0089	0.042	0.516	1076
Human Diseases; Infectious diseases	Leishmaniasis	54	↑	0.5143	1.873	0	0.015	0.105	2080
Human Diseases; Infectious diseases	Malaria	38	↑	0.5128	1.6907	0.004	0.0437	0.577	1531
Human Diseases; Infectious diseases	Measles	92	↑	0.3629	1.421	0.0164	0.1524	0.999	2135
Human Diseases; Infectious diseases	Pertussis	53	↑	0.4447	1.6022	0.0103	0.06	0.834	1614
Human Diseases; Infectious diseases	Salmonella_infection	56	↑	0.5516	1.993	0	0.0034	0.013	1614
Human Diseases; Infectious diseases	Staphylococcus_aureus_infection	36	↑	0.5586	1.836	0	0.0175	0.168	586
Human Diseases; Infectious diseases	Toxoplasmosis	88	↑	0.3808	1.4896	0.015	0.11	0.987	3151
Human Diseases; Infectious diseases	Tuberculosis	131	↑	0.3815	1.5952	0	0.0616	0.85	1780
Human Diseases; Neurodegenerative diseases	Prion_diseases	28	↑	0.5068	1.5684	0.024	0.0693	0.909	402
Metabolism; Carbohydrate metabolism	Fructose_and_mannose_ metabolism	24	↑	0.5352	1.6116	0.0122	0.0577	0.804	1439
Organismal Systems; Development	Osteoclast_differentiation	96	↑	0.5207	2.0536	0	0.0026	0.004	1615
Organismal Systems; Digestive system	Protein_digestion_and_absorption	54	↑	0.4548	1.6374	0.0022	0.0507	0.741	2867
Organismal Systems; Endocrine system	Oxytocin_signaling_pathway	99	↑	0.3665	1.4446	0.0278	0.1371	0.998	980
Organismal Systems; Endocrine system	Prolactin_signaling_pathway	60	↑	0.3768	1.3813	0.046	0.1807	1	1780

Organismal Systems; Immune system	Antigen_processing_and_ presentation	55	↑	0.5294	1.8864	0	0.0156	0.089	1486
Organismal Systems; Immune system	B_cell_receptor_signaling_ pathway	55	↑	0.433	1.5815	0.0065	0.0655	0.883	940
Organismal Systems; Immune system	Chemokine_signaling_pathway	130	↑	0.4506	1.8499	0	0.0165	0.138	2098
Organismal Systems; Immune system	Hematopoietic_cell_lineage	71	↑	0.4701	1.7693	0	0.0285	0.322	2579
Organismal Systems; Immune system	Leukocyte_transendothelial_ migration	83	↑	0.5695	2.2054	0	0	0	1040
Organismal Systems; Immune system	Natural_killer_cell_mediated_ cytotoxicity	85	↑	0.4292	1.6933	0.002	0.0443	0.565	953
Organismal Systems; Immune system	NOD_like_receptor_signaling_ pathway	37	↑	0.5096	1.6716	0.008	0.043	0.637	866
Organismal Systems; Immune system	Platelet_activation	89	↑	0.4362	1.7036	0.0057	0.0413	0.521	2084
Organismal Systems; Immune system	T_cell_receptor_signaling_ pathway	85	↑	0.399	1.5374	0.0101	0.0846	0.952	2424
Organismal Systems; Immune system	Toll_like_receptor_signaling_ pathway	74	↑	0.4847	1.8345	0	0.0167	0.172	956
Cellular Processes; Transport and catabolism	Peroxisome	49	↓	-0.6918	-2.4338	0	0	0	750
Genetic Information Processing; Folding, sorting and degradation	Proteasome	40	↓	-0.5004	-1.6498	0	0.026	0.689	2544
Genetic Information Processing; Transcription	Spliceosome	64	↓	-0.4736	-1.7389	0.002	0.0118	0.372	2163
Genetic Information Processing; Translation	mRNA_surveillance_pathway	50	↓	-0.4534	-1.5642	0.0257	0.0473	0.943	2137
Genetic Information Processing; Translation	Ribosome	89	↓	-0.4538	-1.7556	0	0.0109	0.315	2150
Human Diseases; Cancers	Chemical_carcinogenesis	48	↓	-0.4858	-1.7097	0.004	0.0153	0.457	1592
Human Diseases; Endocrine and metabolic diseases	Non_alcoholic_fatty_liver_ disease _(NAFLD)	117	↓	-0.4532	-1.8342	0	0.0054	0.138	2025
Human Diseases; Neurodegenerative diseases	Alzheimer's_disease	127	↓	-0.4411	-1.8064	0	0.0072	0.193	2025
Human Diseases; Neurodegenerative diseases	Huntington's_disease	123	↓	-0.5766	-2.379	0	0	0	2180

Human Diseases; Neurodegenerative diseases	Parkinson's_disease	95	↓	-0.5766	-2.2646	0	0	0	2180
Human Diseases; Substance dependence	Alcoholism	84	↓	-0.388	-1.5042	0.0134	0.0711	0.987	1180
Metabolism; Amino acid metabolism	Arginine_and_proline_metabolism	35	↓	-0.5391	-1.7555	0.0021	0.0104	0.315	806
Metabolism; Amino acid metabolism	Glycine,_serine_and_threonine_ metabolism	26	↓	-0.5175	-1.5715	0.0259	0.0458	0.931	1151
Metabolism; Amino acid metabolism	Histidine_metabolism	10	↓	-0.6671	-1.6042	0.0323	0.0369	0.853	1016
Metabolism; Amino acid metabolism	Lysine_biosynthesis	2	↓	-0.9291	-1.3862	0.0367	0.1392	1	658
Metabolism; Amino acid metabolism	Lysine_degradation	24	↓	-0.7478	-2.2464	0	0	0	807
Metabolism; Amino acid metabolism	Tryptophan_metabolism	25	↓	-0.7103	-2.1176	0	0.0001	0.001	1055
Metabolism; Amino acid metabolism	Valine,_leucine_and_isoleucine_ degradation	33	↓	-0.7719	-2.4935	0	0	0	874
Metabolism; Biosynthesis of other secondary metabolites	Caffeine_metabolism	5	↓	-0.7778	-1.495	0.0497	0.0743	0.99	1305
Metabolism; Carbohydrate metabolism	Ascorbate_and_aldarate_ metabolism	9	↓	-0.7204	-1.6508	0.021	0.0265	0.688	658
Metabolism; Carbohydrate metabolism	Butanoate_metabolism	13	↓	-0.6951	-1.7504	0.0099	0.0108	0.333	2639
Metabolism; Carbohydrate metabolism	Citrate_cycle_(TCA_cycle)	28	↓	-0.6387	-1.9422	0	0.0013	0.026	2001
Metabolism; Carbohydrate metabolism	Glyoxylate_and_dicarboxylate_ metabolism	17	↓	-0.6356	-1.775	0.0019	0.0091	0.256	1935
Metabolism; Carbohydrate metabolism	Propanoate_metabolism	17	↓	-0.7047	-1.93	0.0019	0.0014	0.032	1014
Metabolism; Carbohydrate metabolism	Pyruvate_metabolism	26	↓	-0.6133	-1.8485	0.0019	0.0046	0.112	1151
Metabolism; Energy metabolism	Oxidative_phosphorylation	88	↓	-0.6316	-2.4269	0	0	0	2009
Metabolism; Lipid metabolism	alpha_Linolenic_acid_metabolism	12	↓	-0.6595	-1.6097	0.0223	0.0358	0.836	535
Metabolism; Lipid metabolism	Biosynthesis_of_unsaturated_ fatty_acids	11	↓	-0.8515	-2.1042	0	0.0001	0.002	865

Metabolism; Lipid metabolism	Fatty_acid_degradation	27	↓	-0.7729	-2.3228	0	0	0	807
Metabolism; Lipid metabolism	Fatty_acid_elongation	14	↓	-0.7618	-1.9915	0	0.0007	0.014	865
Metabolism; Lipid metabolism	Linoleic_acid_metabolism	22	↓	-0.6193	-1.8221	0.0038	0.0061	0.16	1796
Metabolism; Lipid metabolism	Primary_bile_acid_biosynthesis	11	↓	-0.8021	-2.0128	0.0019	0.0005	0.008	750
Metabolism; Lipid metabolism	Steroid_hormone_biosynthesis	41	↓	-0.5802	-1.937	0	0.0013	0.029	939
Metabolism; Metabolism of cofactors and vitamins	One_carbon_pool_by_folate	7	↓	-0.8535	-1.8042	0.002	0.0069	0.194	1103
Metabolism; Metabolism of cofactors and vitamins	Porphyrin_and_chlorophyll_ metabolism	18	↓	-0.5945	-1.6389	0.0114	0.0281	0.737	1015
Metabolism; Metabolism of cofactors and vitamins	Retinol_metabolism	34	↓	-0.6783	-2.2113	0	0	0	641
Metabolism; Metabolism of other amino acids	beta_Alanine_metabolism	17	↓	-0.7418	-2.0589	0	0.0003	0.005	1016
Metabolism; Metabolism of other amino acids	Selenocompound_metabolism	11	↓	-0.7299	-1.7737	0.0061	0.0089	0.259	1986
Metabolism; Xenobiotics biodegradation and metabolism	Drug_metabolism_cytochrome_ P450	29	↓	-0.508	-1.5755	0.0246	0.0456	0.924	1250
Metabolism; Xenobiotics biodegradation and metabolism	Metabolism_of_xenobiotics_by_ cytochrome_P450	34	↓	-0.4791	-1.5454	0.0366	0.0532	0.968	1220
Organismal Systems; Endocrine system	Ovarian_steroidogenesis	43	↓	-0.4968	-1.6997	0.0095	0.0166	0.506	225
Organismal Systems; Endocrine system	PPAR_signaling_pathway	54	↓	-0.5441	-1.9014	0	0.0022	0.054	901

1. The complete gene expression profile in DEN-treated *bid* -deficient livers for 4-6 months were subjected to GSEA using KEGG pathway desgination.
2. Size: Number of genes in the gene set after filtering out these genes not in the expression dataset.
3. ES: Enrichment score for the gene set, the degree to which this gene set is overrepresented at the top or bottom of the ranked list of genes in the expression dataset.
4. NES: Normalized enrichment score, the enrichment score for the gene set after it has been normalized across analyzed gene sets.
5. NOR p-value: Norminal p value, the statistical significance of the enrichment score. It is not adjusted for gene set size or multiple hypothesis testing.
6. FRD q-value: False discovery rate, the estimated probability that the nromalized enrichment score representes a false positive finding.
7. FWER p-value: Familywise-error rate, a more conservatively estimated probabillity that the normalzied enrichment score represents a false positive findnig.
8. RANK AT MAX: The positiion in the ranked list at which the maximum enrichment socre occurred.
9. For the up-regulated pathways, those related to immune response/inflammation are shown in red font, and those related to cancer and growth regulation are shown in blue font.

For the down-regulated pathways, those related to amino acids metabolism are shown in red font, and those related to other types of metabolisms are shown in blue font.
