# Supplementary material for: Gene Expression Analysis Indicates Divergent Mechanisms in DEN-Induced Carcinogenesis in Wild Type and Bid-Deficient Livers
Source: PLoS One. 2016 May 19;11(5):e0155211. doi: 10.1371/journal.pone.0155211 (PMC4873180; doi:10.1371/journal.pone.0155211)
Supplement: S14 Table — (PDF) [file pone.0155211.s014.pdf]

S14 Table. Pathway analysis of the gene expression profile in *bid* -deficient mice treated with DEN for 4-6 months

Class	Pathway	Size	Change	ES	NES	NOM p-val	FDR q-val	FWER p-val	RANK AT MAX
Genetic Information Processing; Folding, sorting and degradation	Ubiquitin_mediated_proteolysis	80	↑	0.3837	1.5463	0.0065	1	0.961	1433
Metabolism; Amino acid metabolism	Glycine,_serine_and_threonine_metabolism	26	↑	0.5033	1.5812	0.0181	1	0.918	3262
Metabolism; Amino acid metabolism	Tyrosine_metabolism	25	↑	0.475	1.517	0.0381	1	0.992	1282
Organismal Systems; Immune system	Cytosolic_DNA_sensing_pathway	36	↑	0.4287	1.472	0.037	1	1	686
Organismal Systems; Nervous system	Glutamatergic_synapse	69	↑	0.3419	1.3579	0.0487	1	1	2333
Cellular Processes; Cellular commiunity	Adherens_junction	61	↓	-0.3381	-1.3873	0.0475	0.8085	0.998	1502
Cellular Processes; Transport and catabolism	Phagosome	107	↓	-0.3316	-1.5075	0.0107	0.6064	0.968	2898
Environmental Information Processing; Signaling molecules and interaction	Cell_adhesion_molecules_(CAMs)	93	↓	-0.3039	-1.3338	0.048	0.7435	1	2610
Genetic Information Processing; Replication and repair	Base_excision_repair	22	↓	-0.5374	-1.7276	0.0047	0.6221	0.447	1382
Human Diseases; Endocrine and metabolic diseases	Type_I_diabetes_mellitus	41	↓	-0.4102	-1.5728	0.0134	0.7442	0.877	1184
Human Diseases; Infectious diseases	African_trypanosomiasis	24	↓	-0.5087	-1.6453	0.0132	0.6342	0.704	2246
Human Diseases; Infectious diseases	Staphylococcus_aureus_infection	36	↓	-0.3935	-1.4217	0.0371	0.9285	0.996	1849
Metabolism; Metabolism of cofactors and vitamins	Nicotinate_and_nicotinamide_metabolism	7	↓	-0.6919	-1.5586	0.0416	0.5005	0.904	1654
Organismal Systems; Excretory system	Collecting_duct_acid_secretion	17	↓	-0.5299	-1.5639	0.0357	0.6012	0.894	741

1. The complete gene expression profile in DEN-treated *bid* -deficient livers for 4-6 months were subjected to GSEA using KEGG pathway desgination.
2. Size: Number of genes in the gene set after filtering out these genes not in the expression dataset.
3. ES: Enrichment score for the gene set, the degree to which this gene set is overrepresented at the top or bottom of the ranked list of genes in the expression dataset.
4. NES: Normalized enrichment score, the enrichment score for the gene set after it has been normalized across analyzed gene sets.
5. NOR p-value: Norminal p value, the statistical significance of the enrichment score. It is not adjusted for gene set size or multiple hypothesis testing.

6. FRD q-value: False discovery rate, the estimated probability that the nromalized enrichment score representes a false positive finding.
7. FWER p-value: Familywise-error rate, a more conservatively estimated probabillity that the normalzied enrichment score represents a false positive findnig.
8. RANK AT MAX: The positiion in the ranked list at which the maximum enrichment socre occurred.
9. For the up-regulated pathways, those related to immune response/inflammation are shown in red font, and those related to cancer and growth regulation are shown in blue font.
For the down-regulated pathways, those related to amino acids metabolism are shown in red font, and those related to other types of metabolisms are shown in blue font.
