# Supplementary material for: Gene Expression Analysis Indicates Divergent Mechanisms in DEN-Induced Carcinogenesis in Wild Type and Bid-Deficient Livers
Source: PLoS One. 2016 May 19;11(5):e0155211. doi: 10.1371/journal.pone.0155211 (PMC4873180; doi:10.1371/journal.pone.0155211)
Supplement: S15 Table — (PDF) [file pone.0155211.s015.pdf]

S15 Table. Pathway analysis of the gene expression profile in *bid* -deficient mice treated with DEN for 10-12 months

Class	Pathway	Size	Change	ES	NES	NOM p-val	FDR q-val	FWER p-val	RANK AT MAX
Cellular Processes; Cellular commiunity	Focal_adhesion	143	↑	0.3491	1.5416	0.0054	0.2606	0.975	1057
Cellular Processes; Transport and catabolism	Endocytosis	139	↑	0.3149	1.3812	0.0336	0.4402	1	1556
Environmental Information Processing; Signal transduction	ErbB_signaling_pathway	65	↑	0.3738	1.4513	0.0338	0.3705	1	796
Environmental Information Processing; Signaling molecules and interaction	ECM_receptor_interaction	58	↑	0.4311	1.6127	0.0126	0.3190	0.878	1167
Human Diseases; Cancers	Colorectal_cancer	52	↑	0.4466	1.6645	0.0057	0.4748	0.752	1504
Human Diseases; Cancers	Non_small_cell_lung_cancer	42	↑	0.4083	1.4362	0.0470	0.3711	1	1873
Human Diseases; Cancers	Small_cell_lung_cancer	64	↑	0.4275	1.6276	0.0088	0.3960	0.84	1057
Human Diseases; Cardiovascular diseases	Dilated_cardiomyopathy	64	↑	0.3834	1.4968	0.0239	0.3022	0.994	1678
Human Diseases; Cardiovascular diseases	Hypertrophic_cardiomyopathy_(HCM)	57	↑	0.4433	1.6570	0.0076	0.3795	0.777	1408
Human Diseases; Cardiovascular diseases	Viral_myocarditis	57	↑	0.4082	1.5357	0.0257	0.2545	0.976	1556
Human Diseases; Endocrine and metabolic dis[illegible]	Type_I_diabetes_mellitus	41	↑	0.4510	1.5961	0.0182	0.2317	0.915	1828
Human Diseases; Immune diseases	Graft_versus_host_disease	36	↑	0.4182	1.4497	0.0318	0.3565	1	1556
Human Diseases; Infectious diseases	Hepatitis_B	106	↑	0.3589	1.5339	0.0123	0.2434	0.977	1408
Human Diseases; Infectious diseases	Herpes_simplex_infection	123	↑	0.3733	1.6051	0.0017	0.2626	0.891	1602
Human Diseases; Infectious diseases	Influenza_A	116	↑	0.3658	1.5436	0.0036	0.2755	0.973	1387
Human Diseases; Infectious diseases	Measles	92	↑	0.3822	1.5574	0.0131	0.2662	0.96	1455
Metabolism; Amino acid metabolism	Valine,_leucine_and_isoleucine_ biosynthesis	3	↑	0.9325	1.6166	0.0102	0.3594	0.869	155

Metabolism; Metabolism of cofactors and vitamins	One_carbon_pool_by_folate	7	↑	0.7190	1.6102	0.0383	0.2842	0.88	618
Metabolism; Metabolism of cofactors and vitamins	Pantothenate_and_CoA_biosynthesis	6	↑	0.8189	1.7663	0	0.544	0.405	155
Organismal Systems; Development	Osteoclast_differentiation	96	↑	0.3824	1.5736	0.0072	0.2564	0.947	1978
Organismal Systems; Immune system	Antigen_processing_and_presentation	55	↑	0.4657	1.7036	0	0.4869	0.61	1603
Organismal Systems; Immune system	Toll_like_receptor_signaling_pathway	74	↑	0.3940	1.5989	0.0073	0.2489	0.911	1462
Genetic Information Processing; Translation	Aminoacyl_tRNA_biosynthesis	21	↓	-0.4836	-1.4867	0.04	0.771	0.982	2799
Metabolism; Carbohydrate metabolism	Citrate_cycle_(TCA_cycle)	28	↓	-0.4444	-1.4938	0.0467	0.9101	0.979	3600
Metabolism; Carbohydrate metabolism	Glycolysis_/_Gluconeogenesis	47	↓	-0.4025	-1.5118	0.0184	1	0.966	2804
Metabolism; Lipid metabolism	Steroid_biosynthesis	14	↓	-0.6217	-1.7143	0.0042	0.2529	0.414	1923
Metabolism; Metabolism of cofactors and vitamins	Lipoic_acid_metabolism	1	↓	-0.9782	-1.2998	0.0374	1	1	203
Organismal Systems; Excretory system	Proximal_tubule_bicarbonate_reclamation	13	↓	-0.6996	-1.9171	0	0.0453	0.047	861

1. The complete gene expression profile in DEN-treated *bid*-deficient livers for 4-6 months were subjected to GSEA using KEGG pathway desgination.
2. Size: Number of genes in the gene set after filtering out these genes not in the expression dataset.
3. ES: Enrichment score for the gene set, the degree to which this gene set is overrepresented at the top or bottom of the ranked list of genes in the expression dataset.
4. NES: Normalized enrichment score, the enrichment score for the gene set after it has been normalized across analyzed gene sets.
5. NOR p-value: Norminal p value, the statistical significance of the enrichment score. It is not adjusted for gene set size or multiple hypothesis testing.
6. FRD q-value: False discovery rate, the estimated probability that the nromalized enrichment score representes a false positive finding.
7. FWER p-value: Familywise-error rate, a more conservatively estimated probabillity that the normalzied enrichment score represents a false positive findnig.
8. RANK AT MAX: The positiion in the ranked list at which the maximum enrichment socre occurred.
9. For the up-regulated pathways, those related to immune response/inflammation are shown in red font, and those related to cancer and growth regulation are shown in blue font.
For the down-regulated pathways, those related to amino acids metabolism are shown in red font, and those related to other types of metabolisms are shown in blue font.
