# Supplementary material for: Gene Expression Analysis Indicates Divergent Mechanisms in DEN-Induced Carcinogenesis in Wild Type and Bid-Deficient Livers
Source: PLoS One. 2016 May 19;11(5):e0155211. doi: 10.1371/journal.pone.0155211 (PMC4873180; doi:10.1371/journal.pone.0155211)
Supplement: S16 Table — (PDF) [file pone.0155211.s016.pdf]

S16 Table. List of qRT-PCR primers

Gene	Forward Primer	Reverse Primers
**Adh4**	GTAGACTCTGTCCCAAACCTG	AAGGTCAGGATTGTTCGGATG
**β-actin**	GACGGCCAGGTCATCACTATTG	AGGAAGGCTGGAAAAGAGCC
**C1qb**	GGGTTCCTGGCTCTGATG	CTCCAAACTCACCAAGGTCTC
**Cyp2B9**	CCTCGACTACATTGCCCATAG	TTCTGGTGATGGAACTCTGTG
**Cyp7B1**	CAGTCCACTTCACCAGAGAAC	CACAGCCTCAGAACCTCAAG
**Egfr**	CTCCATGCTTTCGAGAACCTAG	ATGATCACATCCCCATCACTG
**Fos**	TCCTTACGGACTCCCCAC	CTCCGTTTCTCTTCCTCTTCAG
**Glo1**	ATTGCCGTTCCTGATGTCTAC	GAATCTCAATCCAGTAGCCGTC
**Gstm3**	ACACTGGGCTATTGGAACAC	TGGCTTCGGTCAAAGTTGG
